# Modification of Amorphous Mesoporous Zirconia Nanoparticles with Bisphosphonic Acids: A Straightforward Approach for Tailoring the Surface Properties of the Nanoparticles

**DOI:** 10.1002/chem.202103354

**Published:** 2021-11-17

**Authors:** Khohinur Hossain, Luca Florean, Anna Del Tedesco, Elti Cattaruzza, Marco Geppi, Silvia Borsacchi, Patrizia Canton, Alvise Benedetti, Pietro Riello, Alessandro Scarso

**Affiliations:** ^1^ Dipartimento di Scienze Molecolari e Nanosistemi Università Ca' Foscari Venezia Via Torino 155 30172 Venezia Mestre Italy; ^2^ Dipartimento di Chimica e Chimica Industriale Università di Pisa via G. Moruzzi 13 56124 Pisa Italy; ^3^ ICCOM CNR s.s. Pisa via G. Moruzzi, 1 56124 Pisa Italy

**Keywords:** bisphosphonic acids, colloidal systems, MAS NMR, surface functionalization, zirconia nanoparticles

## Abstract

The use of readily prepared bisphosphonic acids obtained in few steps through a thio‐Michael addition of commercially available thiols on tetraethyl vinylidenebisphosphonate enables the straightforward surface modification of amorphous mesoporous zirconia nanoparticles. Simple stirring of the zirconia nanoparticles in a buffered aqueous solution of the proper bisphosphonic acid leads to the surface functionalization of the nanoparticles with different kinds of functional groups, charge and hydrophobic properties. Formation of both chemisorbed and physisorbed layers of the bisphosphonic acid take place, observing after extensive washing a grafting density of 1.1 molecules/nm^2^ with negligible release in neutral or acidic pH conditions, demonstrating stronger loading compared to monophosphonate derivatives. The modified nanoparticles were characterized by IR, XPS, ζ‐potential analysis to investigate the loading of the bisphosphonic acid, FE‐SEM to investigate the size and morphologies of the nanoparticles and ^31^P and ^1^H MAS NMR to investigate the coordination motif of the phosphonate units on the surface. All these analytical techniques demonstrated the strong affinity of the bisphosphonic moiety for the Zr(IV) metal centers. The functionalization with bisphosphonic acids represents a straightforward covalent approach for tailoring the superficial properties of zirconia nanoparticles, much straightforward compared the classic use of trisalkoxysilane or trichlorosilane reagents typically employed for the functionalization of silica and metal oxide nanoparticles. Extension of the use of bisphosphonates to other metal oxide nanoparticles is advisable.

## Graphical abstract

Bisphosphonic acids provide a straightforward method for the covalent modification of the surface of mesoporous zirconia nanoparticles, with stronger affinity with respect to monophosphonate compounds. The modular synthesis of the bisphosphonic acids based on thio‐Michael addition reaction on tetraethyl vinylidenebisphosphonate ester enables the preparation of zirconia nanoparticles with specific functional groups or charges on the surface.

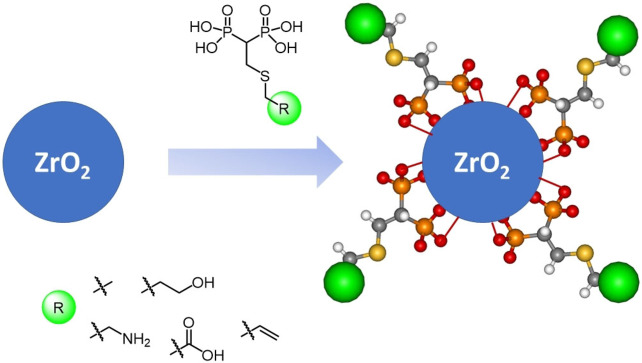

## Introduction

The development of new hybrid organic‐inorganic nanosystems is a cutting‐edge field of research in particular for high potential biomedical applications.^[1]^ Specifically, the interaction between the organic ligands and the inorganic nanoparticles needs to be thoroughly investigated and tailored in order to achieve stability and to impart specific properties to the nano‐assemblies.^[2]^

Zirconia is a very interesting material for nano‐technological applications,^[3]^ in particular for biomedical purposes^[4]^ due to its biocompatibility^[5]^ derived by a large inertness and high thermal, mechanical, and chemical stability^[6]^ except at very high pH conditions. Recently, among other methods,^[7]^ an innovative procedure for the preparation of amorphous zirconia nanoparticles (ZrNPs) based on the template synthesis starting from Zr(O*n*‐Pr)_4_ in the presence of hexadecylamine as templating unit has been reported for theranostic applications.^[8]^ ZrNPs produced with this method are characterized by average diameter of 200 nm, surface area of 187.4±0.6 m^2^/g and pore diameters in the range 3.4–6.6 nm, showing biocompatibility and thermal and mechanical stability till 200 °C which are highly suitable for biomedical applications. Recently we demonstrated the promising drug loading and release properties of this material towards a series of active pharmaceutical ingredients like *N*‐acetyl cysteine, vancomycin, ibuprofen and nitrofurantoin.^[9]^

Similarly to silica NPs, ZrNPs are subjected to extensive study and functionalization to modify the surface properties of these materials. The surface chemistry on zirconia^[10]^ is not comparable to that of silica in particular due to the decreased chemical stability of the silane bond from Zr−O−Si−R with respect to Si−O−Si−R. In fact, the employment of organosilane chemistry for the functionalization of zirconia provides limited applications.^[11]^ Because of this, one of the most common methods employed for zirconia functionalization is based on the deposition of a silica‐coating layer to which a further silane application^[12]^ is performed to tailor the surface properties of the NPs. It is worth to notice that the use of silane derivatives implies a condensation reaction with release of alcohol or halides by‐products.^[13]^ Because of the decreasing chemical stability of the silane bond from Si−O−Si‐R≥Zr−O−Si−R>Ti−O−Si−R, the use of organosilanes for the general functionalization of transition metal oxide provides limited applicability.^[14]^

It is well known that zirconia, thanks to its surface structure,^[15]^ can efficiently interact with phosphate and phosphonate‐based ligands,[^16^, ^17^] as evidenced also by the application of zirconia to remove phosphates from water.^[18]^ More in detail, in the literature some examples of zirconia functionalization with mono‐phosphonate[^19^, ^20^, ^21^] containing species have been reported. Other metal oxides have been recently demonstrated to interact with mono‐phosphonates like aluminum oxide^[22]^ or titanium oxide^[23]^ for which the comparison of the binding with other typical ligands like catechol, carboxylates and others has been investigated.^[24]^

**Scheme 1:**
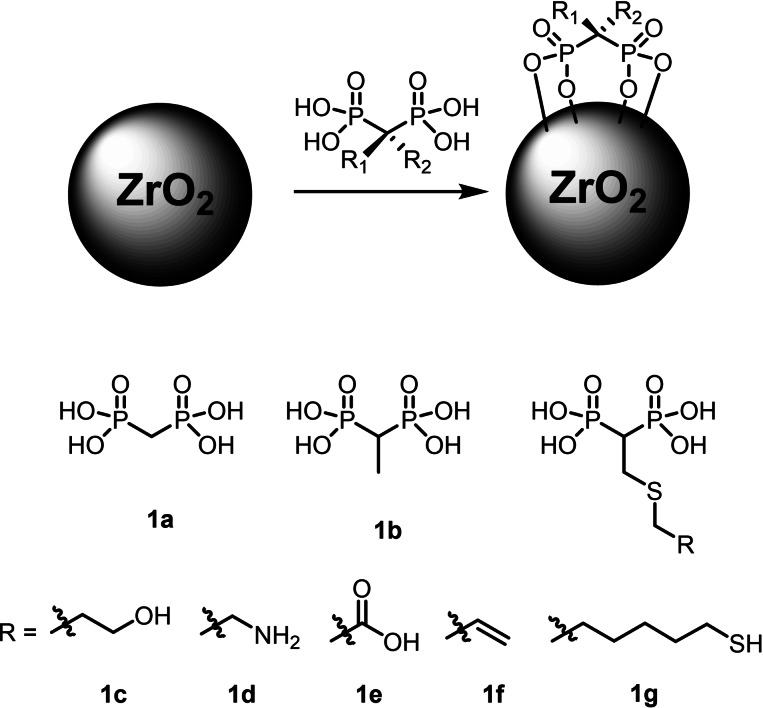
Functionalization of amorphous mesoporous ZrNPs with BPs **1 a**‐**g** characterized by different length, polarity of the side chain and presence of specific functional groups.

**Scheme 2:**
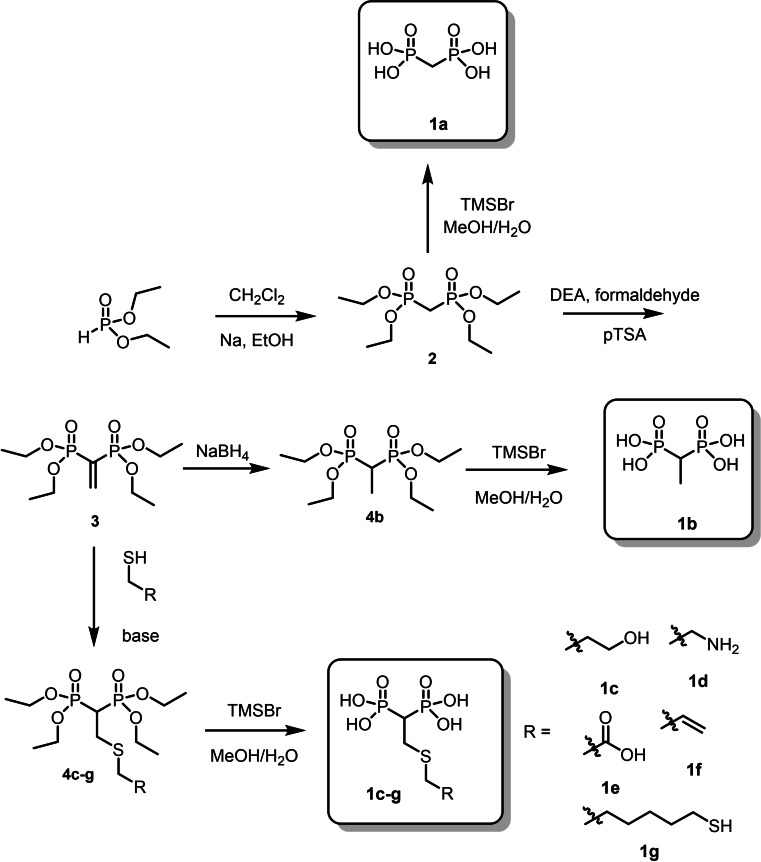
Synthetic approach for the preparation of the BPs **1 a**–**g** characterized by different substituents on the central C atom.

Bisphosphonic acids (BPs, **1**, Scheme 1) are a well‐known class of organic molecules that, due to their affinity for hydroxyapatite as mineral constituent of bones enabled by the chelating properties of the two phosphonate units for calcium, find application for the contrast of osteoporosis and other bone diseases.[^25^, ^26^] Moreover, the presence of the non‐hydrolysable P−C−P bond ensures the chemical stability of the molecule. BPs can be prepared with a series of synthetic approaches.^[27]^ We considered in particular the modification of vinylidenebisphosphonate ester **3** as an important scaffold obtained by reaction of diethyl phosphite with methylene chloride via formation of methylenebisphosphonate ester **2** (Scheme 2). **3** is a versatile electrophile that through Michael^[28]^ or hetero‐Michael^[29]^ addition reactions enable the preparation of a wide range^[30]^ of potential drug candidates to contrast osteoporosis and bone diseases.

In the present contribution we propose the modification of the surface properties of ZrNPs based on the employment of a family of BPs as derivatizing species (Scheme 1). The latter ligands can be tailored with specific functional groups in the side chain such as amino, carboxylic acid, alcohol, alkene and others for possible further derivatization, and their affinity with ZrNPs turned out to be very high, with formation of both chemisorbed and physisorbed layers with higher stability compared to monophosphonates. The functionalization was obtained by dispersing the ZrNPs with an aqueous solution of the desired BPs under buffered conditions. This led to a wide range of surface modified NPs with enhanced colloidal stability due to the presence of negative charges on the surface over a wide range of pH, as demonstrated by ζ‐potential measurements. The described method represents a step forward with respect to the typical approach for zirconia functionalization based on the formation of silica shells or the direct use of *tris*‐alkoxysilane or trichlorosilane derivatization methods.^[31]^

## Results and Discussion

### Synthesis of BPs 1 a–g

The proposed family of BPs were all prepared following or adapting known procedures. More in detail, the typical intermediate methylene bisphosphonate tetraethyl‐ester **2** (Scheme 2) was prepared from diethyl‐phosphite with dichloromethane and further elongated with formaldehyde leading to the vinylidene bisphosphonate ester **3**. To this building block, a series of thiols could be added exploiting the use of triethylamine as base catalyst^[32]^ forming **4 c**–**g**. Alternatively, **3** could be reduced with sodium borohydride obtaining **4 b**. The final BPs with free phosphonic units **1 a**–**g** were obtained by ethyl ester deprotection with trimethyl silyl bromide (TMSBr) followed by hydrolysis with water in methanol (Scheme 2).^[33]^

**Scheme 3:**
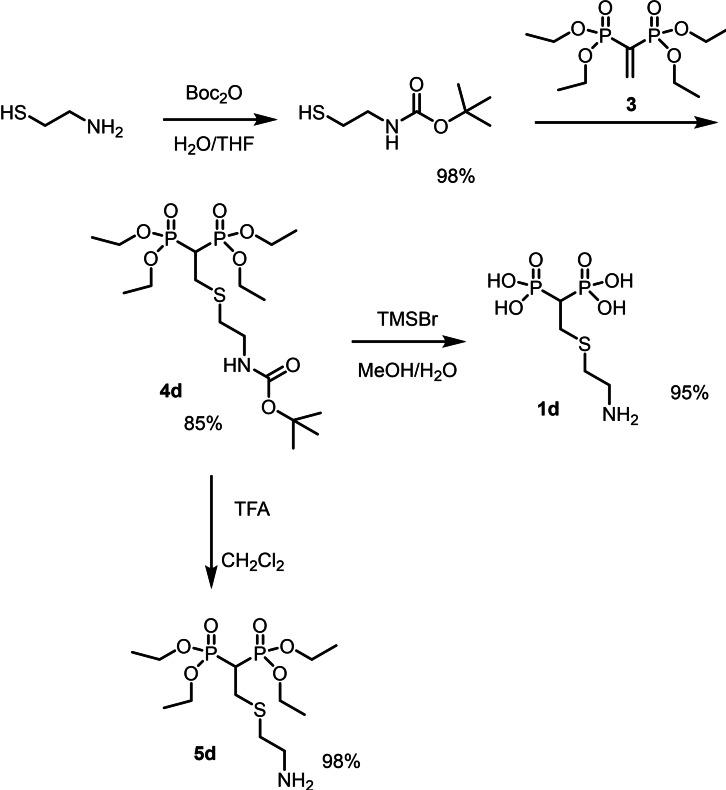
Protection of 2‐aminoethanethiol with Boc and subsequent thio‐Michael addition to **3** leading to the Boc protected tetraethylester derivative **4 d**, further transformed into the final BP acid **1 d** or into the free amino BP **5 d** that could be further functionalized on the amino moiety.

Yields for the BPs **1 a**–**g** from the common precursor **3** were usually good and the reactions could be easily scaled up on the order of hundreds of milligrams. All products were characterized by ^1^H, ^31^P, ^13^C NMR and some 2D NMR experiments as reported in the Supporting Information. In the case of the amino substituted BP **1 d** the commercially available 2‐aminoethanthiol turned out to react with **3** forming a mixture of N and S *β*‐substituted BP esters. Because of this it was necessary to preliminarily protect the amino functionality of 2‐aminoethanthiol with Boc and then the product was further reacted with **3** forming the desired product (Scheme 3).

Treatment of **4 d** with TMSBr led to the formation of the BPs **1 d** with free terminal amino group due to the concomitant Boc removal. Alternatively, product **4 d** could be selectively deprotected on the amino group by treatment with trifluoroacetic acid (TFA) forming the corresponding free amino‐BP **5 d** in good yield.

**Scheme 4:**
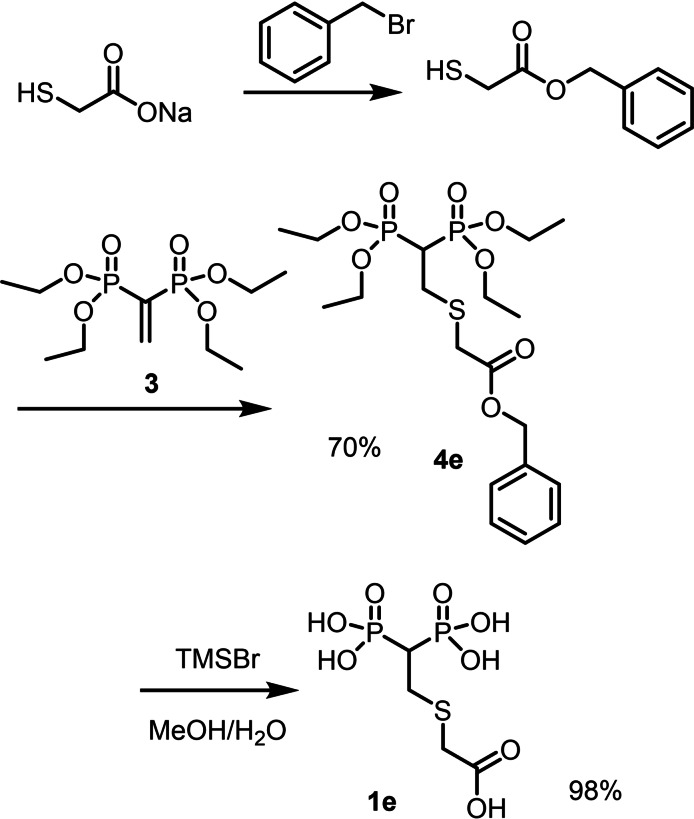
Protection of sodium thioglycolate with benzyl bromide and subsequent thio‐Michael addition to **3** leading to the derivative **4 e**, further transformed into the final BP acid **1 e**.

For the synthesis of **4 e**, the reaction of sodium thioglycolate with **3** did not provide the desired product even using polar solvents like DMSO or DMF, observing in all cases a mixture of products derived by both O and S attack on the *β* carbon atom of **3**. To selectively protect the carboxylic unit, sodium thioglycolate was reacted in DMF with benzyl bromide under inert atmosphere forming the corresponding benzyl ester with free thiol moiety. The latter product was reacted with **3** directly without isolation, forming the desired thio‐Michael addition product **4 e** that was eventually deprotected with TMSBr to form the free BP acid **1 e** (Scheme 4).

### Synthesis of ZrNPs

Amorphous mesoporous zirconia nanoparticles ZrNPs were successfully prepared by a neutral surfactant assisted sol‐gel method in the presence of NaF.^[8]^ It is worth to note that in order to obtain amorphous ZrNPs with a large surface area, while preserving the porosity, the surfactant was removed at 120 °C in a vacuum extraction process rather than by calcination. The nanoparticles obtained by this method are monodispersed amorphous products characterized by average mean diameter of 200±50 nm, superficial area of 187 m^2^/g and pore diameter distribution of 5.1±1.5 nm.^[34]^ The isotherms display the type IV profile with a H1 hysteresis loop (according to the IUPAC classification),^[35]^ which is typical for mesoporous materials. For this reason, these ZrNPs have the potentiality to host several types of molecules.

#### Interaction of 1 a with ZrNPs

**Figure 1:**
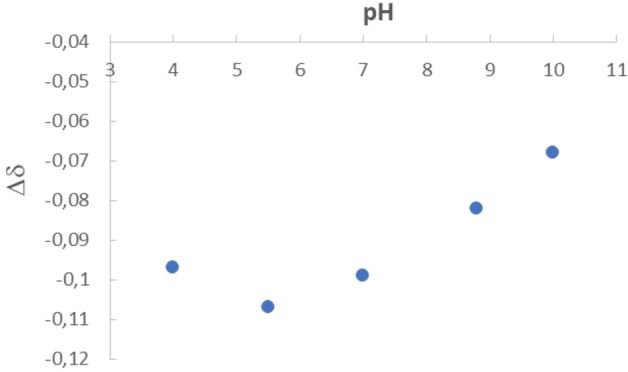
Average Δδ values for the ^1^H NMR resonances of the buffer species upon addition of ZrNPs.

To investigate in detail the affinity of the BPs with ZrNPs we initially focused the attention on the smallest BP derivative **1 a** monitoring over time the decrease of its concentration in buffered aqueous solution containing a fixed amount of ZrNPs through quantitative ^1^H NMR analysis in solution. Preliminarily, control experiments were also performed to investigate the possible interaction of the different buffer species with ZrNPs. It was observed that 25 mM acetate pH 4.0, MES pH 5.5, HEPES pH 7.0, and CAPS pH 10.0 buffers did not show decrease of the concentration of the buffering species larger than 5 % with respect to the initial concentration (see Supporting Information) which is a value that is also comparable with the uncertainty of the quantitative NMR determination. Differently, TRIZMA buffer for pH 8.8 showed a slightly larger 10 % decrease of concentration after interaction with ZrNPs. This is likely due to the neutral polydentate molecular structure of the buffer bearing three alcohol moieties that can bind the surface of the ZrNPs. It is also worth to note that a shift of the proton resonances for all the buffers was observed with negative Δδ in all cases (Figure 1). With the exception of the acetate buffer, an almost linear trend was observed for the Δδ with the pH of the solution, indicative of a shielding effect on the buffer species provided by the ZrNPs. Considering that the ZrNPs are characterized by the presence of positive charges at low pH values (see later), one would expect a de‐shielding effect on the buffer resonances imparted by the NPs at low pH values. A possible explanation for this phenomenon could be proposed considering that at acidic pH values the positively charged ZrNPs in water are surrounded by a negatively charged Stern layer and it is within this electron rich surface that the acetate molecules experience an overall shielding effect. Moving to neutral and basic pH conditions, the ZrNPs tend to become negatively charged, and a consequent decreased shielding effect is observed for MES, HEPES, TRIZMA, and CAPS.

Initial tests to monitor the affinity of **1 a** with ZrNPs were carried out stirring at 600 rpm for different time periods 50 mg of ZrNPs with a series of 5 mL in D_2_O solutions containing 5 mM of **1 a** and 25 mM buffer systems in the pH range 4.0–8.8. Tests at pH 10 were not further investigated confirming the chemical instability of the BPs over time at that pH value.^[8]^ After 15, 30, 60 and 120 minutes the samples were centrifuged and the surnatant solution was quantitatively analyzed by ^1^H NMR.

**Figure 2:**
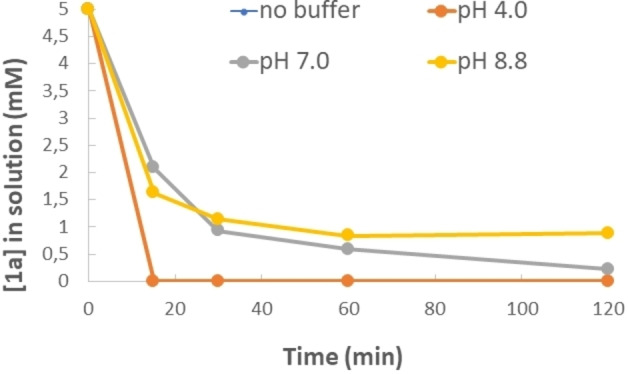
Concentration profile in the surnatant solution of **1 a** at different buffered pH values monitored by quantitative ^1^H NMR after interaction with ZrNPs over time. [**1 a**]_0_=5 mM 5 mL, ZrNPs 50 mg. No buffer and pH 4 data overlap.

The area of the triplet at about 2.24 ppm attributed to the methylene unit of **1 a** was monitored over time (see Supporting Information). In water (unbuffered solution pH 2.0) and at pH 4.0, the uptake of **1 a** by the ZrNPs from the aqueous solution was quantitative within few minutes (superimposed curves in Figure 2). A further increase in the pH led to a decreased affinity observing after 1 h about 88 % of binding of **1 a** at pH 7.0 and slightly lower binding (83 %) at basic pH 8.8. To determine the maximum loading of **1 a** on the ZrNPs under acidic conditions at pH 4.0 the experiments were repeated with initial 10 and 25 mM solutions of **1 a** observing 100 % and 70 % loading on 50 mg of ZrNPs, respectively.

Considering the molecular footprint of a single molecule of **1 a** about 42 Å^2^ from molecular modeling and the average surface area of the ZrNPs about 187 m^2^/g determined by nitrogen adsorption‐desorption isotherms (BET equation), it was possible to calculate at pH 4.0 the surface coverage by **1 a** on ZrNPs of about 68 %, 135 % and 237 %, respectively when using the 5, 10 and 25 mM solution of **1 a** for the loading. Such very high loading values can be interpreted considering a combination of chemisorption directly on the ZrNPs surface and a physisorption superficial phenomenon that takes place on a large extent, in which further layers of **1 a** are present on the surface by interaction through H‐bonding with the chemisorbed **1 a** molecules.

In order to determine the grafting density of the BPs due to chemisorption on the surface of the ZrNPs, TGA experiments on bare ZrNPs and with **1 a**–**f** were carried. Unfortunately the presence of residual templating agent in the bare ZrNPs and the tendency of phosphonate containing compounds to form new solid phases with zirconium under thermal treatment, led to inconsistent mass loss data. Moreover, the thermal treatment above 300 °C is known to cause a crystallization process with an abrupt decrement of the porosity,^[8]^ which is against the purpose of the investigation of these ZrNPs for possible future theranostic applications. Therefore, we performed a series of CHNS elemental analyses on samples of bare ZrNPs as well as ZrNPs loaded with **1 a**. The ZrNPs show the presence of C, H and N which can be ascribed to the residual presence of the templating hexadecylamine used as templating unit for the synthesis of the porous ZrNPs which was not completely removed by the vacuum extraction process (Table 1, entry 1). The samples of ZrNPs loaded with **1 a** and thoroughly washed with pure water showed a small increment on the C and H content as a confirmation of the presence of the BP on the surface of the nanoparticles (Table 1, entry 2). Since **1 a** contains only one methylene unit in the structure, its contribution to the overall elemental composition is small with respect to the C and H content arising by the residual hexadecylamine. Due to the small difference in elemental composition observed between bare ZrNPs and those with **1 a** it was not possible to determine the amount of loaded **1 a** with sufficient accuracy. In order to overcome this limitation, we decided to prepare **1 g** as a specific BP characterized by low molecular weight bearing two S atoms. The new compound was obtained similarly to the other derivatives **1 c**–**f** by reaction of **3** with 1,5 pentane dithiol in the presence of triethylamine leading to the protected **4 g** in 98 % yield. The latter was further treated with TMSBr to obtain the final BP **1 g** in the acid form in 96 % yield. S is not present on the bare ZrNPs, therefore **1 g** enabled a more accurate determination of the loading of the BPs. The very small S content present in bare ZrNPs does not come by the reagents used in the synthesis and it is basically comparable to the detection limit of thee technique. The slightly larger % S for Zr NPs **1 a** could be due to the use of sodium sulfate in the synthesis of **1 a**. What is really clear is that the % of S largely changes in the presence of **1 g** due to the presence of two S atoms in the molecular structure of the bisphosphonic acid employed. As reported in Table 1 entry 3, the % of S for the ZrNPs treated with **1 g** was sufficiently high to allow an accurate determination of the number on moles of **1 g** present on the surface. From these data and considering a specific surface area of 187 m^2^/g, a loading of 1.9 μmol/m^2^ which corresponds to a grafting density of 1.1 molecules/nm^2^ was calculated. Considering the molecular footprint of **1 g** comparable to that of **1 a** excluding any contribution by the aliphatic thiol containing side chain on the grafting on ZrNPs, it can be calculated a coverage of the surface of the NP corresponding to 47 %.

**Table 1:** Elemental analysis for the bare ZrNPs and for the samples loaded with **1 a** and the S containing BP **1 g**.

#	Sample	% C	% H	% N	% S
1	ZrNPs	2.90	1.45	0.13	0.03
2	ZrNPs **1 a**	3.27	1.49	0.13	0.09
3	ZrNPs **1 g**	6.33	1.69	0.11	2.09

**Figure 3:**
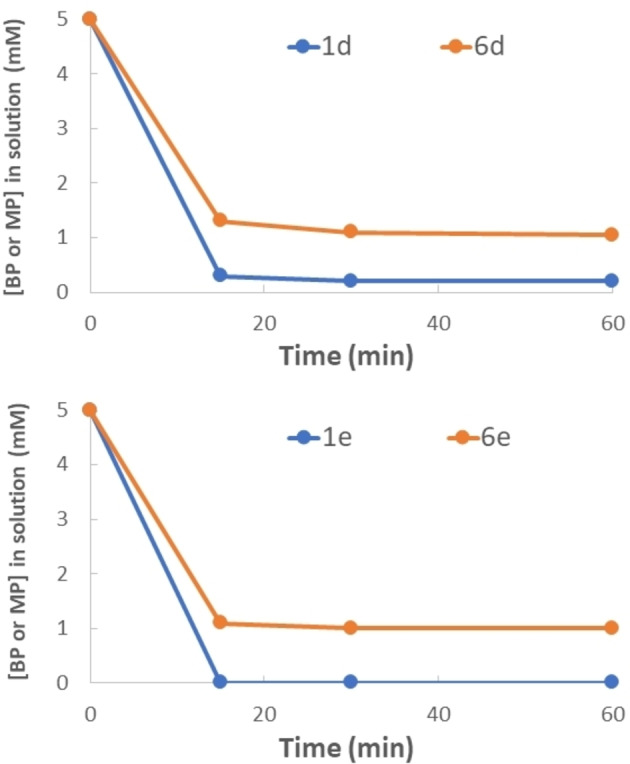
Binding on ZrNPs of **1 d** compared to the monophosphonic derivative **6 d** (top) and **1 e** compared to the monophosphonic derivative **6 e** (bottom). [**1 d**]_0_=[**1 e**]_0_=[**6 d**]_0_=[**6 e**]_0_= 5 mM, ZrNPs 50 mg in 5 mL pH 4 buffer solution.

To compare the affinity of the BPs with respect to commercially available monophosphonates, a series of affinity tests were carried out under identical experimental conditions comparing 2‐aminoethylphosphonic acid (**6 d**) with **1 d** and 3‐phosphonopropionic acid (**6 e**) with **1 e** at pH 4.0 as preferred buffer conditions (Figure 3).

From the two plots of Figure 3 it is evident that both **1 d** and **1 e**, regardless the presence of amino or carboxylic unit in the side chain, ensured high loading on the ZrNPs probably due to the chelating ability of the BP moiety with respect to monophosphonate derivatives **6 d** and **6 e**. In fact, while both BPs led to almost quantitative loading in short time, the two monophosphonates were loaded with 80 % maximum loading efficiency after 60 minutes.

Similarly, to what previously reported for **1 a**, the ZrNPs functionalized with both **1 d** and **6 d** were isolated by centrifugation, washed with pure water, dried and the release over time at pH 8.8 was monitored by NMR observing that in 3 h the monophosphonic acid **6 d** was released for about 11 % while **1 d** only for about 5 %. All these comparative measurements confirmed the higher affinity of BPs with respect to the monophosphonic counterparts, thus providing further advantages to the use of BPs for the tailoring of the surface properties of metal oxide NPs.

#### Analysis of the interaction between 1 a and ZrNPs

Even though it is likely that the phosphonate moieties of **1 a** interact with the Zr metal atoms on the surface of the NPs through coordination and possible chelation on the metal centers, the presence of charges on the surface of the NPs at different pH conditions and the dissociation equilibria of the phosphonic acid units of **1 a** need to be considered to better describe on grafting process.

**Figure 4:**
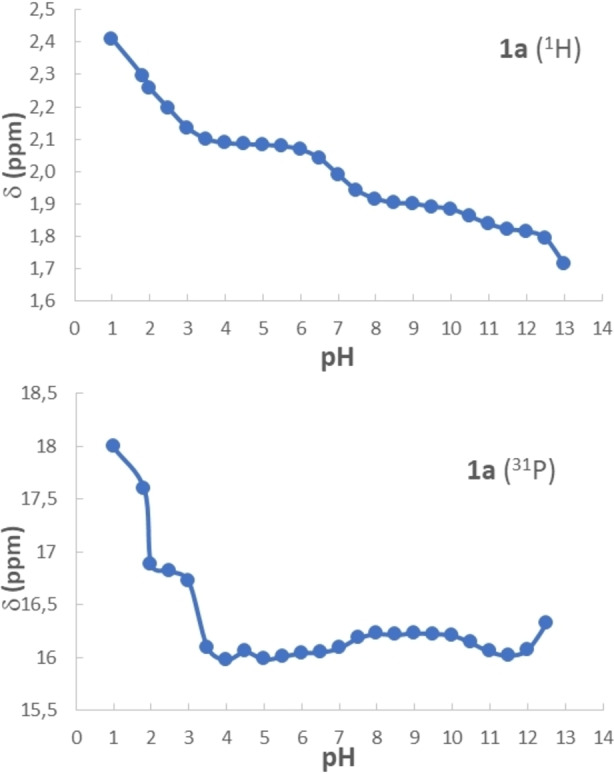
Plot of the ^1^H (top, methylene signal) and ^31^P(^1^H) (bottom) NMR chemical shifts of **1 a** as function of the pH for the determination of the pKa values of the **1 a**.

**Figure 5:**
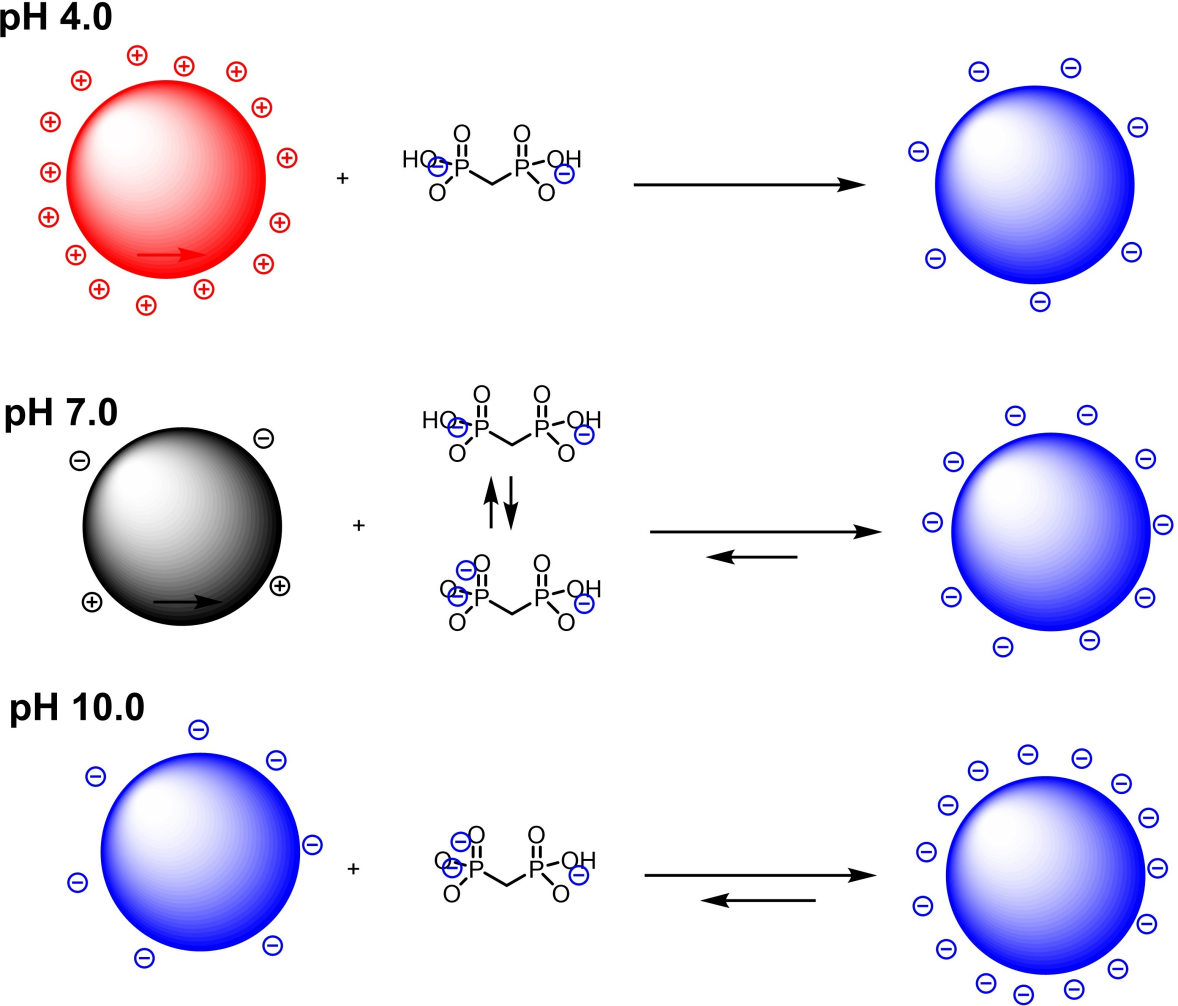
Schematic representation of the charge distribution on the ZrNPs before and after interaction with **1 a**.

The solution speciation of **1 a** as a function of the pH was investigated by means of ^1^H and ^31^P NMR since the chemical shift is known to be greatly affected by the acid‐base equilibria in aqueous solution.^[36]^ Series of samples containing **1 a** (5 mM) at different pH were prepared and the chemical shift values of the ^1^H of the methylene unit and of ^31^P nuclei of the phosphonate moieties were determined and plotted in Figure 4. ^1^H NMR enabled a better determination of pK_3_ and pK_4_, while ^31^P(^1^H) NMR demonstrated to be more sensitive for the determination of pK_1_ and pK_2_. From the obtained chemical shift profiles (see Supporting Information), it was possible to determine the values of pKa_1‐4_ of **1 a** corresponding to 1.9, 3.2, 7.0 and 10.6. With all these information at hand, it was possible to provide better interpretations of the effect of the pH on the loading of **1 a** on ZrNPs. In fact, at pH 4 from ζ‐potential measurements (see later in Figure 7) it is known that ZrNPs are positively charged and, considering the pKa values of **1 a**, the latter species is mainly present as a *bis*‐anion. Therefore, a great electrostatic attraction occurs between **1 a** and the ZrNPs leading to the presence of negative charges on the surface of the functionalized ZrNPs. This is confirmed observing that the more **1 a** binds on the ZrNPs and the more negatively charged the surface of zirconia becomes, as confirmed by ζ‐potential measurements (se later on Figure 7). Moving to pH 7, ZrNPs are almost neutral and **1 a** is basically a mixture of bis‐anion and tris‐anion, therefore the binding occurs still driven also by ionic interaction but with lower efficacy compared to acidic pH where stronger electrostatic attraction occurs. At basic pH like in TRIZMA buffer the ZrNPs are already negatively charged and **1 a** is in the form of a tris‐anion, therefore binding occurs but electrostatic repulsion makes the loading less efficient, as observed in Figure 5. At all pH values the presence of **1 a** on the surface of the ZrNPs ensures the presence of negative charges on the surface thus improving the colloidal stability and preventing aggregation (Figure 5).

#### Binding affinity of 1 b‐1 f with ZrNPs

The study of the interaction between BPs and ZrNPs was extended also to the other BPs **1 b**–**1 f** reported in Scheme 1 and characterized by different substituents on the central methylene atom, ranging from neutral methyl, allyl or functional hydroxyl, amino and carboxylic moieties. All the BPs were tested initially at pH 4.0 with acetate buffer with 5 mL of a 5 mM solution in the presence of 50 mg of ZrNPs. In all cases the uptake of the BPs was complete within 15 min from the addition regardless the different substituent present on the structure of the BPs. This clearly speaks for the peculiarity of the bisphosphonic acid moiety to ensure good interaction with zirconia, especially at pH 4.0 where strong charge interaction occurs.

#### Release experiments of the BPs from the ZrNPs

In order to fully investigate the stability of the ZrNPs‐**1 a**–**f**, the loaded NPs were isolated by centrifugation, washed with water and re‐dispersed in different buffer solutions monitoring the release of the corresponding BPs (Table 2). As long as the functionalization with the model BPs **1 a** is concerned, its release from the ZrNPs was investigated at pH 4.0 without observing any presence of the free BP in solution even after 48 h (Table 2). This indicates that only moving to basic pH conditions some release of the BP is possible. In fact, only at pH 8.8 in TRIZMA buffer the release was 3 % after 1 h, 11 % after 24 h and 19 % after 48 h. Only under drastic basic conditions (100 mM of KOH) the release was about 30–33 % after 30 minutes and remained the same for up to two days. It is in fact known that the surface of zirconia at very high pH values is resorbed and this explains the large release of the BPs. Using PBS as a phosphate buffered saline solution (10 mM NaH_2_PO_4_, 20 mM Na_2_HPO_4_, 150 mM NaCl) the release in solution was 7 % after 1 h and raised only up to 9 % after 48 h.

**Table 2:** Release of BPs determined by ^1^H quantitative NMR at various pH values. PBS: phosphate buffered saline.

BP	Time (h)	Buffer	Release (%)
**1 a**	1	pH 4.0 Acetate	0
	24		0
	48		0
**1 a**	1	pH 8.8 TRIZMA	3
	24		11
	48		19
**1 b**	1	pH 8.8 TRIZMA	4
	24		7
	48		9
**1 c**	1	pH 8.8 TRIZMA	3
	24		8
	48		10
**1 d**	1	pH 8.8 TRIZMA	16
	24		19
	48		23
**1 e**	1	pH 8.8 TRIZMA	0
	24		0
	48		0
**1 e**	1	PBS	2
	24		3
	48		5
**1 f**	1	pH 8.8 TRIZMA	0
	24		0
	48		0

For the other BPs reported in Scheme 1, it is evident that the substituted **1 b**, **1 c** and even more the allyl substituted **1 f** with respect to **1 a** are characterized by a higher degree of hydrophobicity and this turns out into decreased release in solution due to lower solubility in water. It is in fact known that BPs with hydrophobic residues tend to aggregate in solution with a behavior reminiscent of the formation of micelles by surfactants, therefore a contribution of the hydrophobic effect on the binding of these BPs on ZrNPs is likely to occur. It is also clear that for **1 e** the presence of the carboxylic unit provides an extra binding point that transforms the BPs into a tridentate species. In fact, **1 e** is not released in solution even after 48 h and some release is observed only using phosphate buffered saline (PBS) with which it is likely that a displacement effect of the inorganic phosphate on the BP can take place. For the amino derivative **1 d** the latter moiety does not act as an extra binding point for Zr due to its hard character as Lewis acid atoms and conversely, it promotes the solubilization in water and a larger degree of release in solution.

#### Characterization of the functionalized ZrNPs

**Figure 6:**
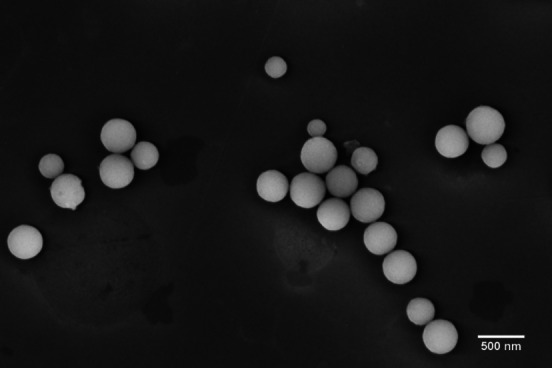
FE‐SEM image of ZrNPs‐**1 a**.

The ZrNPs functionalized with **1 a** were characterized by FE‐SEM, ζ‐potential, FTIR, and XPS analyses. The surface functionalization of ZrNPs did not lead to structural modification with respect to the original naked ZrNPs, as visible from FE‐SEM image reported in Figure 6. Moreover, no aggregation occurred after BP modification observing that the particles did not form clusters and remained well separated.

**Figure 7:**
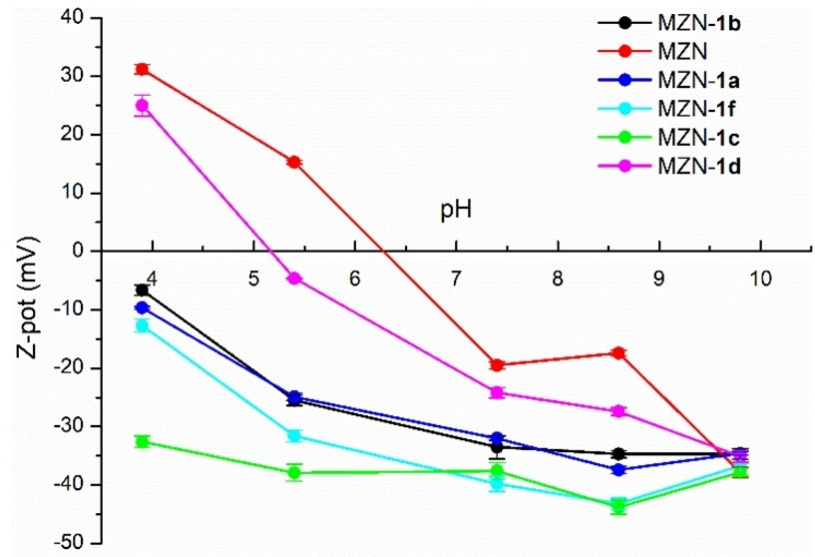
ζ‐potential as a function of the pH of the solution for bare ZrNPs and ZrNPs functionalized with **1 a**‐**1 f**.

A series of ζ‐potential measurements were carried out on the NPs obtained before and after functionalization with **1 a**–**f** in the range of pH between 4.0 and 10. The results reported in Figure 7 clearly shows that the original ZrNPs are characterized by the presence of surface positive charges at acidic pH and that moving to higher pH the surface ζ‐potential of the NPs decreases becoming negatively charged at pH 10, displaying an isoelectric point (IEP) falling in the range between pH 6.5 and 7.0. Surface modified nanoparticles with **1 a**–**f** display a different behaviour. In particular, it is worth to notice that for the ZrNPs‐**1 a** in all the pH range investigated the surface of the NPs turned out to be highly negatively charged due to the presence of several polyanionic **1 a** molecules that modify the surface of the ZrNPs and, thanks to the high charge, prevent aggregation and ensure high colloidal stability for several days.

Similarly, all the other BPs provided comparable effects on the ζ‐potential of the NPs, except for the amino‐BP **1 d**. In the latter case at pH 4.0 the ζ‐potential is highly positive due to the presence of protonated primary amino side chain of the BP that overall overcome the number of negative charges on the surface leading to positively charges NPs.

Moving to higher pH values, the IEP at acidic pH is likely to be due to the terminal primary amino group that above pH 6 becomes neutral and the NPs become more and more negatively charged. It is worth to notice that between pH 6 and 9 all the BPs provide the nanoparticles with a superficial negative charge with overall potential below −20 mV that is highy responsible for the good colloidal stability. Moreover, repeated measurements on the same samples showed good reproducibility of the colloidal solutions, as clearly demonstrated by the rather small error scale‐bar for each ζ‐potential data.

**Figure 8:**
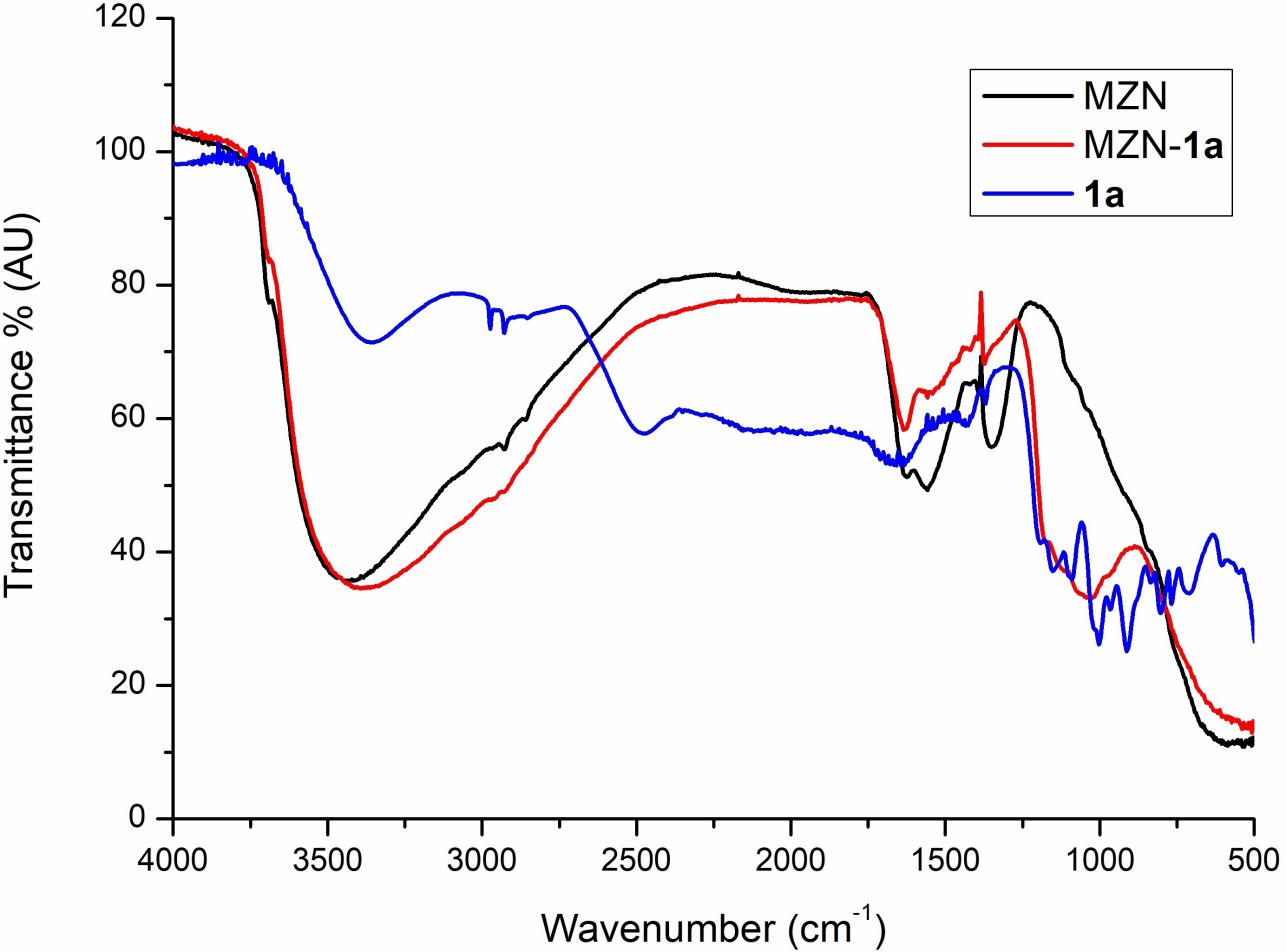
FTIR spectra of pure **1 a** (blue), bare ZrNPs (black) and ZrNPs‐**1 a** (red).

DRIFT‐FTIR spectra of bare ZrNPs and ZrNPs‐**1 a** and ATR‐FTIR spectrum of pure **1 a** are reported in Figure 8. Significant changes in the P−O region (900–1250 cm^−1^) were observed comparing the free **1 a** with the functionalized ZrNPs‐**1 a**. The stretching vibration of the P−OH at 906 cm^−1^ disappears in the infrared spectra of modified NPs, while other peaks typical of the free **1 a** become a single broad band after conjugation. These modifications of the IR spectrum are associated with the bonding of the BPs acid on the surface, probably via condensation reactions.[^37^, ^38^] The broadening and shifting of all peaks corresponding to the P−OH vibrations were observed for all the modified samples with BPs **1 a**–**1 f** (see Supporting Information) and this common behaviour further supports the covalent modification of the ZrNPs. Accordingly, in the case of BPs like **1 b** the presence of other peaks related to specific residues like the bending vibration of C−H bond were observed.

**Figure 9:**
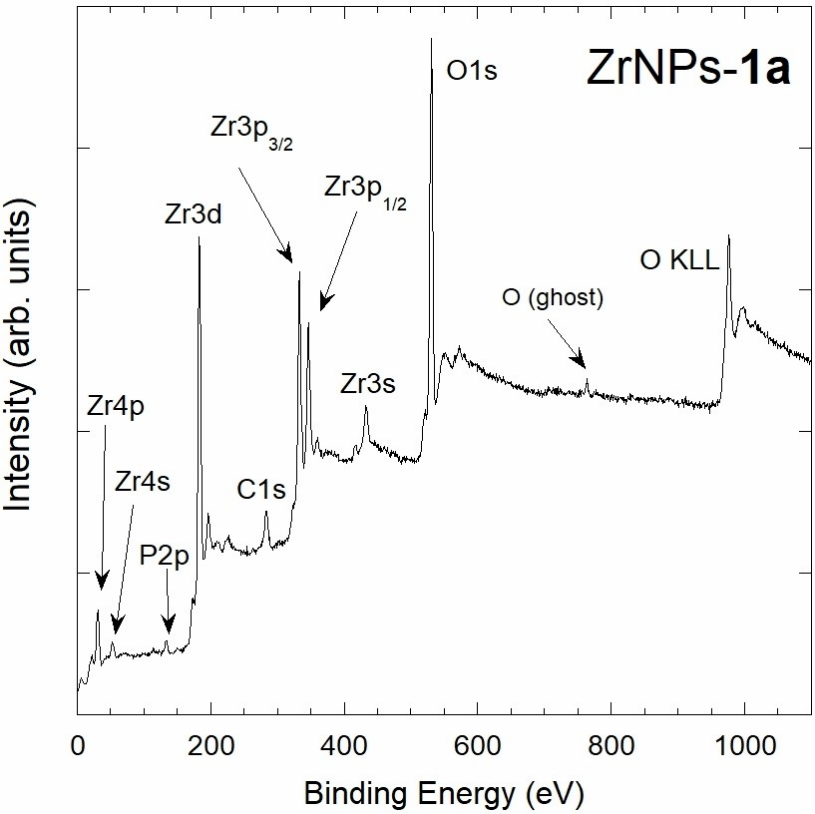
The XPS survey spectrum of ZrNPs‐**1 a** showing a dominant signal attributed to the ZrNPs and weak signals attributed to P and C, related to the presence of **1 a** on the nanoparticles surface.

To further validate the presence of BPs on the surface of the functionalized ZrNPs, XPS spectra were performed on different samples. Being the XPS sampling depth typically around 5–10 nm, this technique evidences the presence on the surface of different chemical elements belonging to the molecules of interest, with a detection limit slightly lower than 1 %. Figure 9 shows a wide‐range survey XPS spectrum for ZrNPs‐**1 a** sample, evidencing the presence of bands related to Zr, O, C, and P. By spectra recorded in higher resolution condition and after correction for the sample charging using a Zr internal reference (see Supporting Information), the binding energy (BE) was determined for Zr3p_3/2_ (332.8 eV), Zr3p_1/2_ (345.8 eV) and O1s (530.2 eV) bands: the detected BE values are characteristic for ZrNPs. P2p and C1s bands were centered at 133.1 eV and 284.3 eV, respectively.

C, O, Zr, P, S, and N atomic fraction for the samples ZrNPs‐**1 b**–**f** are summarized in Table 3 as detected by XPS. For all the functionalized ZrNPs samples the presence of the specific elements, in particular P arising from the bisphosphonate moiety, was confirmed. Moreover, the comparison of the atomic fraction of P, S, and N shows a good agreement with the nominal amount ratio of these elements in the corresponding BPs (Scheme 1). The binding energy values of the P2p, S2p, and N1s bands were determined by detail spectra and the results are reported in Table 4.

**Table 3:** C, O, Zr, P, S, and N atomic fraction as obtained by XPS data for ZrNPs, ZrNPs‐**1 a**, ZrNPs‐**1 b**, ZrNPs‐**1 c**, ZrNPs‐**1 d** and ZrNPs‐**1 f** samples.

Samples	C (%)	O (%)	Zr (%)	P (%)	S (%)	N (%)
ZrNPs	27	54	19	–	–	–
ZrNPs‐**1 a**	17	62	19	∼2.8	–	–
ZrNPs‐**1 b**	17	61	19	∼2.4	–	–
ZrNPs‐**1 c**	16	61	16	∼4.0	∼1.7	–
ZrNPs‐**1 d**	15	60	19	∼3.4	∼1.3	∼1.3
ZrNPs‐**1 f**	18	60	18	∼2.5	∼1.0	–

**Table 4:** Binding energy values of P2p, S2p, and N1s bands as detected in the functionalized ZrNPs.

#	P2p (eV)	S2p (eV)	N1 s (eV)
ZrNPs‐**1 a**	133.4	–	–
ZrNPs‐**1 b**	133.5	–	–
ZrNPs‐**1 c**	133.2	163.4	–
ZrNPs‐**1 d**	133.2	163.6	400.7
ZrNPs‐**1 f**	133.3	163.4	–

**Figure 10:**
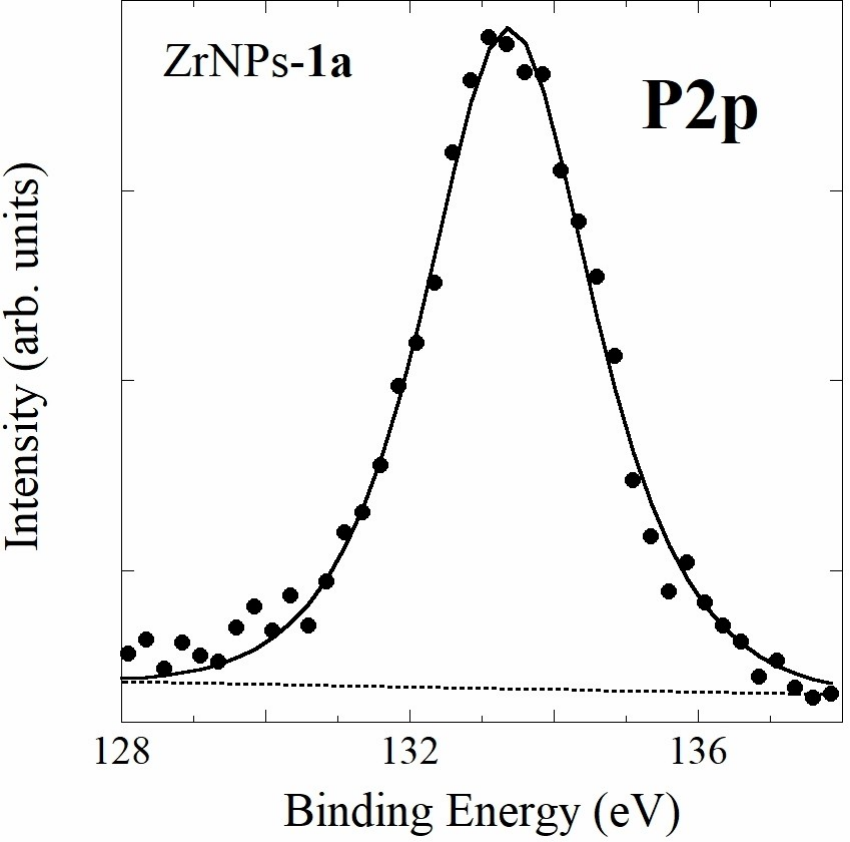
XPS detail spectrum of P2p band, recorded for ZrNPs‐**1 a** sample.

The detected values are compatible with the chemical environment of the elements considered in the different functional groups. In particular, the P2p BE values detected for all the P‐containing samples (falling in the range 133.2–133.5 eV) are slightly lower than those reported for the single BP molecule.^[39]^ This is ascribable to the coordination of the phosphonate moieties on the superficial Zr atoms as observed also for similar systems.^[40]^ These experimental findings once more attest the desired functionalization of the ZrNPs. In Figure 10 the detail XPS spectrum of P2p band for ZrNPs‐**1 a** sample is reported. As far as the S2p and N1s bands are concerned, their BE values are consistent with the presence of S atoms in the thioether side chains of the BPs.^[41]^ and with N atoms in amine functionalized ZrNPs with **1 d**,^[42]^ respectively.

#### Solid State NMR experiments on functionalized ZrNPs

**Figure 11:**
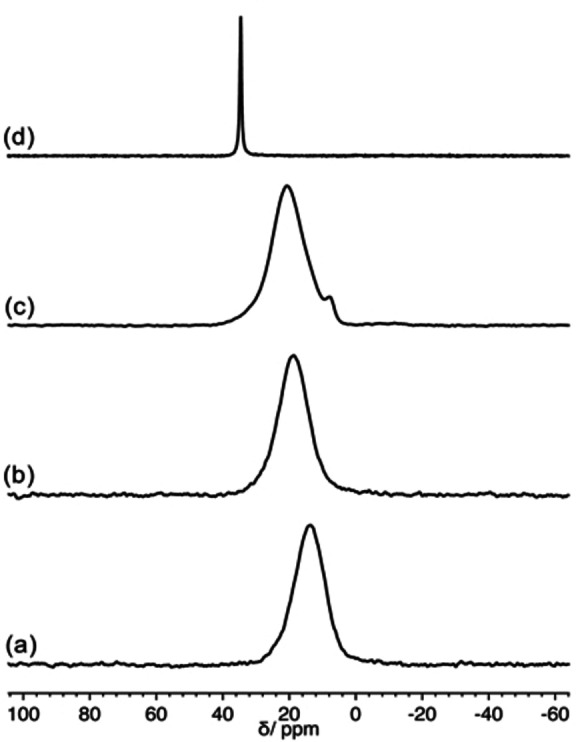
^31^P DE‐MAS spectra of: (a) ZrNPs‐**1 a** (b) ZrNPs‐**1 b** (c) ZrNPs‐**6 e** (d) pristine **6 e**.

The functionalization of ZrNPs with **1 a** and **1 b** was also investigated by means of Solid State NMR spectroscopy, in particular ^31^P‐MAS spectra provide useful information on the local environment of ^31^P nuclei. The spectra of both ZrNPs‐**1 a** and ZrNPs‐**1 b** (Figure 11) show a single broad peak centered at about 14 and 19 ppm, respectively.

These peaks are shifted by about −10 ppm with respect to the signals of the corresponding pure bisphosphonic acids.^[43]^ Based on the literature, where the interaction between mono‐ and, only in few cases, BPs and zirconia has been investigated exploiting ^31^P Solid State NMR spectroscopy,[^44^, ^45^] a≈10 ppm decrease in the ^31^P chemical shift can be attributed to the formation of a multi‐dentate anchoring of **1 a** and **1 b** to the NP surface by reaction of P−OH to form P−O−Zr bonds. The broadness of the peaks is indicative of the presence of a distribution of geometries around ^31^P nuclei and, on the whole, of the amorphous nature of the organic layer. Additional information arises from ^1^H‐MAS spectra (see Supporting Information): for both ZrNPs‐**1 a** and ZrNPs‐**1 b** no signals ascribable to P−OH hydrogen nuclei, expected at about 10–11 ppm[^42^, ^46^] were observed, thus suggesting that most of them reacted with zirconia.

Moreover,^1^H‐^31^P HETCOR experiments, in which spatially close ^1^H and ^31^P nuclei give rise to cross‐peaks, highlighted for both ZrNPs‐**1 a** and ZrNPs‐**1 b** an expected strong correlation between phosphorus nuclei of **1 a** and **1 b** and ^1^H nuclei of directly bonded ‐CH_2_‐ groups (at about 2 ppm), and a weaker correlation with protons resonating at about 6 ppm (see Supporting Information). These protons give rise to a broad signal in the corresponding ^1^H‐MAS spectra, which can be ascribed to water firmly adsorbed on ZrNPs and/or possible residual Zr‐OH groups,^[47]^ both strongly hydrogen‐bonded. It is possible that these protons establish hydrogen‐bonds also with P=O oxygens of phosphonates. Moreover, in one case,^[48]^ also P−OH protons in functionalized mesoporous silica have been reported to resonate at 6.5 ppm, thus it cannot be ruled out that also some residual P−OH groups contribute to this signal.

For comparison we also looked at ZrNPs functionalized with 3‐phosphonopropionic acid **6 e**. In Figure 11 the ^31^P‐MAS spectra of ZrNPs‐**6 e** and of pristine **6 e** are reported. While pristine **6 e** shows a single narrow peak resonating at about 35 ppm, characteristic of a crystalline solid, ZrNPs‐**6 e** gives rise to a very broad signal centered at a chemical shift of 20 ppm, with a minor component at about 8 ppm.

Similarly to BPs, the 15 ppm decrease of the chemical shift in passing from the pure acid to the zirconia hybrid can be ascribed to a bi‐dentate anchoring of **6 e** on the zirconia surface, involving the reaction of most P−OH groups. The small signal at 8 ppm can be tentatively ascribed to a minor fraction of molecules forming a tri‐dentate anchoring also involving the P=O group or to a small amount of bulk metal phosphonate.^[42]^ ^1^H‐MAS spectra (Supporting Information) confirmed these results: in the spectrum of ZrNPs‐**6 e** a signal at about 11 ppm, due to P−OH, observed in the spectrum of pristine **6 e**, is not detected, suggesting that most of P−OH groups reacted. Moreover, while in the ^1^H‐^31^P HETCOR spectrum of pristine **6 e** (Supporting Information) cross‐peaks were observed between phosphorus nuclei and methylene (at about 2 ppm) and P−OH (at about 11 ppm) hydrogen nuclei, the HETCOR spectrum of ZrNPs‐**6 e** appeared completely similar to that of ZrNPs‐**1 a** and ZrNPs‐**1 b**.

## Conclusions

In conclusion we successfully developed a straightforward method for amorphous mesoporous ZrNPs surface modification based on the interaction with aqueous solutions of bisphosphonic acids. The latter class of organic molecules can be easily prepared bearing different substituents in the side chain that can be tailored in terms of length of C atoms, presence of functional groups and polarity. The use of the BPs enabled the functionalization of the ZrNPs leading to new highly charged materials characterized by very low aggregation properties and high colloidal stability. The interaction between BPs and ZrNPs has been thoroughly investigated by quantitative ^1^H NMR in solution to ascertain the loading and release properties of the different BPs as function of the pH, SEM, ζ‐potential measurements, IR, MAS NMR and XPS were carried out to investigate the surface properties of the NPs before and after functionalization. Overall, BPs ensure higher affinity compared to commercially available monophosphonates thanks to both better chelating properties and higher charge content. BPs turned out to be suitable for the modification of the surface properties of ZrNPs with a straightforward method alternative to the use of trichloro or *tris*‐alkoxysilanes commonly applied for silica NPs.^[49]^ The possibility to use BPs bearing various functional groups in the side chain like amino, carboxylic acid and alkene paves the way for further covalent functionalization, in particular for biomedical applications. A possible extension of this grafting approach is advisable also for many other kind of metal oxides in nanoparticle forms.^[50]^

## Conflict of interest

The authors declare no conflict of interest.

## Supporting information

As a service to our authors and readers, this journal provides supporting information supplied by the authors. Such materials are peer reviewed and may be re‐organized for online delivery, but are not copy‐edited or typeset. Technical support issues arising from supporting information (other than missing files) should be addressed to the authors.

Supporting Information
