# Supplementary material for: Modification of Amorphous Mesoporous Zirconia Nanoparticles with Bisphosphonic Acids: A Straightforward Approach for Tailoring the Surface Properties of the Nanoparticles
Source: Chemistry. 2021 Nov 17;27(71):17941–51. doi: 10.1002/chem.202103354 (PMC9299609; doi:10.1002/chem.202103354)
Supplement: Supplementary file 1 — Supporting Information [file CHEM-27-17941-s001.pdf]

Chemistry–A European Journal

Supporting Information

Modification of Amorphous Mesoporous Zirconia Nanoparticles with Bisphosphonic Acids: A Straightforward Approach for Tailoring the Surface Properties of the Nanoparticles

Khohinur Hossain, Luca Florean, Anna Del Tedesco, Elti Cattaruzza, Marco Geppi, Silvia Borsacchi, Patrizia Canton, Alvise Benedetti, Pietro Riello, and Alessandro Scarso*

Index

1. General Methods S2
2. Materials S2
3. ZrNPs Characterization methods S2
4. Synthesis of ZrNPs S3
5. Experimental procedures for the synthesis of BP esters **2, 3, 4b-f** S4
6. Experimental procedures for the synthesis of BP acids **1a-f** S36
7. Functionalization of ZrNPs with BP acids S53
8. Quantitative NMR determination of Buffers and BPs interaction with ZrNPs S54
9. FTIR spectra of BP acids and functionalized ZrNPs S64
10. Determination of the grafting density by elemental analyses S65
11. ^{1}H-MAS and ^{1}H-^{31}P HETCOR Solid State NMR spectra of BP functionalized ZrNPs S66
12. Calculation of loading by ^{1}H NMR and grafting density by Elemental analyses S68
13. References S69

1. General Methods

The reactions were followed with TLC Polygram® Sil G/UV_{254}, 0.25 mm thickness. ^1H NMR, ^{13}C NMR, ^{31}P NMR and 2D spectra of soluble organic compounds were recorded with a Bruker Avance 300 and Ascend 400 spectrometers, working at 300-400 MHz for ^1H, 75-100 MHz for ^{13}C and 121-161 MHz for ^{31}P, respectively. Resonance frequencies are referred to tetramethylsilane and 85% H_3PO_4.

Reagents and solvents with high purity degree purchased by the providers were used as given. Otherwise, they were purified following the procedures reported in literature.[S1] Anhydrous solvents were prepared by adding activated 3 Å molecular sieves to the solvent under inert atmosphere. Molecular sieves were activated shortly before the use by continuous heating under vacuum. Flash chromatography were performed with silica gel Merck 60, 230-400 mesh, following procedures reported in the literature.[S2]

Quantitative ^1H NMR determinations

^1H NMR spectra for quantitative determinations were recorded with a Bruker Ascend 400 spectrometer operating at 400 MHz with saturation of the solvent peak of water at 4.79 ppm. Analyses were carried out preparing a standard solution of the BP 5 mM in D_2O buffered solutions 25 mM. Since the release tests were directly carried out in deuterated water, aliquots of 1 mL on the samples were withdrawn at fixed times (0, 30, 60, 90, 120, 240, 420, and 1440 minutes) removing the ZrNPs by centrifugation. The obtained solutions were directly analysed by quantitative NMR acquiring the ^1H NMR spectra of the samples and of the references with the same acquisition parameters such as number of scans 16 and d1 of 10 s on 0.6 mL of sample. The ^1H NMR spectra were analysed with the quantitative Eretic Bruker program considering volume of the solution, the concentration of the mother solution as reference and the number of H atoms assigned to a certain resonance in the spectrum.

2. Materials

Zirconium propoxide (ZP, Aldrich 70%wt in propanol), Hexadecylamine (HDA, Alfa Aesar 90%), Sodium fluoride (NaF, Eurobio 99%), Ethanol (EtOH, Aldrich 99.8%), and Milli-Q water, 2-Aminoethanthiol (Tokyo Chemical Industry, TCI, >95%), 3-mercapto-1-propanol (Tokyo Chemical Industry, TCI, >97%),), Allyl mercaptan (Tokyo Chemical Industry, TCI, >80%), Methyl mercaptan sodium salt (Tokyo Chemical Industry, TCI), , Sodium borohydride (Sigma-Aldrich, ≥ 98%), Bromo trimethylsilane (Sigma-Aldrich, 97%), Trifluoroacetic acid (Sigma-Aldrich, 99%), Benzyl bromide (Sigma-Aldrich, 98%), Bismuth(III) nitrate pentahydrate ($Bi(NO_3)_3 \cdot 5H_2O$, Aldrich 99.9%), poly(vinylpyrrolidone) (PVP40, MW=40 kg/mol, Aldrich), ethylene glycol (EG, Aldrich 99.8%), Urea ($CO(NH_2)_2$, Fluka >99%), $FeCl_2 \cdot 4H_2O$ (Sigma-Aldrich, 99%), $FeCl_3 \cdot 6H_2O$ (Sigma-Aldrich, 98%)

3. ZrNPs Characterization methods

The nitrogen adsorption−desorption isotherms were obtained at the liquid nitrogen temperature (77 K) using a Micromeritics ASAP 2010 volumetric adsorption analyzer. From the adsorption data, the Brunauer−Emmett−Teller (BET) equation was used to calculate the specific surface area, while from the adsorption branches of the isotherms, the Barrett−Joyner−Halenda (BJH) model was used to estimate the pore size distribution and the pore volume was determined from the isotherm using the total adsorption value at relative pressure $p/p_0 = 0.26$.

The dimensions and the morphologies of the nanoparticles were investigated by electron microscopy using a Zeiss Sigma VP Field Emission Scanning Electron Microscope (FE-SEM). Mean and standard deviation of the FE-SEM micrographs were determined measuring at least 50 nanoparticles for each sample. The diameters were measured using the image analysis software ImageJ.

The surface of the functionalized mesoporous zirconia nanoparticles samples was characterized using several supplementing analysis methods. Fourier-transform infrared (FTIR) spectrometric

measurements were carried out with a Spectrum BX (PerkinElmer Co.) spectrometer in both transmission mode and with horizontal ATR accessory equipped with a diamond crystal. The measurements were taken between 4000 and 500 cm^{-1}, averaging 32 scans.

Elemental analyses were performed on a Vario MICRO CHNS instrument.

X-ray photoelectron spectroscopy (XPS) was performed using a Perkin Elmer Φ 5600ci spectrometer using nonmonochromatic Al K_α radiation (1486.6 eV) in the 10^{-7} Pa pressure range. All the binding energy (BE) values are referred to the Fermi level. The correct calibration of the BE scale was verified during analysis by checking the position of both Au $4f_{7/2}$ and $Cu2p_{3/2}$ bands (from pure metal targets), falling at 84.0 and 932.6 eV, respectively [S3]. The raw XPS spectra were fitted using a non-linear least-square fitting program adopting a Shirley-type background and Gaussian–Lorentzian peak shapes for all the peaks (XPSPEAK41 software). Because of surface charging, samples presented a shift of the bands toward higher BEs: the charging effect was corrected by using an internal reference, depending on the considered nanoparticle system ($Zr3d_{5/2}$ band centred at 182.4 eV for ZrO_2 system [S3], $Ti2p_{3/2}$ band at 458.6 eV for TiO_2 system [S3], $Fe2p_{3/2}$ band for 710.9 eV for Fe_2O_3 system [S3,S4]). The uncertainty of all the determined BEs was around 0.2 eV. The atomic composition was evaluated using sensitivity factors as provided by Θ V5.4A software. The relative uncertainty of the calculated atomic fraction of the different elements is around 5−10%.

The ζ-potential of the ZrNPs nanoparticles and different bisphosphonic acid modified ZRNPS nanoparticles were calculated from electrophoretic mobility measurements done with a Zeta sizer Nano ZS (Malvern Instruments Ltd.). The measurements were performed after dilution of colloidal solutions with ultra-pure water and buffer solution (pH range 4 to 10) to 1 wt%. About 2 mL of colloidal solutions were transferred into measuring cell. The measurements were run at E-field= 5.00 V/cm, T= 25 °C, with switch time at t = 33 s and averaging the obtained mobility data over six consecutive scans.

Solid State NMR spectra were recorded on a Varian Infinity Plus 400 spectrometer working at Larmor frequencies of 400.34 and 162.06 MHz for ^1H and ^{31}P nuclei, respectively, using a CP-MAS probe-head accommodating rotors with outer diameters of 3.2 mm. Both ^{31}P and ^1H spectra were recorded under Magic Angle Spinning (MAS) at a spinning frequency of 20 kHz, using a Direct Excitation (DE) pulse sequence, with high-power decoupling from ^1H nuclei in the case of ^{31}P spectra. The duration of the excitation pulse was 2 μs for both ^{31}P and ^1H nuclei. ^{31}P-MAS spectra were recorded with a relaxation delay of 5 s between consecutive transients and about 1000 scans were accumulated for each sample. The signal of H_3PO_4 (80%) at 0 ppm was used as reference for ^{31}P chemical shift scale. ^1H-MAS spectra were recorded accumulating about 200 transients, with a recycle delay of 5 s, for each sample. ^1H-^{31}P HETCOR spectra were recorded at a MAS frequency of 20 kHz, with frequency-switched Lee–Goldburg ^1H homonuclear decoupling, using a contact time of 300 μs in order to minimize ^1H spin-diffusion.

4. Synthesis of ZrNPs

The synthesis of ZrNPs was adapted from the procedure reported in the literature.[S5]
Briefly, the surfactant was dissolved in ethanol, then the correct quantity of sodium fluoride aqueous solution was added. The precursor mixed with ethanol was added dropwise into the primary solution under vigorous stirring. It has been found that the preferred molar ratio of the reagents EtOH:H_2O:HAD (hexadecylamine):ZP:NaF is 750:20:2:1:0.02. The precipitate was recovered by centrifugation, and three washing cycles whit fresh ethanol were performed. The dried powder was put into a Teflon bomb with 6.25 ml of water and 12.5 ml of EtOH per gram of powder and heated at 170 °C for 20 h. To extract the surfactant without structural modifications, the recovered powder was treated with a heat vacuum extraction process at 120°C.

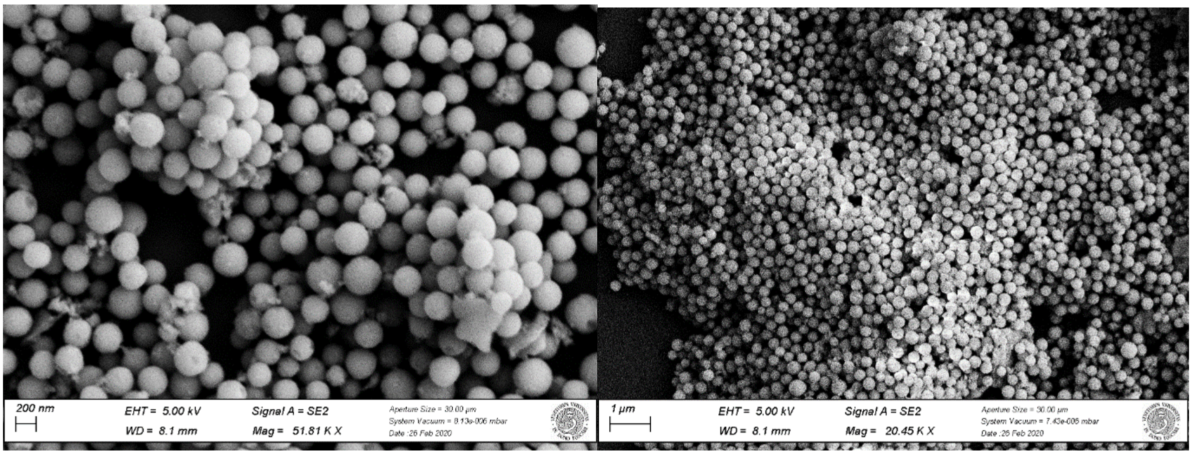

Figure 1. SEM images of synthesized ZrNPs with surface area of 187.4±0.6 m^2/g.

5. Experimental procedures for the synthesis of BP esters 2, 3, 4b-f

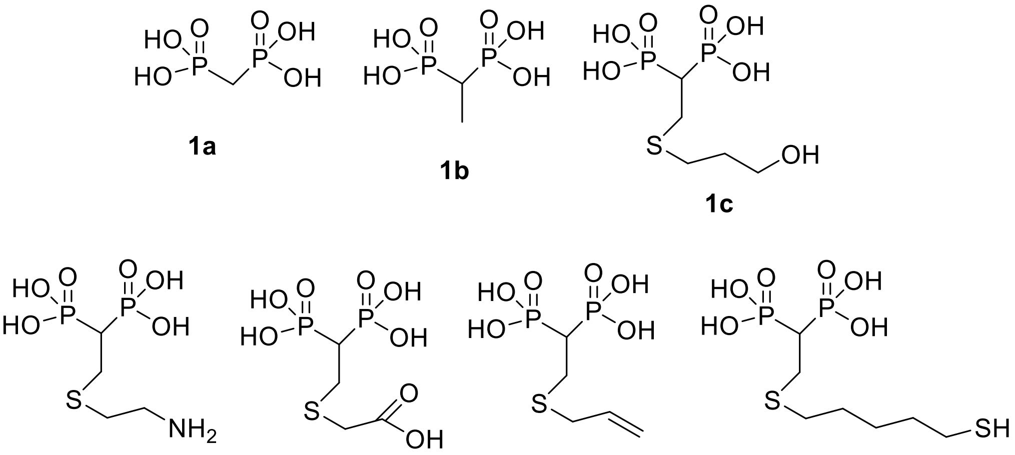

1a 1b 1c 1d 1e 1f 1g

Figure 2. Molecular structure of different BP acids used for the modification of ZrNPs.

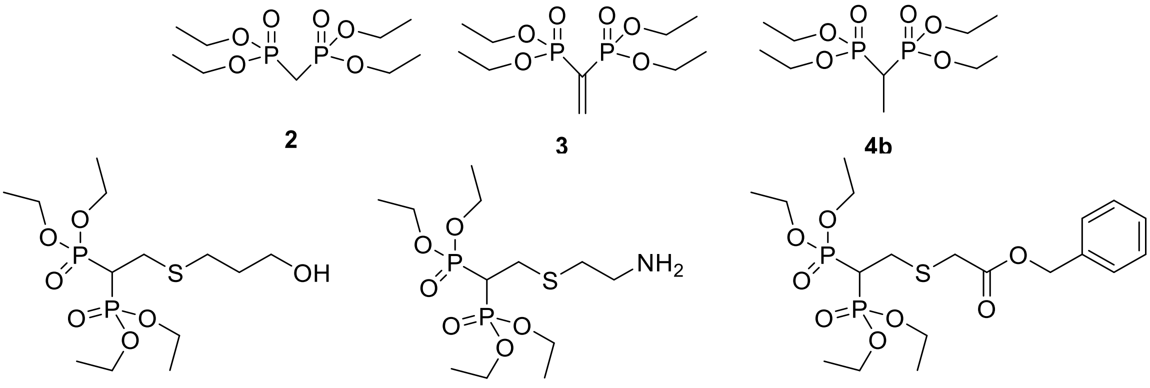

2 3 4b 4c 4d 4e

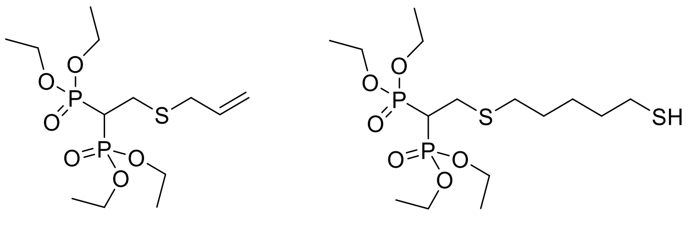

4f **4g**

Figure 3. Molecular structure of different BP acid precursors.

Tetraethyl methylenebis(phosphonate) 2

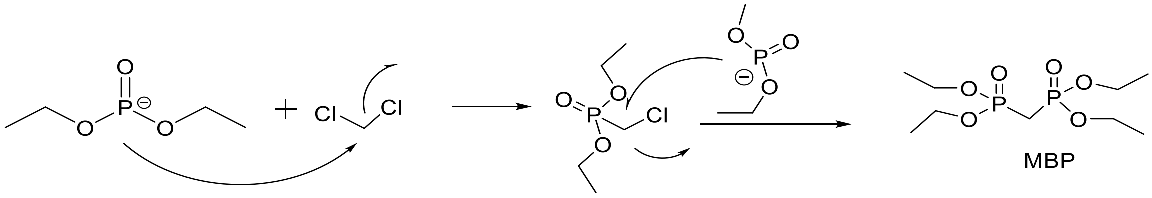

The starting material was synthesised according to literature procedure.[3] In a 500 mL round bottomed flask small pieces of sodium metal (5 g, 220 mmol) were dissolved in 130 mL of ethanol under vigorous stirring. To this, diethyl phosphite (28 mL, 220 mmol) was added dropwise into the clear sodium ethoxide solution and the mixture was left under stirring for 1h at room temperature. The resulting solution was concentrated under rotavapor, and then anhydrous methylene chloride (10 mL, 156 mmol) was added. The solution was left under stirring for 15 days at room temperature. Subsequently, the solution was washed with saturated brine (3 X 50 mL). The organic phase was diluted with CH_2Cl_2 (30 mL) and dried with $NaSO_4$ and the precipitate was filtered. Methylene dichloride was removed from mother liquors with rotavapor. The residue was distilled under high vacuum through Kugelrohr (0.1 mm Hg) to remove impurities. The product remained as colorless oil with satisfying purity (54% yield).

^{1}H NMR (400 MHz, Chloroform-*d*) δ 4.16 – 3.97 (set of m, 8H), 2.34 (t, *J* = 21.0 Hz, 2H), 1.24 (t, *J* = 7.1 Hz, 12H).

^{31}P NMR (162 MHz, Chloroform-*d*) δ 19.33 (s,2p).

^{13}C NMR (101 MHz, Chloroform-*d*) δ 62.62 (t, *J* = 3.0 Hz), 25.43 (t, *J* = 136.9 Hz), 16.36 (d, *J*=3.2 Hz) ppm.

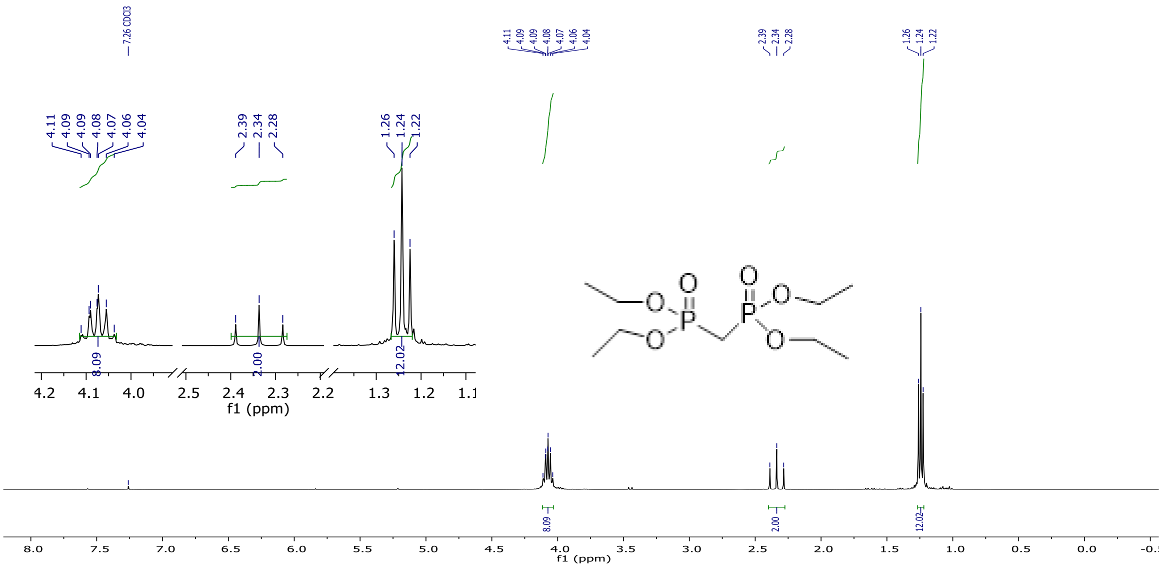

Figure 4. ^{1}H-NMR of **2.**

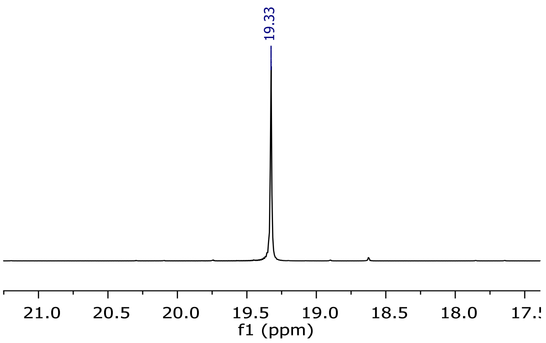

Figure 5. ^{31}P {^{1}H}-NMR of **2.**

Tetraethyl ethene-1,1-diylbis(phosphonate) 3

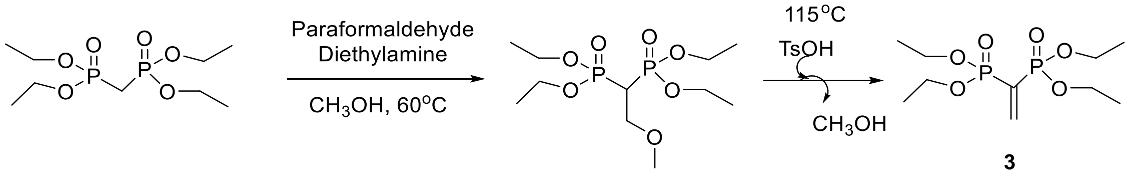

3

In a 500 mL round bottomed flask, paraformaldehyde (3.18 g, 106 mmol) and diethylamine (2.2 mL, 21mmol) were dispersed in 60 mL of methanol and the flask was topped with a reflux condenser. The apparatus was purged with nitrogen and the suspension refluxed until clearness. The resulting solution was cooled to room temperature. Then tetraethyl (methylene) bisphosphonate (5 ml, 20 mmol) was added dropwise into the cooled solution and then reflux for overnight. The completeness of the first step of the reaction was verified by ^{1}H and ^{31}P NMR analysis. Then the solution was concentrated under rotavapor, and the crude was diluted with methanol (30 mL) and then concentrated. The concentrated solution was diluted with toluene and concentrated again. Then the solution was dissolved in anhydrous toluene and p-toluene sulfonic acid (15 mg, 0.09 mmol) was added to the solution. A pressure-equalizing dropping funnel filled with activated 3 Å molecular sieves was placed over the flask and topped with a reflux condenser. The apparatus was purged with nitrogen and the suspension refluxed for 24 hours. The completeness of the reaction was verified by ^{1}H and ^{31}P NMR analysis. The resulting solution was cooled to room temperature and concentrated under rotavapor. Then the residue was dissolved in CH_2Cl_2, washed twice with H_2O, dried with Na_2SO_4, and finally CH_2Cl_2 was removed from mother liquor with rotavapor. The product was obtained as light-yellow oil (5.3 g, 17.6 mmol, 90% yield).

^{1}H NMR (400 MHz, Chloroform-*d*) δ 6.88 (dd, *J* = 71.6, 9.3 Hz, 2H), 4.17 – 3.89 (set of m, 8H), 1.24 (t, *J* = 7.1 Hz, 12H).

^{31}P{^{1}H} NMR (162 MHz, Chloroform-*d*) δ 12.93 (s,2P).

^{13}C NMR (101 MHz, Chloroform-*d*) δ 149.29 (s), 132.17 (t, *J* = 166.6 Hz), 62.78 (t, *J* = 2.8 Hz), 16.38 (t, *J* = 3.3 Hz) ppm.

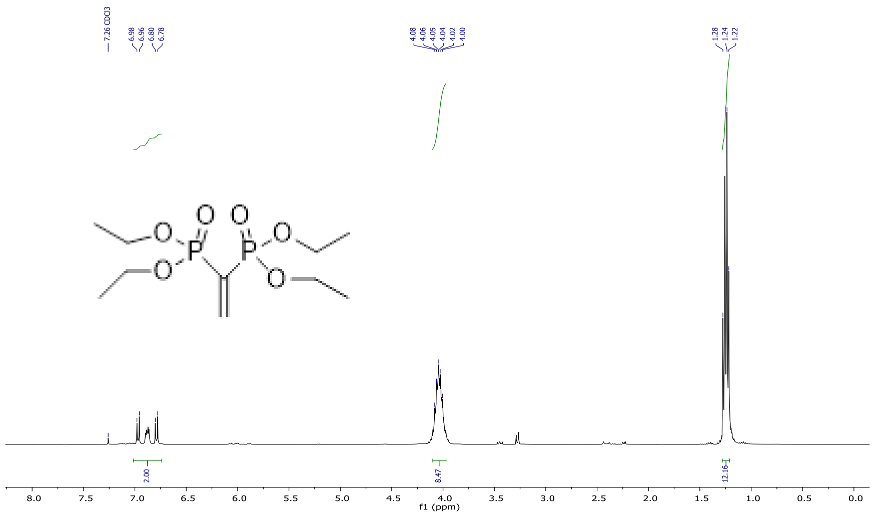

Figure 6. ^{1}H-NMR of **3.**

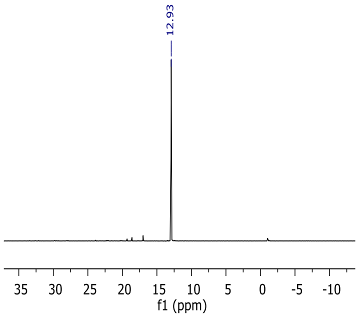

Figure 7. ^{31}P {^{1}H}-NMR of **3.**

Tetraethyl ethane-1,1-diylbis(phosphonate) 4b

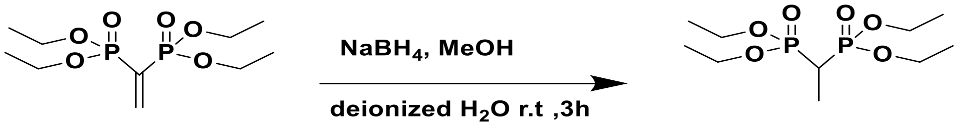

In a 100 mL round bottom flask equipped with magnetic stirring bar, vinylidene bisphosphonate tetraethyl ester (VBP, 500 mg, 1.67 mmol) was added in 5 mL methanol. Then $NaBH_4$ (63 mg, 1.67 mmol) was added to the solution and left under stirring for 3h at room temperature under inert atmosphere. Then 1 mL deionized H_2O was added continuing stirring for 30 min. After that, methanol was removed with rotavapor. The crude was dissolved in 10 mL ethyl acetate and then washed with saturated brine (2X 50 mL), dried with Na_2SO_4 and filtered. Finally, ethyl acetate solvent was removed with rotavapor. The product was obtained as colorless liquid (450 mg, 1.49 mmol, 95% yield).

^{1}H NMR (400 MHz, Chloroform-*d*) δ 4.18 – 4.02 (set of m, 8H), 2.44 – 2.26 (set of m, 1H), 1.40 (td, *J* = 17.2, 7.4 Hz, 3H), 1.28 (t, *J* = 7.1 Hz, 12 H).

^{31}P{^{1}H}- NMR (162 MHz, $CDCl_3$) δ 24.04 (s, 2P). ^{1}H {^{31}P}-NMR (400 MHz, Chloroform-*d*) δ 4.16 – 4.06 (set of m, 8H), 2.33 (q, *J* = 7.4 Hz, 1H), 1.40 (d, *J* = 7.4 Hz, 3H), 1.28 (t, *J* = 7.1 Hz, 12H).

^{13}C NMR (101 MHz, Chloroform-*d*) δ 62.53 (d, *J* = 6.7 Hz), 31.06 (t, *J* = 136.2 Hz), 16.39 (d, *J* = 1.7 Hz), 16.32 (d, *J* = 1.7 Hz), 10.19 (t, *J* = 6.2 Hz).

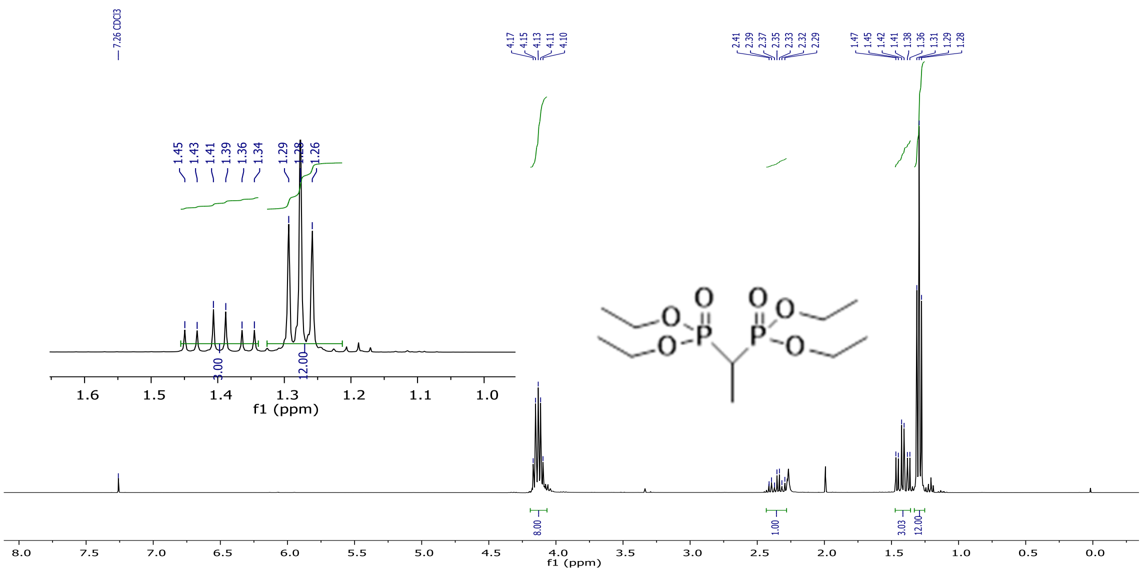

Figure 8. ^{1}H-NMR of EBP ester **4b**

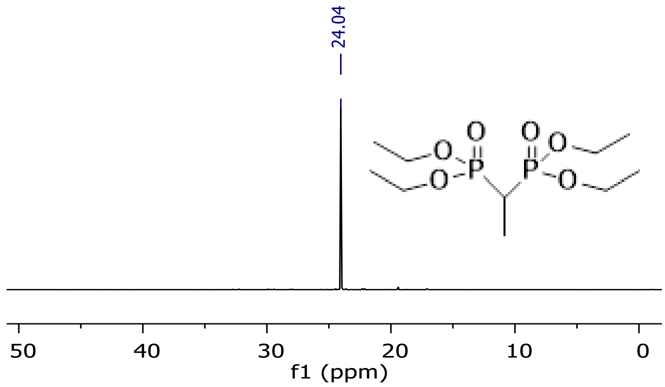

Figure 9. ^{31}P {^{1}H}-NMR of EBP ester **4b**

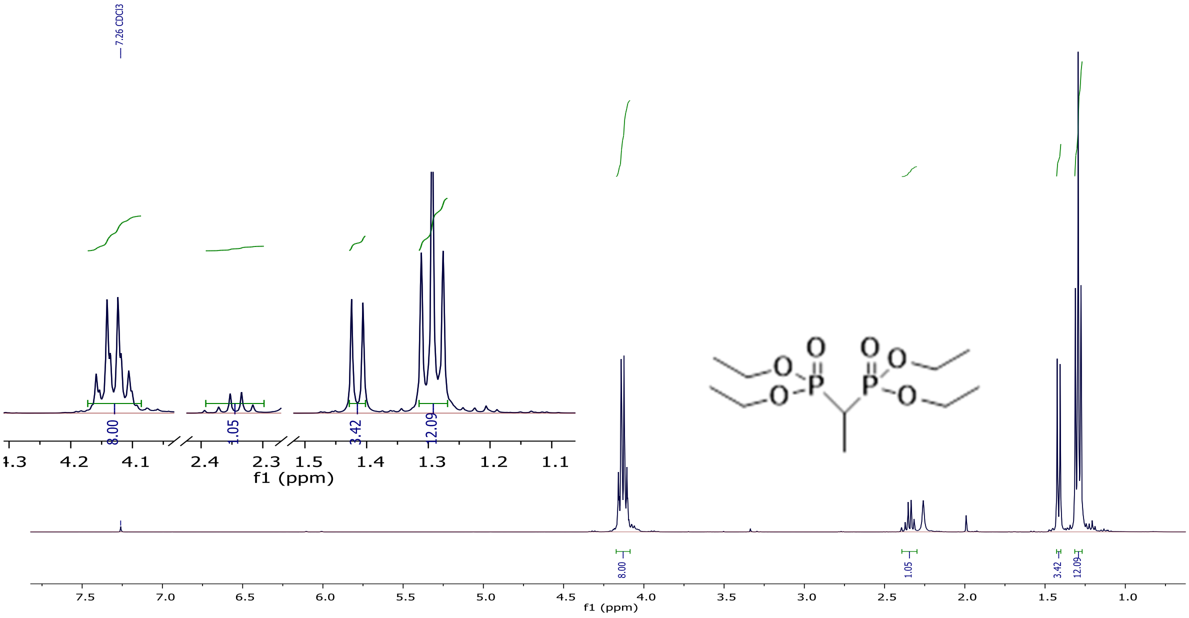

Figure 10. ^{1}H {^{31}P}-NMR of **4b.**

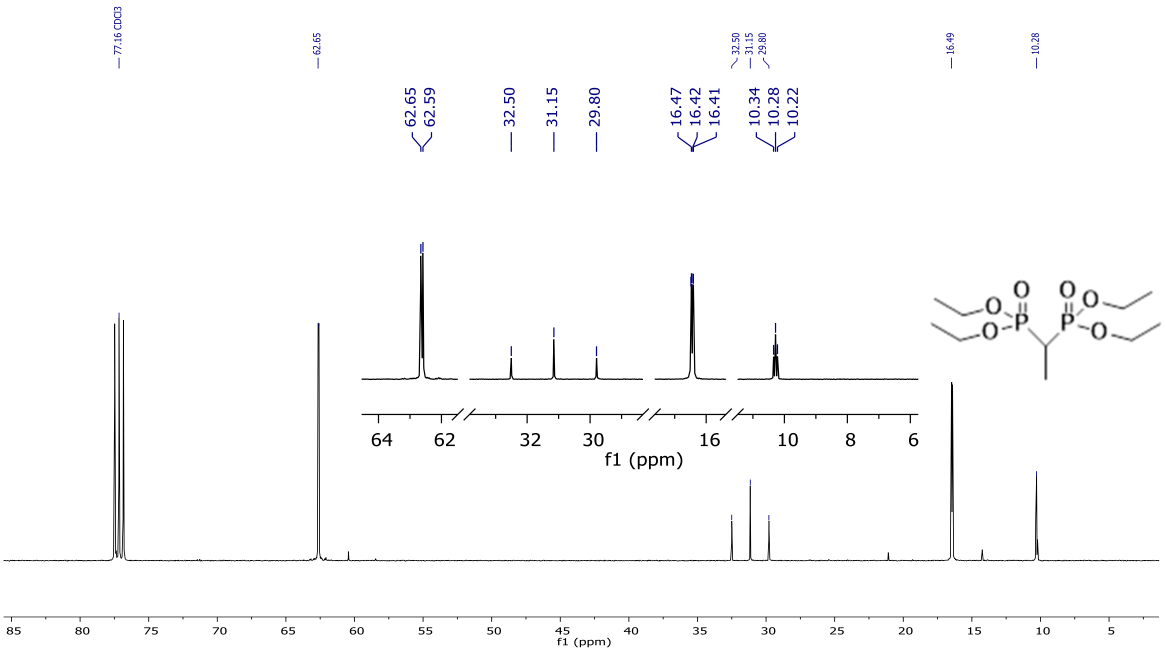

Figure 11. ^{13}C-NMR of **4b.**

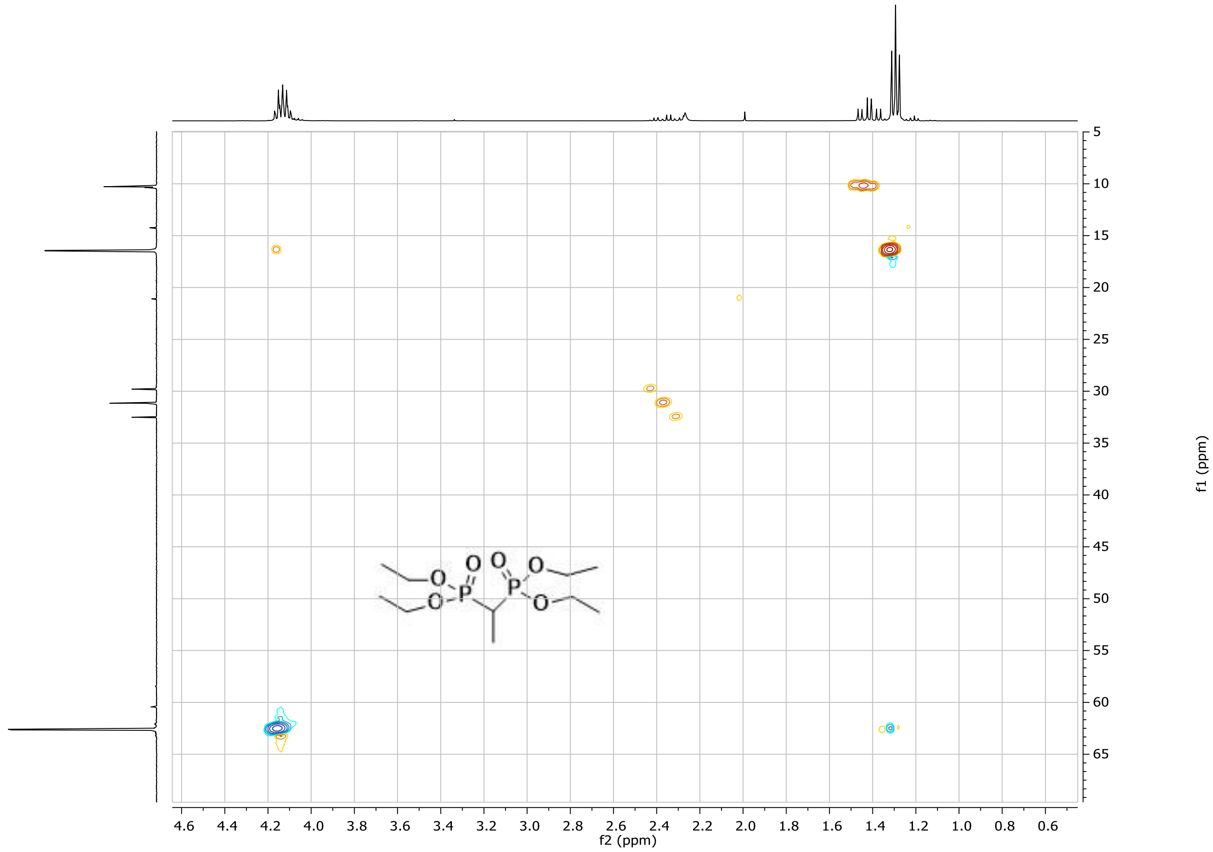

Figure 12. 2D-NMR HSQC of **4b.**

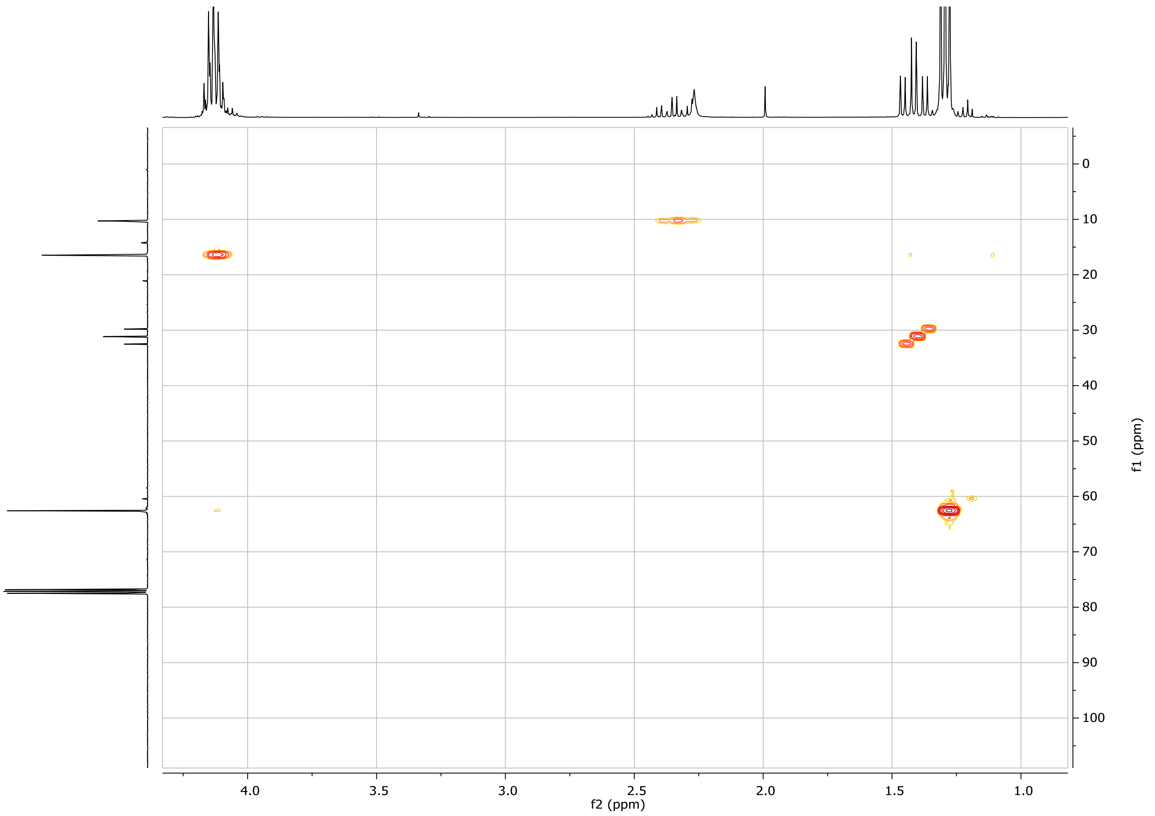

Figure 13. 2D-NMR HMBC of **4b.**

Tetraethyl (2-((3-hydroxypropyl)thio)ethane-1,1-diyl)bis(phosphonate) 4c

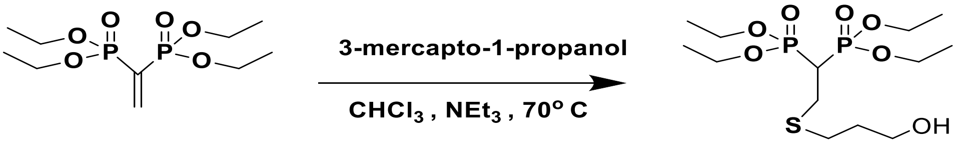

In a 100 mL round bottom flask, vinylidene bisphosphonate tetraethyl ester (VBP, 500 mg, 1.67 mmol) was dispersed in 2.5 mL $CHCl_3$. Then 3- mercapto-1-propanol (146 µL, 1.67 mmol) and triethyl amine (12 µL, 5% in mol) were added to the solution. The apparatus was purged with nitrogen and the reaction mixture was refluxed for overnight. The resulting solution was cooled to room temperature and concentrated with rotavapor. The crude was dissolved in 10 mL CH_2Cl_2 and washed with deionized H_2O (2x 25 mL). The organic phase was dried with Na_2SO_4, filtered and the solvent removed with rotavapor. The crude mixture was purified by flash chromatography with1:1 ethyl acetate/Methanol as eluent. The product was obtained as a colorless liquid (619 mg, 1.58 mmol, 95% yield).

^{1}H NMR (400 MHz, Chloroform-*d*) δ 4.21 – 4.08 (set of m, 8H), 3.68 (t, *J* = 5.9 Hz, 2H), 3.02 (td, *J* = 16.3, 5.9 Hz, 2H), 2.71 – 2.50 (set of m, 3H), 1.81 (p, *J* = 6.5 Hz, 2H), 1.31 (t, *J* = 7.1 Hz, 13H). ù

^{31}P {^{1}H}- NMR (162 MHz, Chloroform-d) δ 21.6 (s, 2P).

^{1}H {^{31}P}- NMR (400 MHz, , Chloroform-d) δ 4.17 – 4.09 (set of m, 8H), 3.69 – 3.64 (t, 2H), 3.00 (d, *J* = 5.7 Hz, 2H), 2.65 (t, *J* = 6.9 Hz, 2H), 2.56 (t, *J* = 5.1 Hz, 1H), 1.82 – 1.74 (p, 2H), 1.28 (t, *J* = 6.9 Hz, 12H).

^{13}C NMR (101 MHz, Chloroform-d) δ 63.06 (d, J = 6.9 Hz), 62.91 (d, J = 6.8 Hz), 38.95 (t, J = 131.8 Hz), 31.90 (s), 29.41 (S), 27.68 (t, J = 4.8 Hz), 16.44 (d, J = 6.4 Hz).

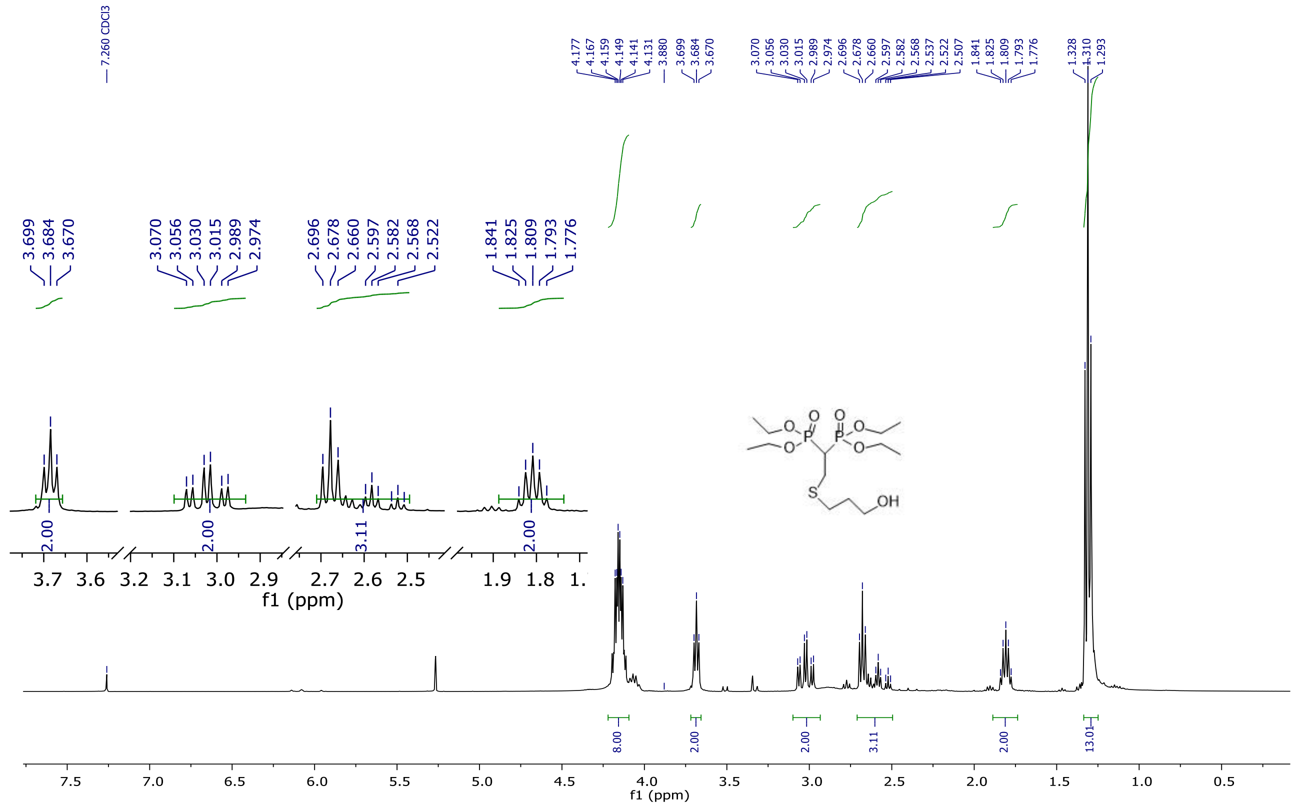

Figure 14. ^{1}H-NMR of **4c.**

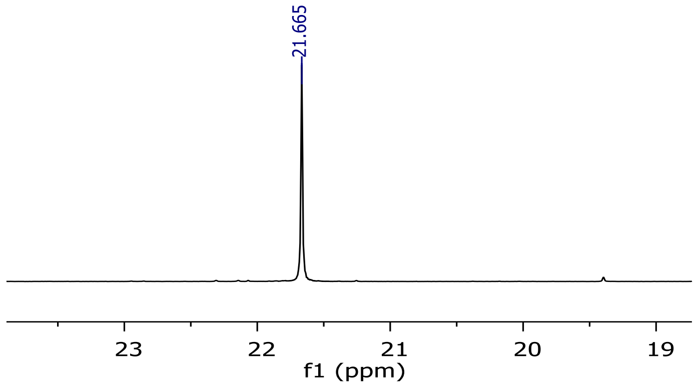

Figure 15. ^{31}P NMR of **4c**.

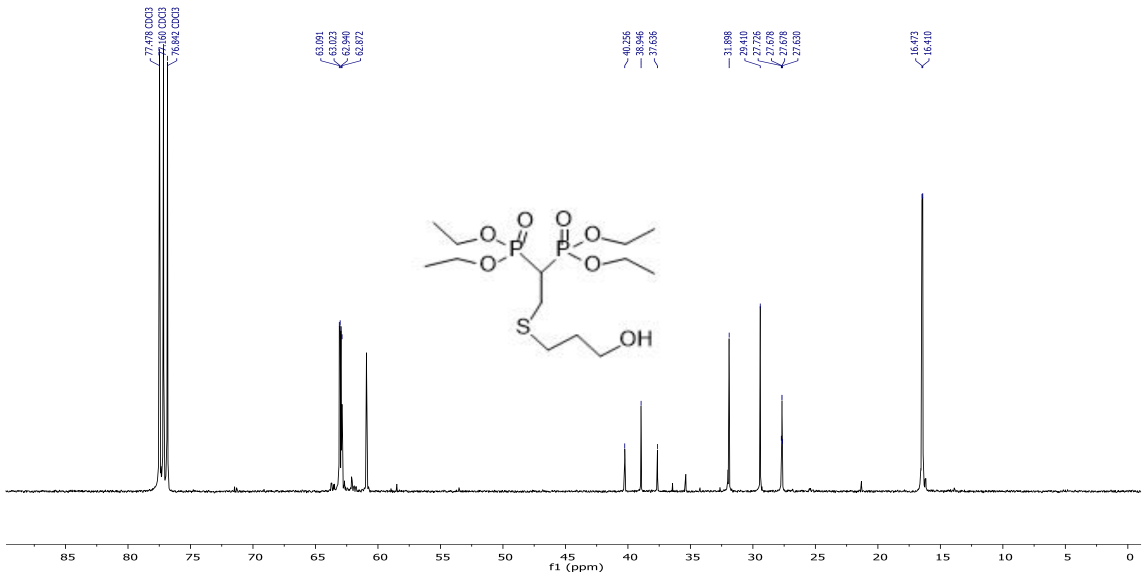

Figure 16. ^{13}C-NMR of **4c**.

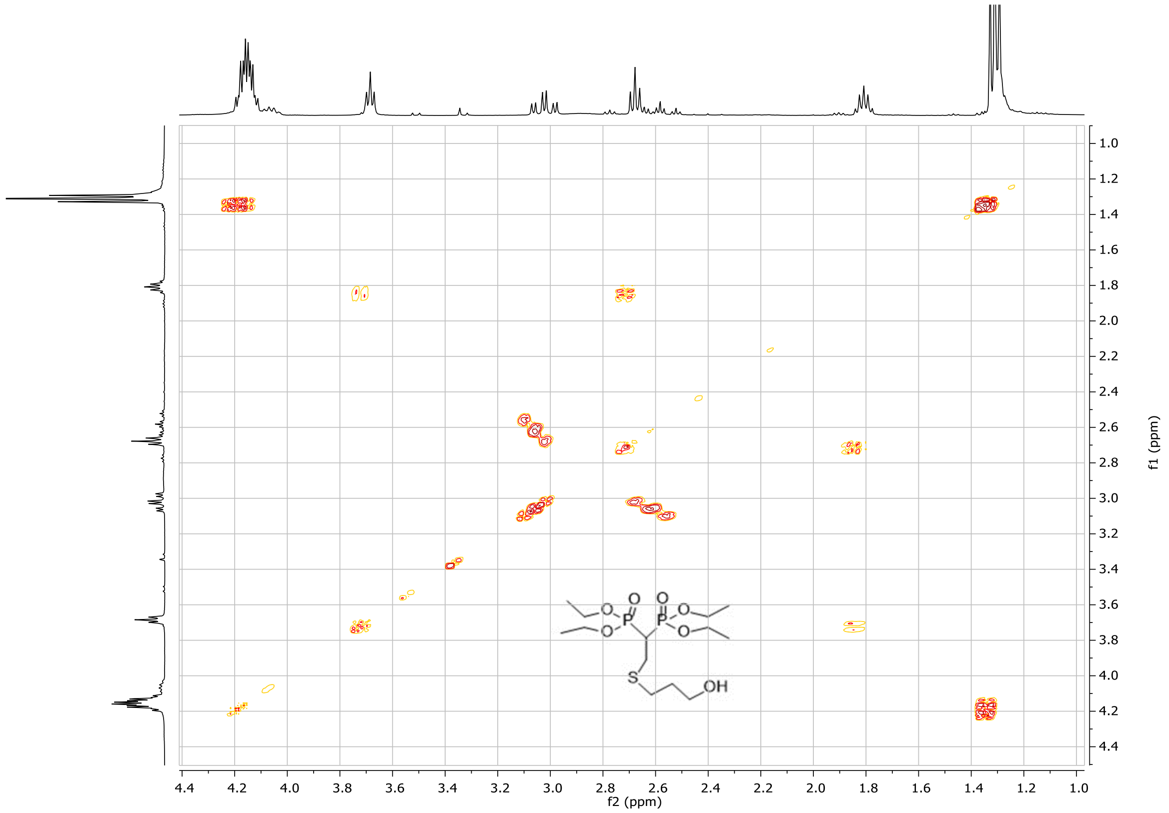

Figure 17. 2D-NMR COSY of **4c**.

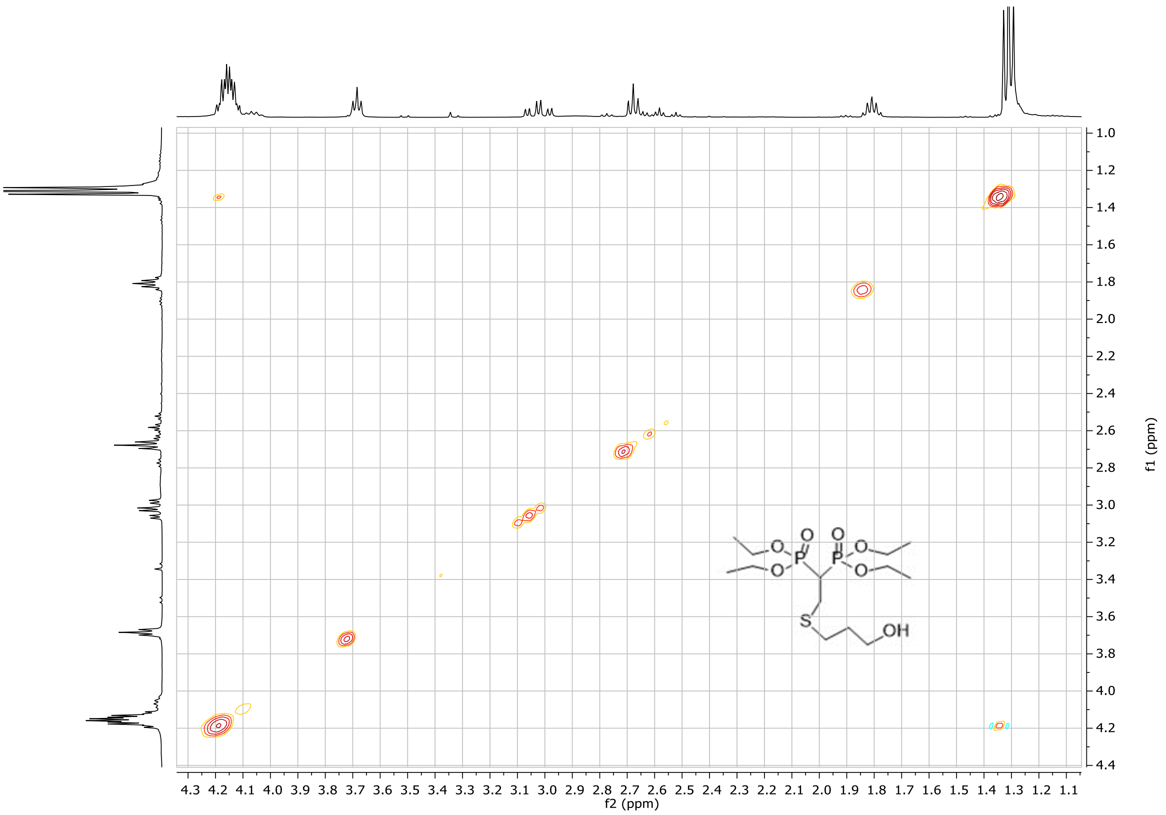

Figure 18. 2D-NMR NOESY of **4c**.

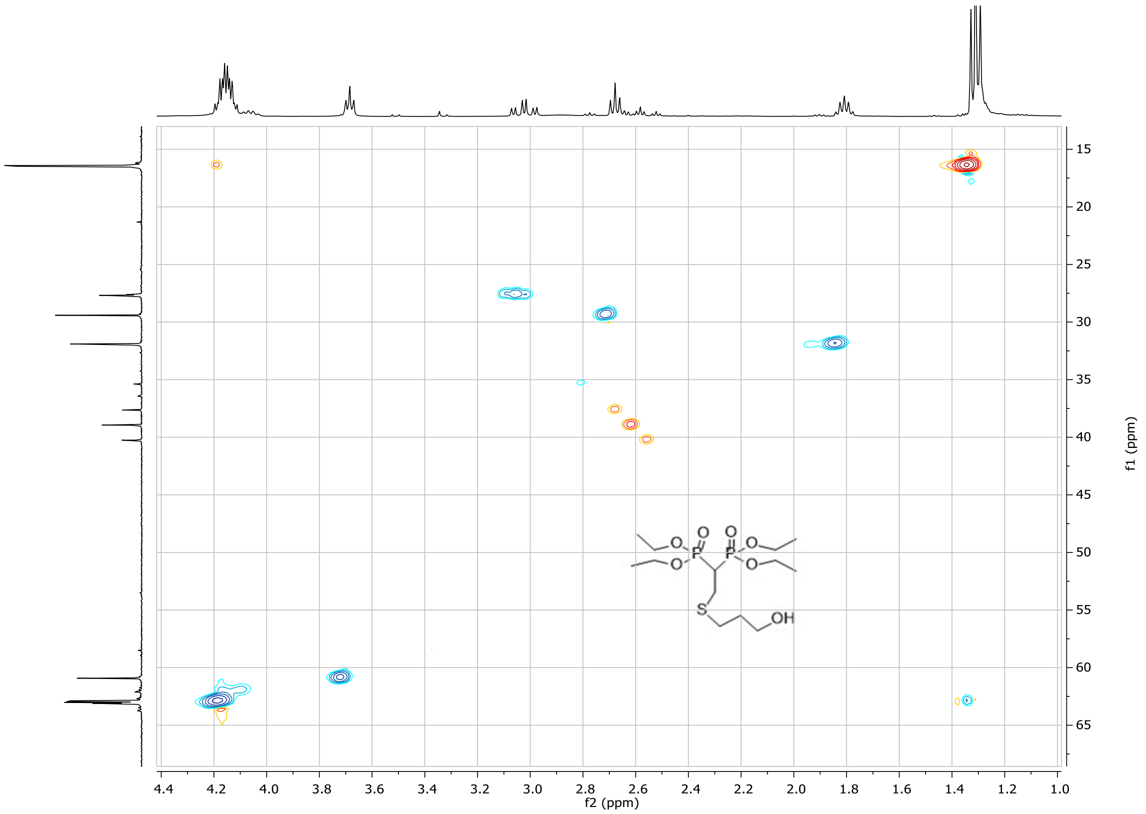

Figure 19. 2D-NMR HSQC of **4c**.

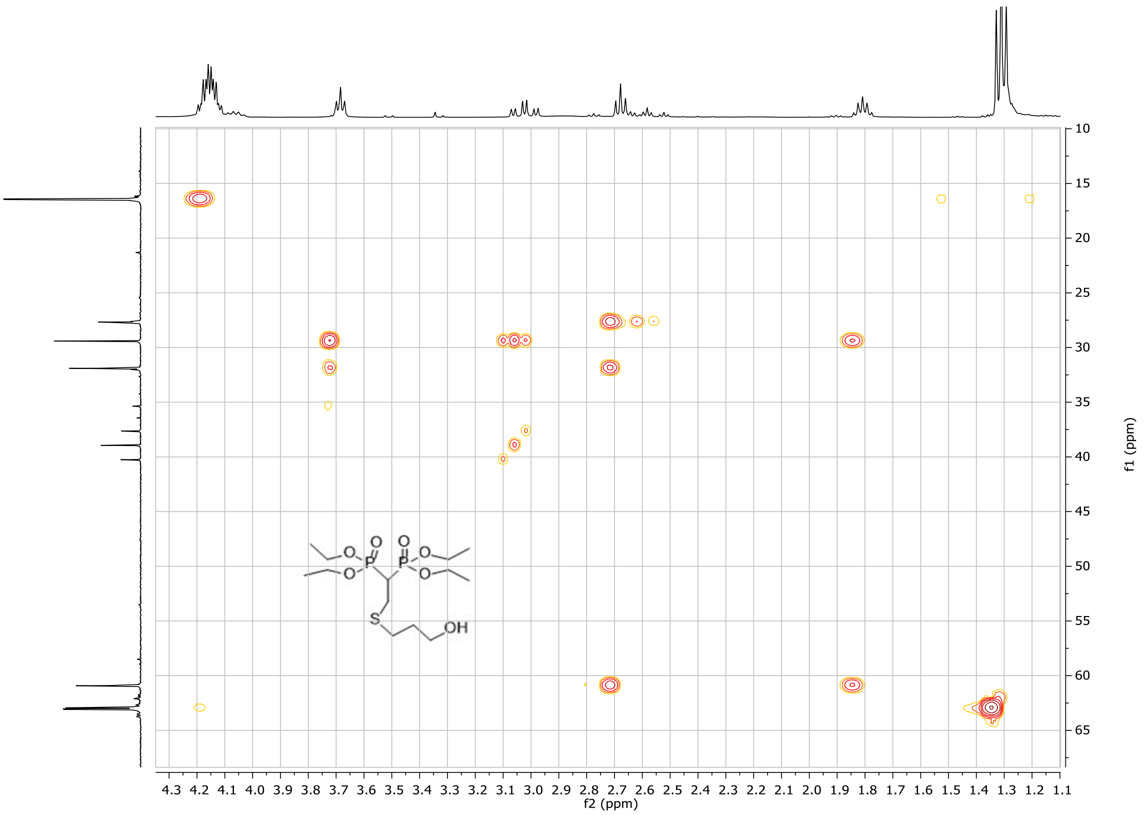

Figure 20. 2D-NMR HMBC of **4c**.

Synthesis of 2-(Boc-amino) ethanethiol

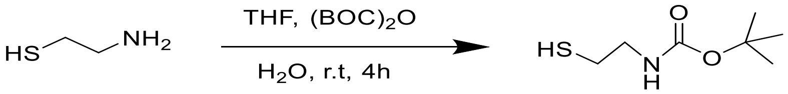

In a 500 mL round bottle flask equipped with magnetic stirring bar, a solution of 2-amino ethanol (200 mg, 2.6 mmol) in water (3 mL) and a THF solution of di-tert-butyl decarbonate $(BOC)_2O$ (597 µL, 2.6 mmol) were added. The reaction mixture was stirred under N_2 atmosphere at room temperature for 4 h. Then solution was extracted with 10 mL of CH_2Cl_2 and washed twice with 25 mL of water. The organic phase was dried with Na_2SO_4, filtered and the solvent removed with rotavapor. The product was obtained as a colorless liquid (451 mg, 2.49 mmol, 98% yield).

^{1}H NMR (400 MHz, Chloroform-*d*) δ 3.21 (q, *J* = 6.1 Hz, 2H), 2.56 (q, *J* = 8.0, 7.3 Hz, 2H), 1.36 (s, 7H), 1.31 (t, *J* = 8.5 Hz, 1H).

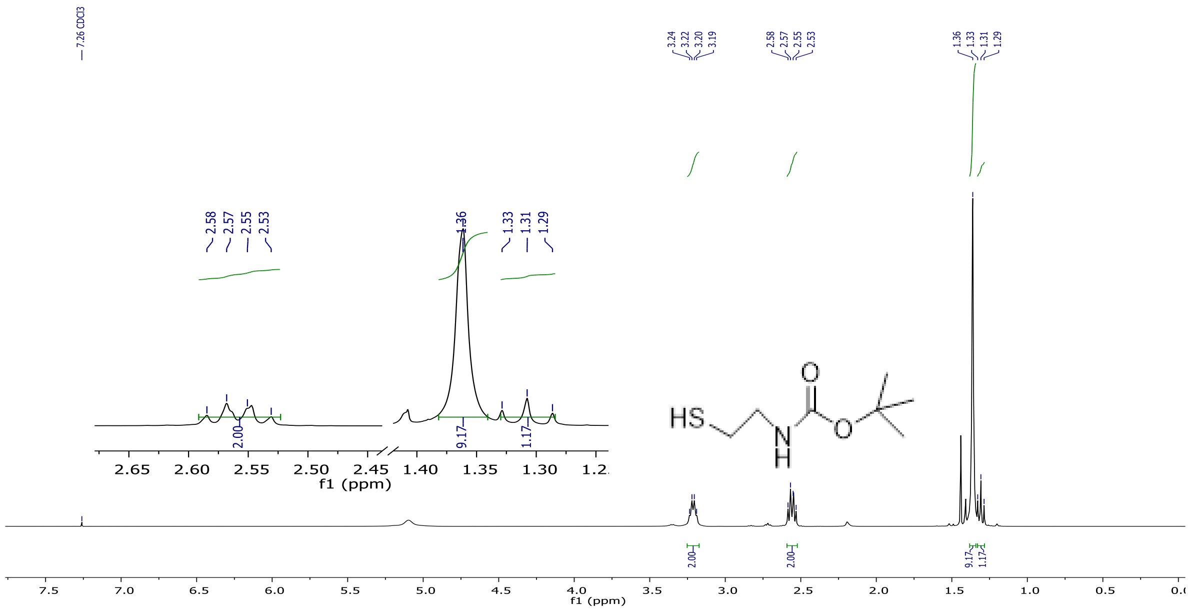

Figure 21. ^{1}H-NMR of 2-(Boc-amino) ethanethiol.

Tert-butyl (2-((2,2-bis(diethoxyphosphoryl)ethyl)thio)ethyl)carbamate 4d

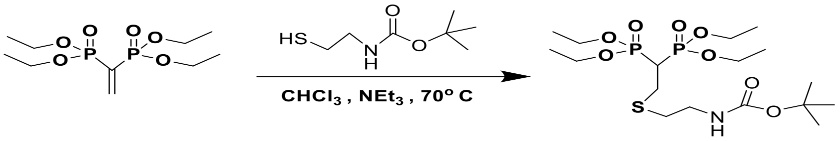

In a 100 mL round bottom flask, vinylidene bisphosphonate tetraethyl ester (VBP, 500 mg, 1.67 mmol) was dispersed in 2.5 mL $CHCl_3$. Then (2-boc-amino)-ethanethiol (301 µL, 1.67 mmol) and triethyl amine (12 µL, 5% in mol) were added to the solution. The apparatus was purged with nitrogen and the reaction mixture was refluxed overnight. The resulting solution was cooled to room temperature and concentrated with rotavapor. The crude was dissolved in 10 mL CH_2Cl_2 and washed with deionized H_2O (2x 25 mL). The organic phase was dried with Na_2SO_4, filtered and the solvent removed with rotavapor. The crude mixture was purified by flash chromatography with 1:1 ethyl acetate/Methanol as eluent. The product was obtained as a colorless liquid (678 mg, 1.42 mmol, 85% yield).

^{1}H NMR (400 MHz, Chloroform-*d*) δ 4.24 – 4.13 (set of m, 8H), 3.38 – 3.28 (m, 2H), 3.03 (td, *J* = 16.5, 5.8 Hz, 2H), 2.68 (t, *J* = 6.2 Hz, 2H), 2.57 (tt, *J* = 24.0, 5.9 Hz, 1H), 1.42 (s, 9H), 1.33 (t, *J* = 7.1 Hz, 12H). ^{31}P{^{1}H}- NMR (162 MHz, Chloroform-*d*) δ 21.56 (s, 2P).

^{1}H {^{31}P}- NMR (400 MHz, Chloroform-*d*) δ 4.26 – 4.11 (set of m, 8H), 3.32 (set of m, 2H), 3.03 (d, *J* = 5.7 Hz, 2H), 2.68 (t, *J* = 6.1 Hz, 2H), 2.57 (t, *J* = 5.8 Hz, 1H), 1.42 (s, 9H), 1.33 (t, *J* = 7.1 Hz, 12H). ^{13}C NMR (101 MHz, Chloroform-*d*) δ 63.09 (d, *J* = 6.7 Hz), 62.91 (d, *J* = 6.7 Hz), 39.47 (s), 39.17 (t, *J* = 131.9 Hz), 33.53 (s), 28.52 (s), 27.51 (t, *J* = 4.9 Hz), 16.50 (d, *J* = 6.2 Hz).

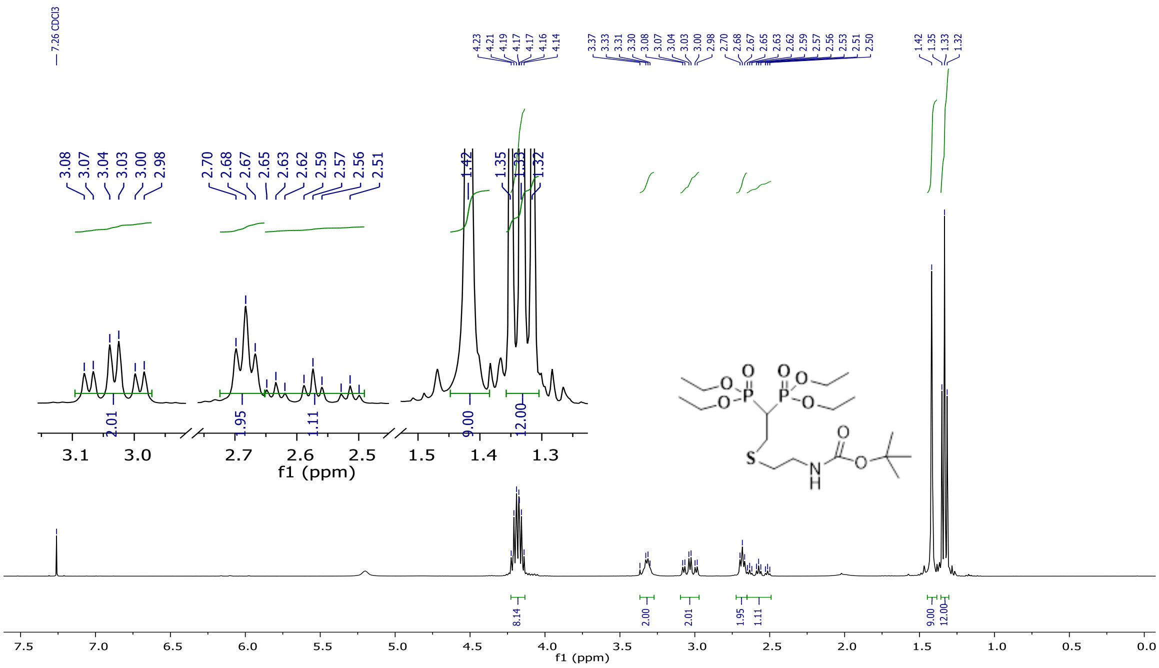

Figure 22. ^{1}H-NMR of **4d**.

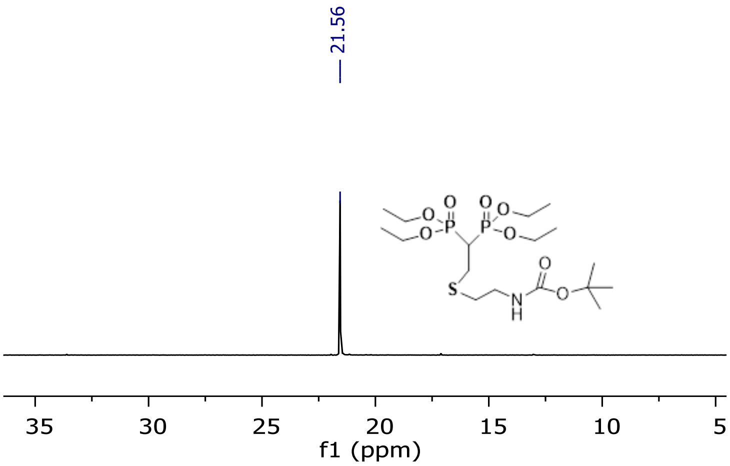

Figure 23. ^{31}P-NMR of **4d**.

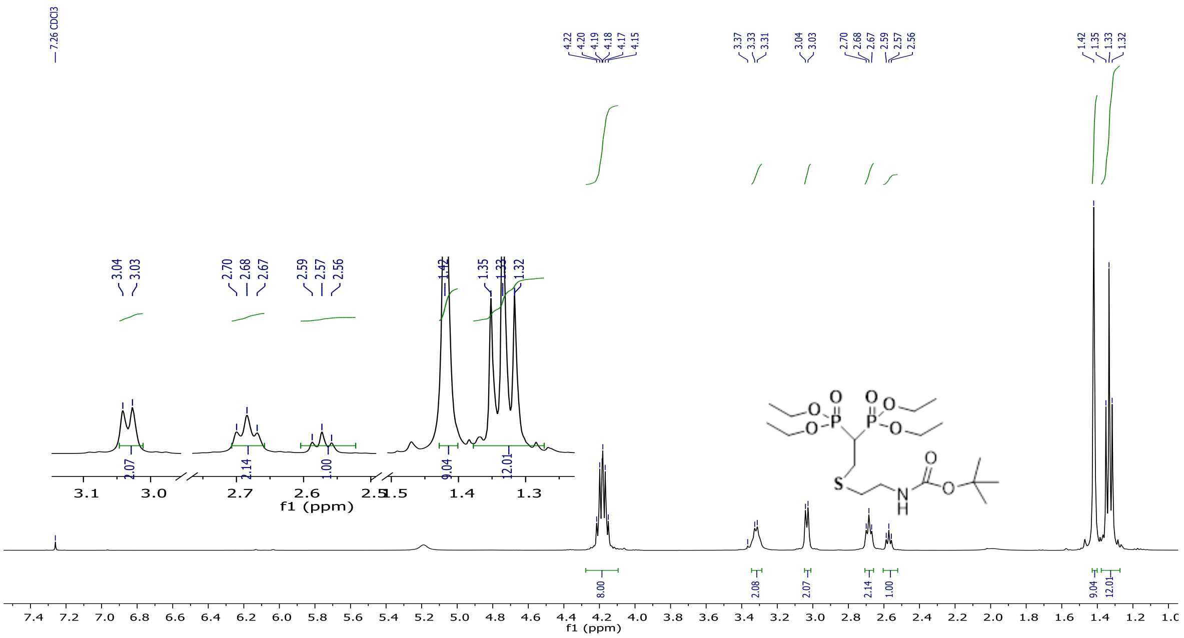

Figure 24. $^{1}H\{^{31}P\}$-NMR of **4d**.

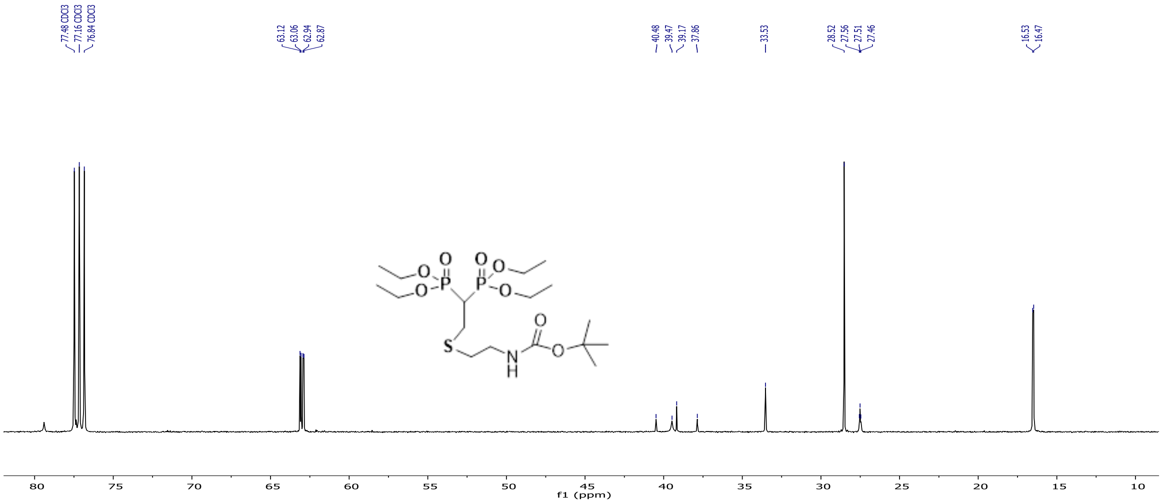

Figure 25. ^{13}C NMR of **4d**.

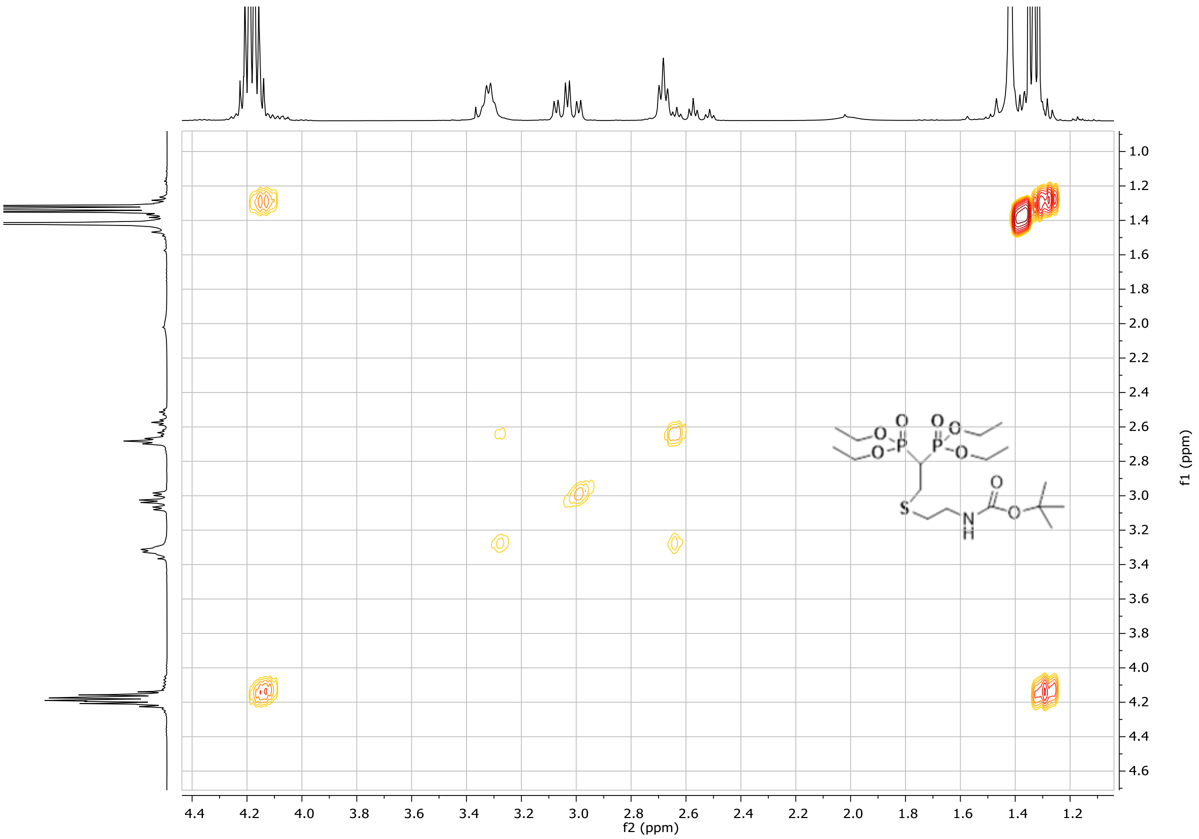

Figure 26. 2D-NMR COSY of **4d**.

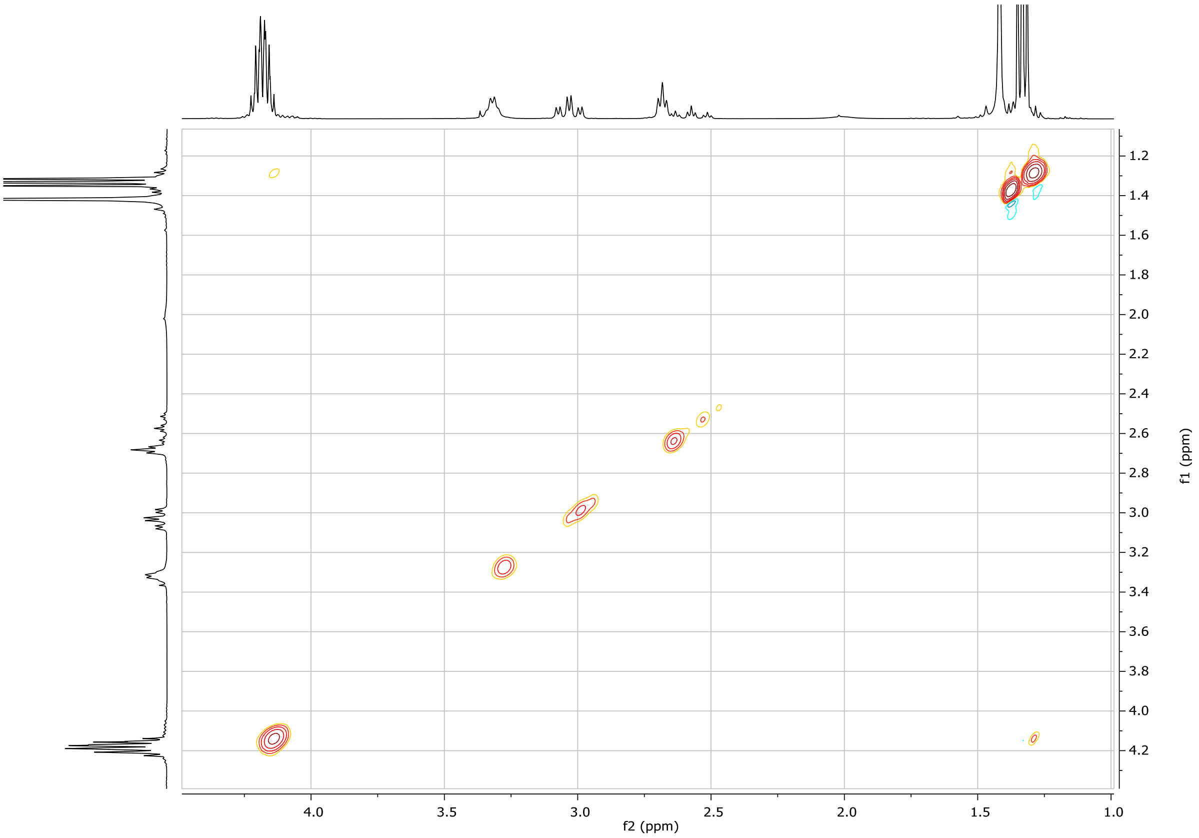

Figure 27. 2D-NMR NOESY of **4d**.

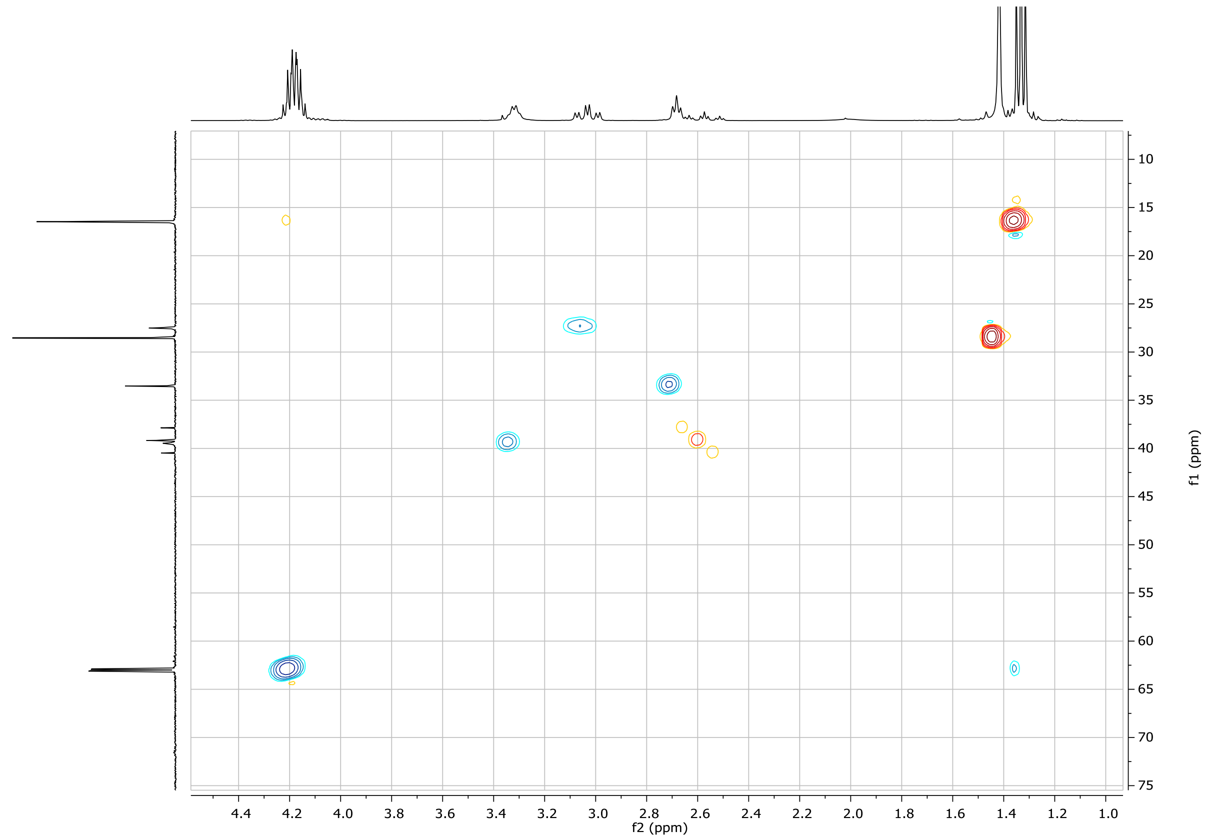

Figure 28. 2D-NMR HSQC of **4d**.

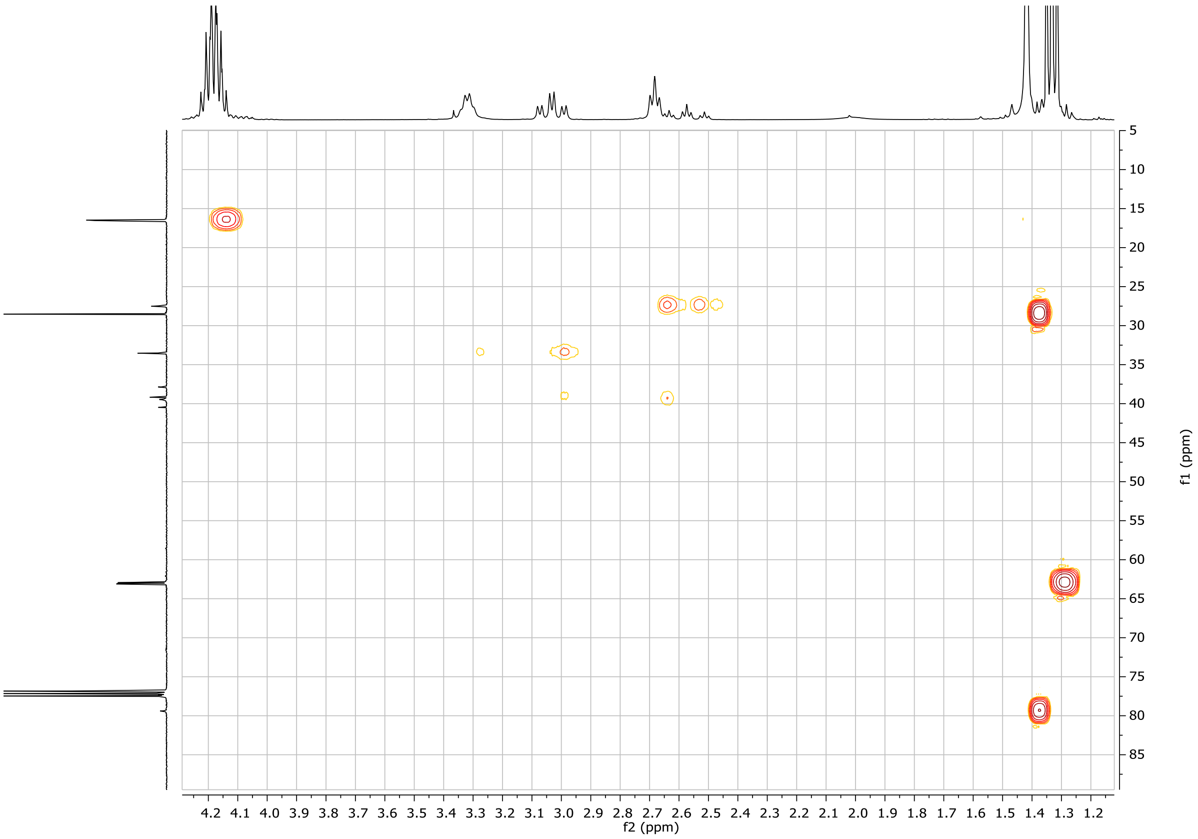

Figure 29. 2D-NMR HMBC of **4d**.

Tetraethyl (2-((2-aminoethyl)thio)ethane-1,1-diyl)bis(phosphonate) 5d

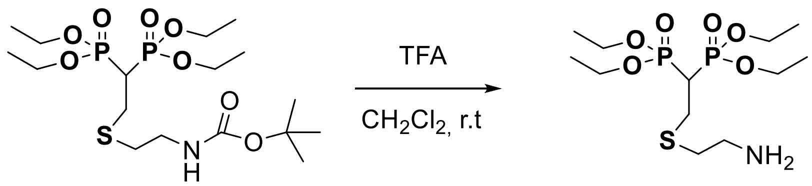

In a 100 mL round bottom flask, tetraethyl 2-{(2-boc-amino) ethanethiol} ethane-1,1-diyldiphosphonate (500 mg, 1.05 mmol) was added in 3 mL of CH_2Cl_2. Then 4 mL of trifluoracetic acid (TFA) was added slowly and the reaction mixture was left under stirring for 1.5 h at room temperature. The crude treated under vacuum. The product was obtained as colorless liquid (388 mg, 1.03 mmol, 98% yield).

^{1}H NMR (400 MHz, Chloroform-*d*) δ 4.27 – 4.12 (set of m, 8H), 3.26 (set of m, 2H), 3.06 (td, *J* = 17.2, 6.1 Hz, 2H), 2.96 (t, *J* = 6.0 Hz, 2H), 2.78 (tt, *J* = 24.4, 6.0 Hz, 1H), 1.35 (t, *J* = 7.1 Hz, 12H). ^{31}P{^{1}H}- NMR (162 MHz, Chloroform-*d*) δ 20.93 (s ,2p).

^{1}H {^{31}P}-NMR (400 MHz, Chloroform-*d*) δ 4.20 (p, *J* = 7.1 Hz, 8H), 3.28 (set of m, 2H), 3.07 (d, *J* = 6.0 Hz, 2H), 2.96 (t, *J* = 5.9 Hz, 2H), 2.79 (t, *J* = 5.9 Hz, 1H), 1.35 (t, *J* = 7.0 Hz, 12H). ^{13}C NMR (101 MHz, Chloroform-*d*) δ 64.47 (d, *J* = 7.1 Hz), 64.29 (d, *J* = 7.0 Hz), 38.77 (s), 37.55 (t, *J* = 136.2 Hz), 30.25 (S), 27.07 (t, *J* = 5.0 Hz), 16.20 (d, *J* = 5.6 Hz).

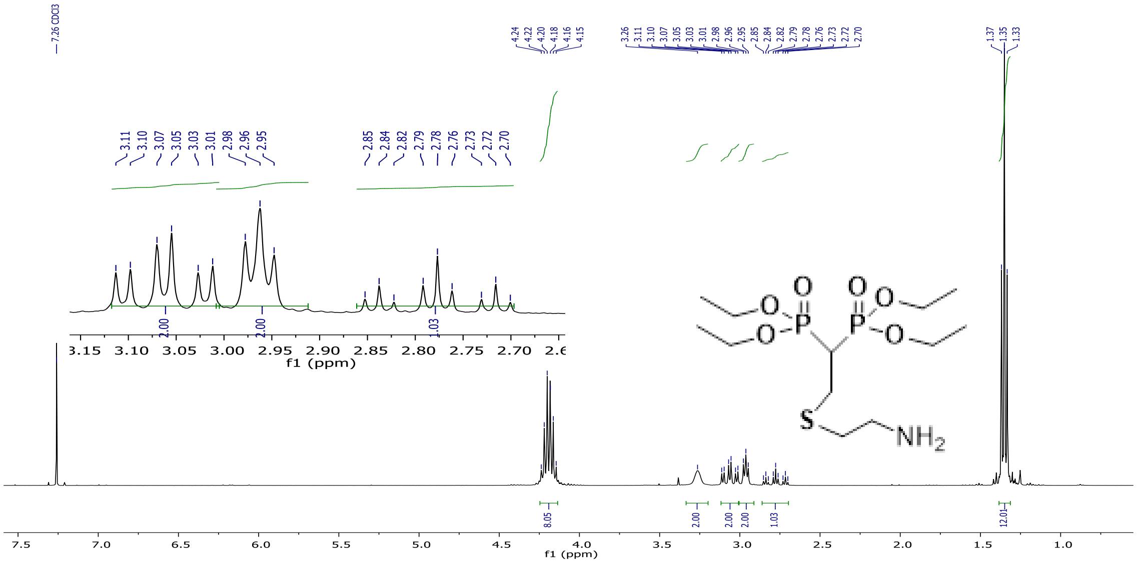

Figure 30. ^{1}H-NMR of **5d.**

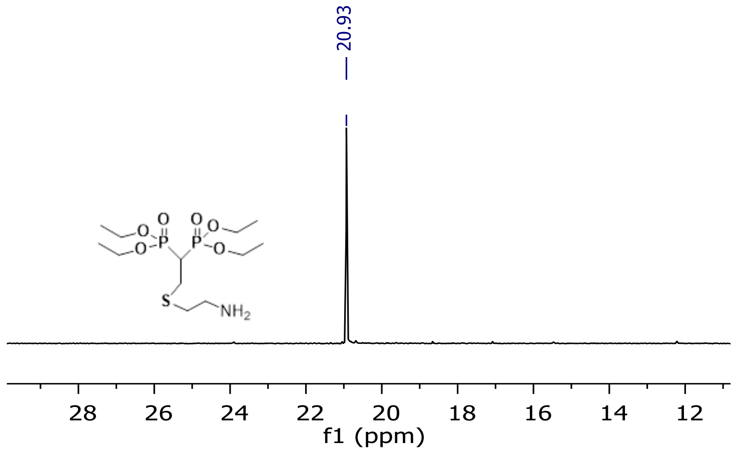

Figure 31. ^{31}P-NMR of **5d**.

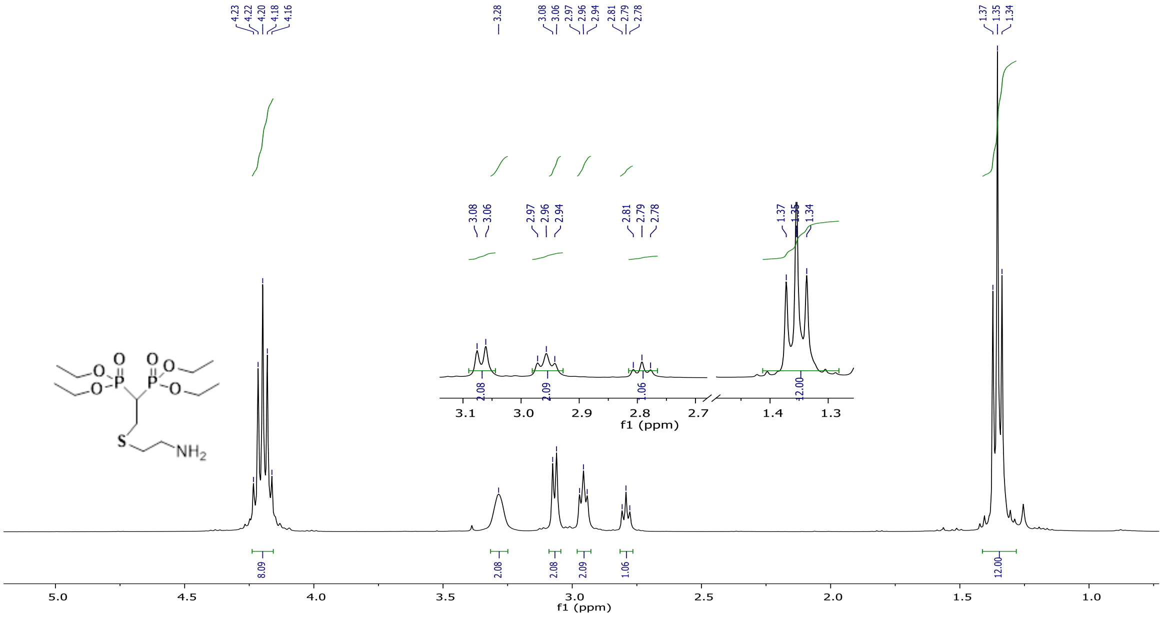

Figure 32. ^{1}H{^{31}P}-NMR of **5d**.

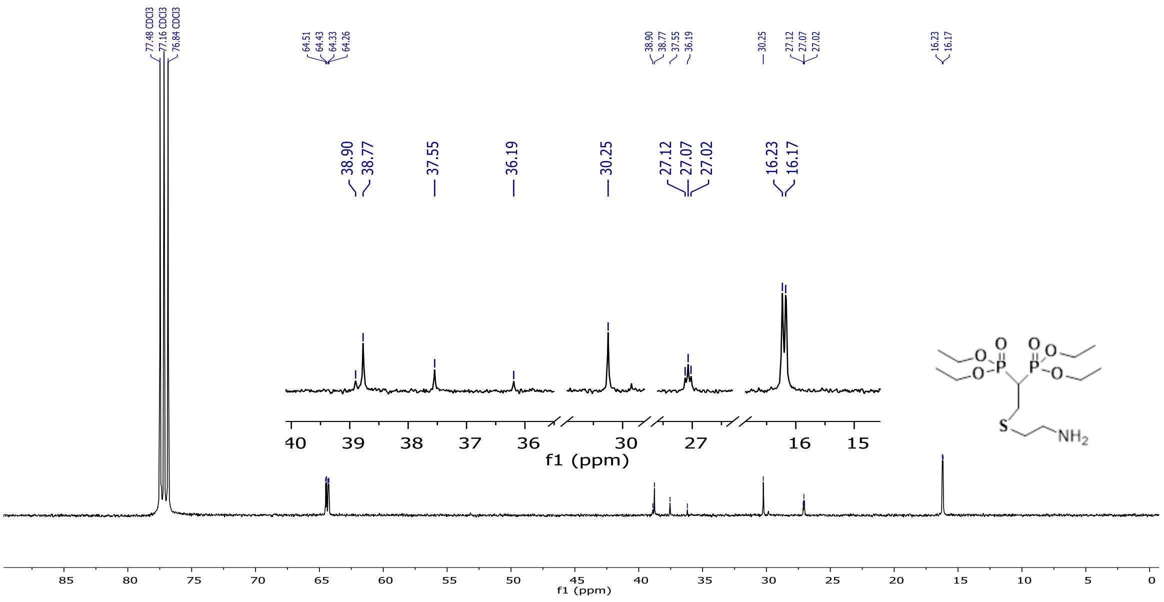

Figure 33. ^{13}C NMR of **5d**.

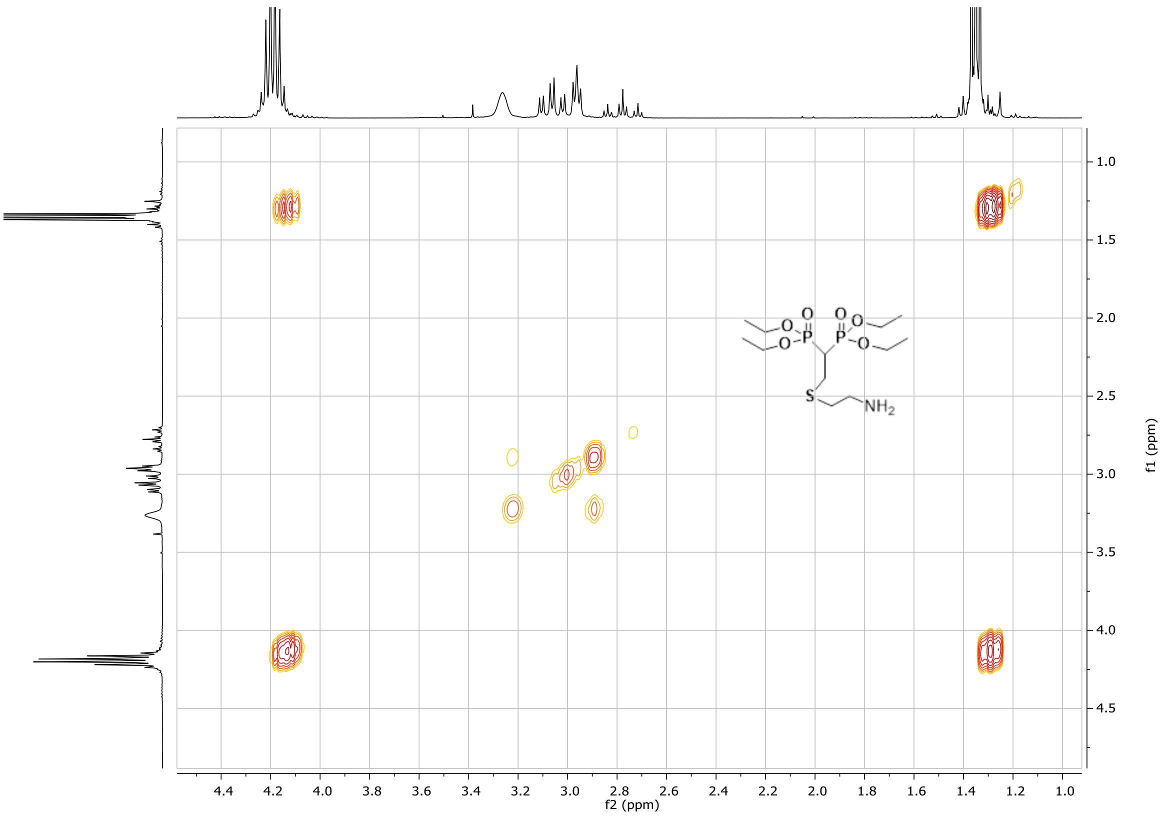

Figure 34. 2D-NMR COSY of **5d**.

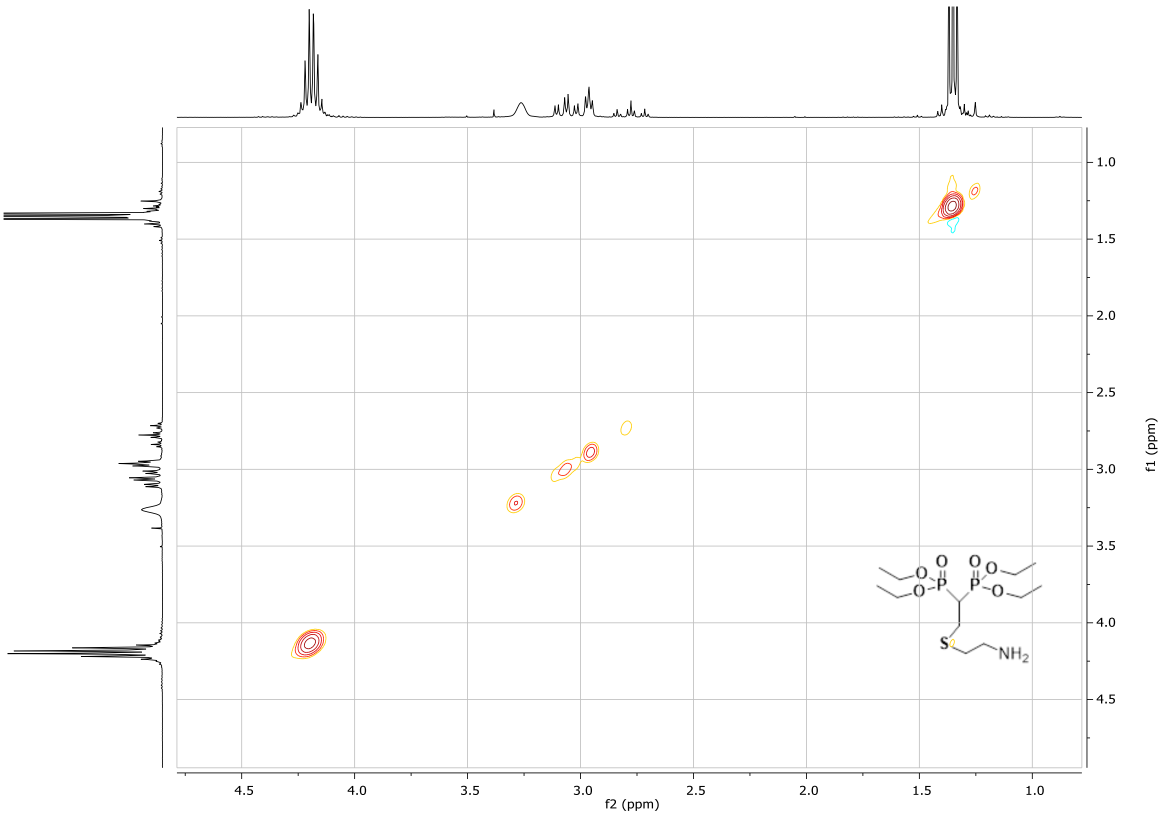

Figure 35. 2D-NMR NOESY of **5d**.

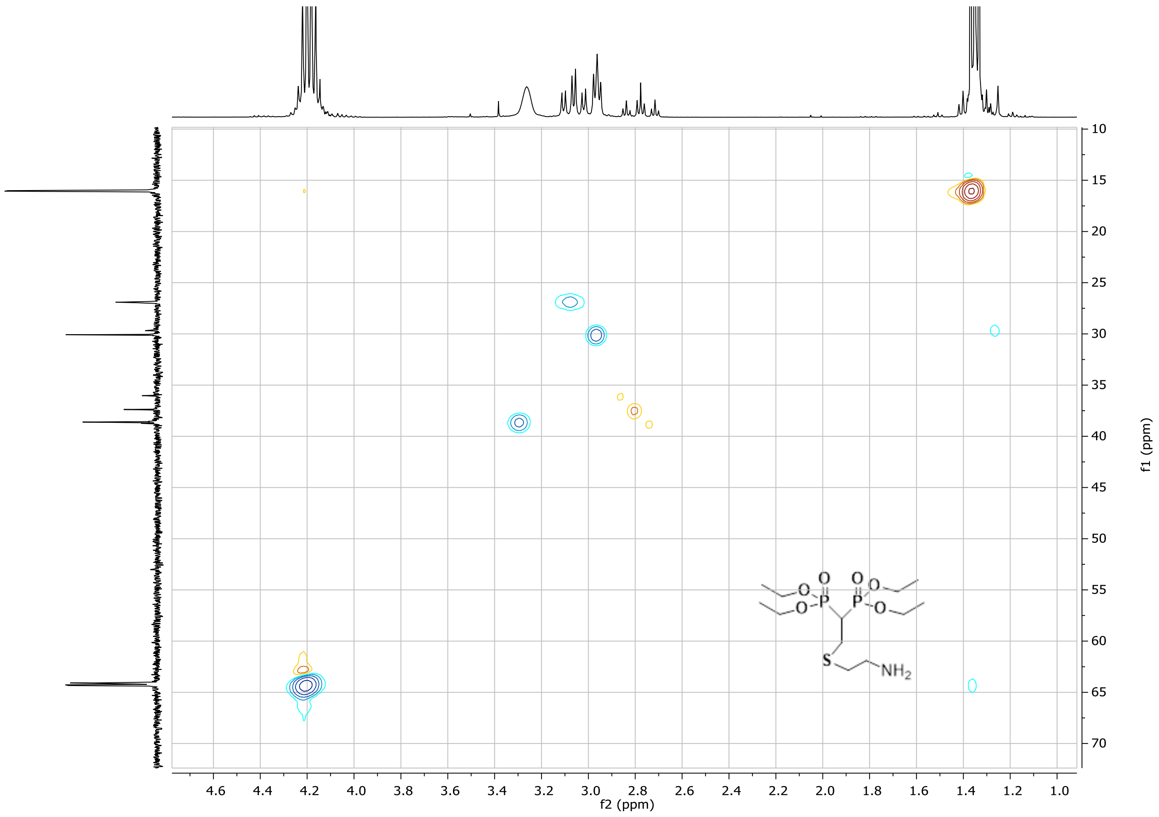

Figure 36. 2D-NMR HSQC of **5d**.

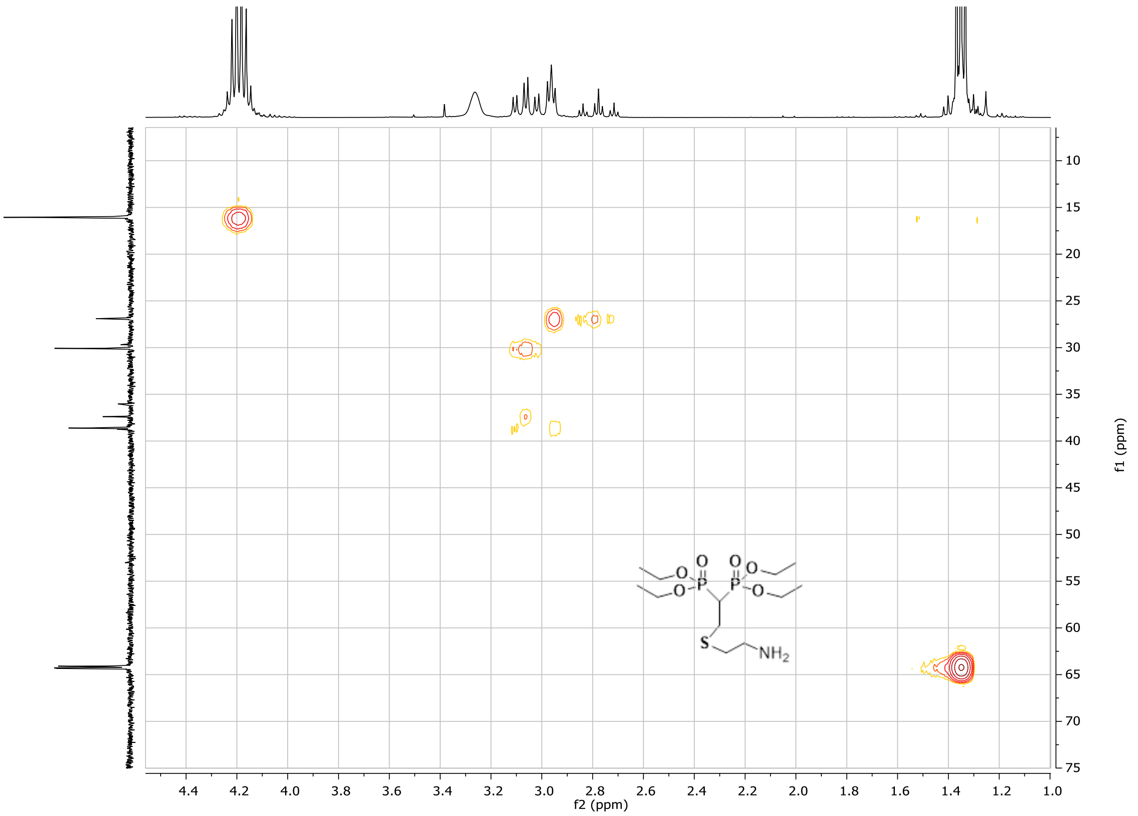

Figure 37. 2D-NMR HMBC of **5d**.

Benzyl 2-((2,2-bis(diethoxyphosphoryl)ethyl)thio)acetate 4e

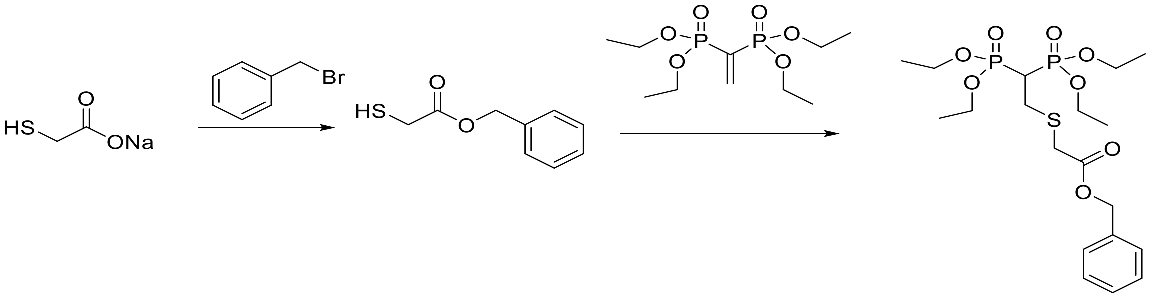

In a double neck 100 mL round bottle flask, sodium thioglycolate (250 mg, 2.19 mmol) was added in 3-4 mL of anhydrous DMF. The apparatus was immediately purged with nitrogen gas. Then benzyl bromide (260 mg, 2.19 mmol) and vinylidene bisphosphonate tetraethyl ester (VBP, 657 mg, 2.19 mmol) were added in solution using needle. The reaction mixture was left at 60°C overnight. After that, a molar equivalent amount of triethyl amine (300 µL) was added to the reaction mixture under inert atmosphere. Then the reaction mixture was left for 24h at 60°C. The reaction mixture was cooled to room temperature and diluted with 20 mL CH_2Cl_2 solvent. The organic phase was dried with Na_2SO_4, filtered and the solvent was removed with rotavapor. The crude mixture was purified by flash chromatography with1:1 ethyl acetate/Methanol as eluent. The product was obtained as a yellow liquid (739 mg, 1.53 mmol, 70% yield).

^{1}H NMR (400 MHz, Chloroform-*d*) δ 7.40 – 7.27 (set of m, 5H), 5.15 (s, 2H), 4.23 – 4.07 (set of m, 8H), 3.35 (s, 2H), 3.17 (td, *J* = 16.1, 6.0 Hz, 2H), 2.74 (tt, *J* = 24.0, 6.0 Hz, 1H), 1.31 (t, *J* = 7.1 Hz, 12H).

^{31}P{^{1}H}-NMR (162 MHz, Chloroform-*d*) δ 21.27 (s, 2P).

$^{1}H\{^{31}P\}$-NMR (400 MHz, Chloroform-*d*) δ 7.39 – 7.27 (set of m, 5H), 5.15 (s, 2H), 4.16 (q, *J* = 7.0 Hz, 8H), 3.35 (s, 2H), 3.17 (d, *J* = 6.0 Hz, 2H), 2.74 (t, *J* = 6.0 Hz, 1H), 1.31 (t, *J* = 7.1 Hz, 12H).

^{13}C NMR (101 MHz, Chloroform-*d*) δ 128.70 (s), 128.50 (s), 128.39 (s), 67.26 (s), 63.19 (d, *J* = 6.6 Hz), 63.03 (d, *J* = 6.7 Hz), 38.64 (t, *J* = 131.8 Hz), 34.88 (s), 28.81 (t, *J* = 4.9 Hz), 16.45 (d, *J* = 6.2 Hz).

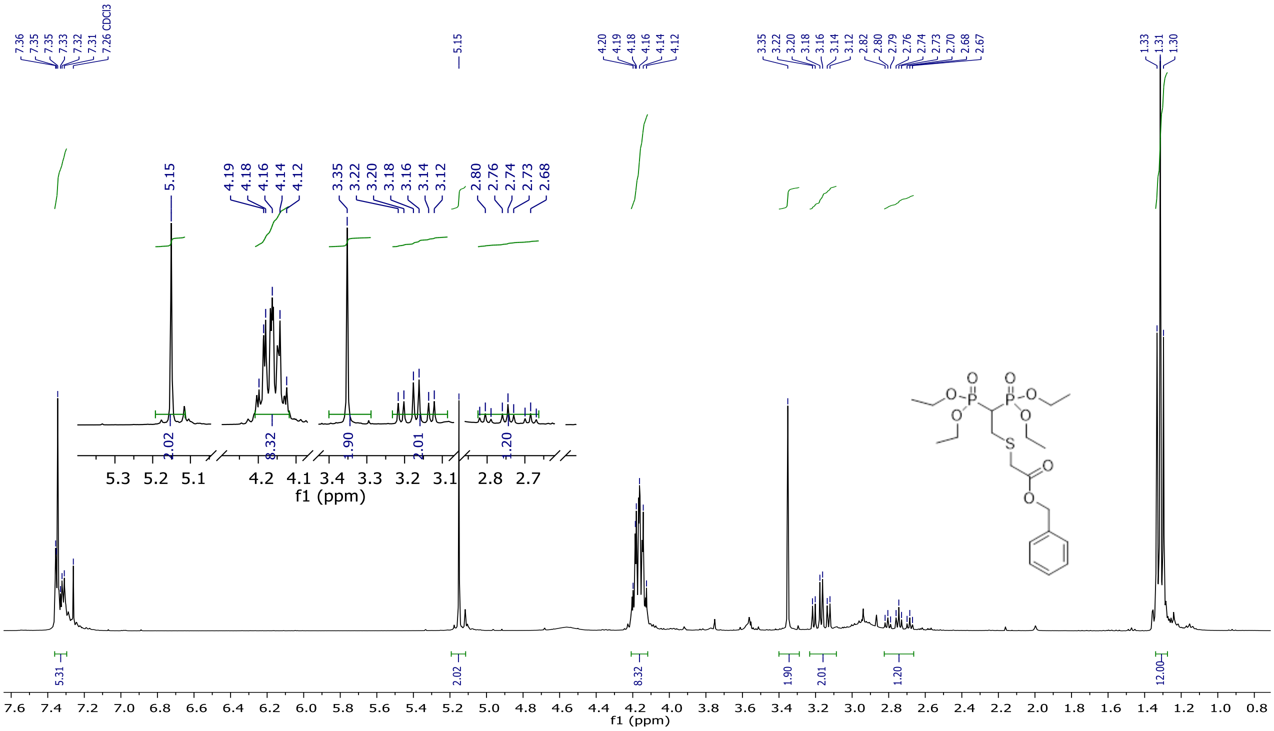

Figure 38. ^{1}H NMR of **4e**.

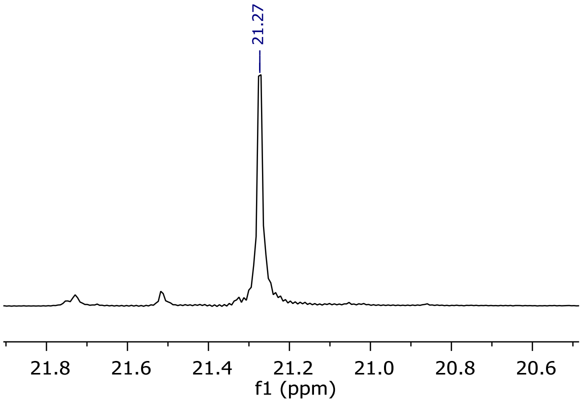

Figure 39. ^{31}P $\{^{1}H\}$-NMR of **4e**.

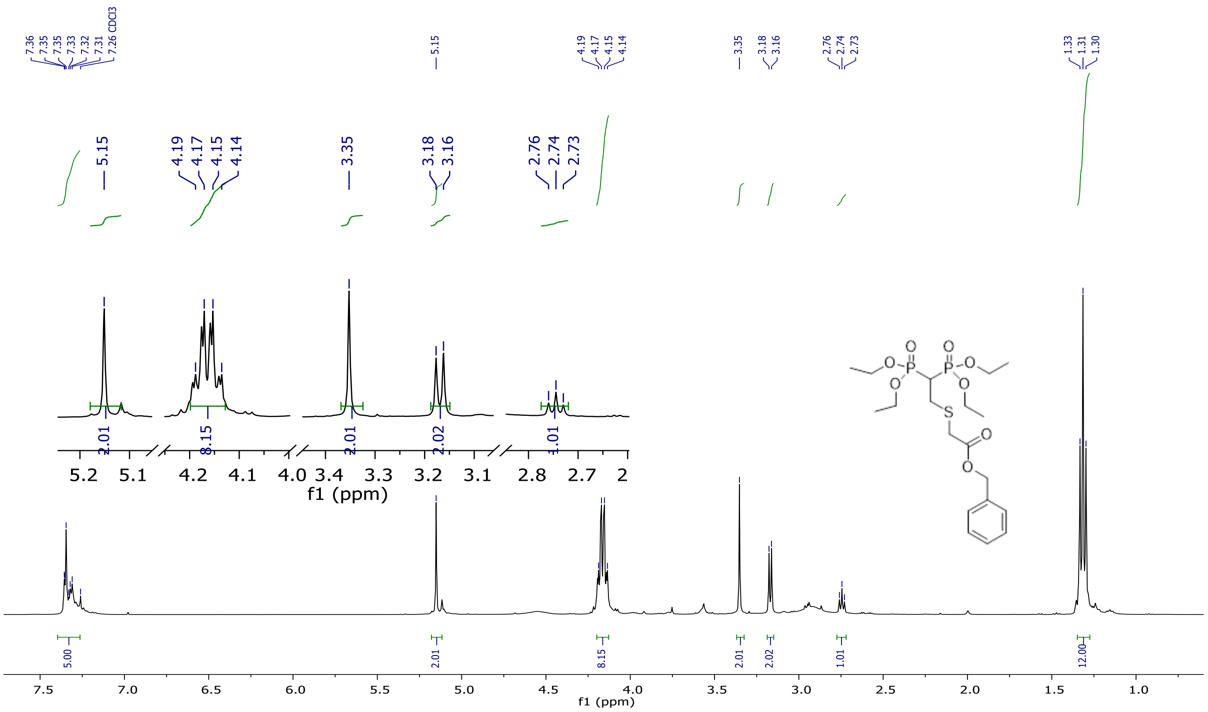

Figure 40. $^{1}H\ \{^{31}P\}$ -NMR of **4e**.

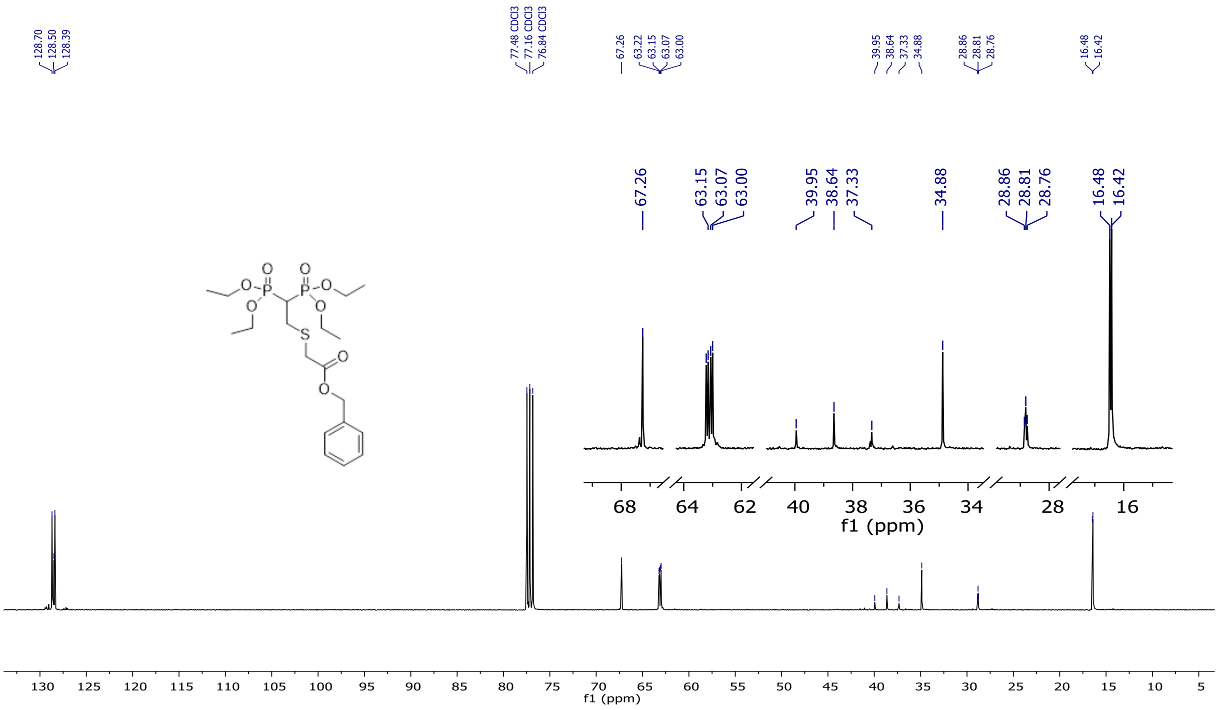

Figure 41. ^{13}C -NMR of **4e**.

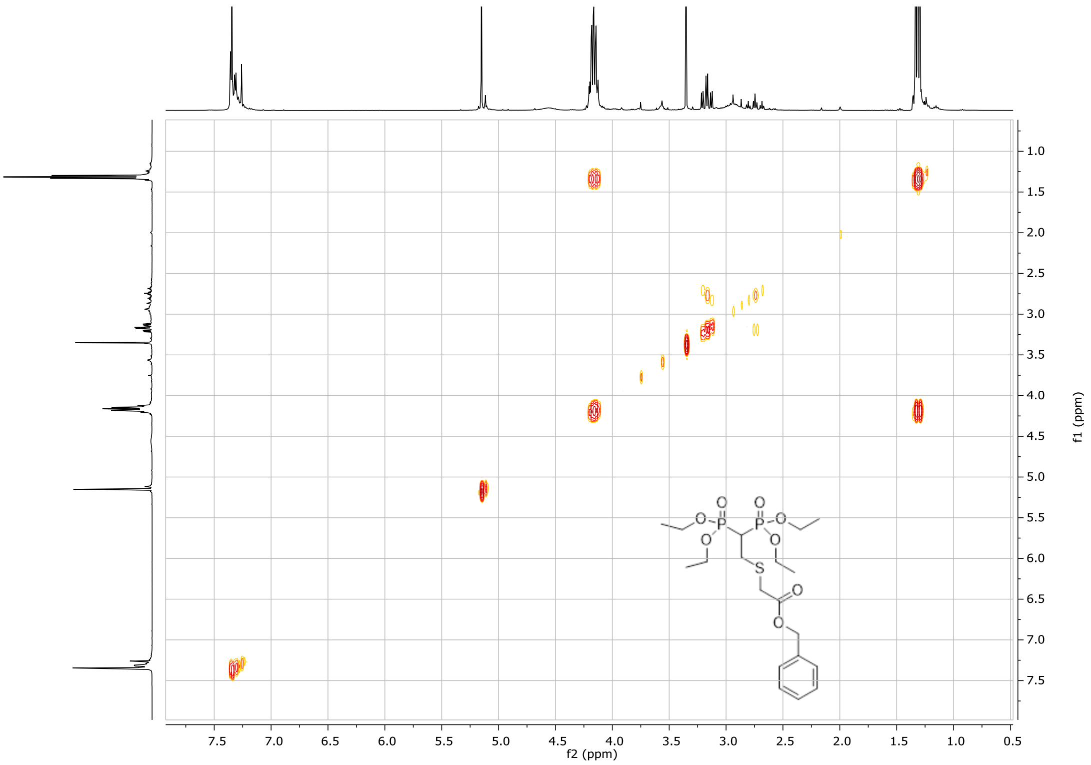

Figure 42. 2D-NMR COSY of **4e**.

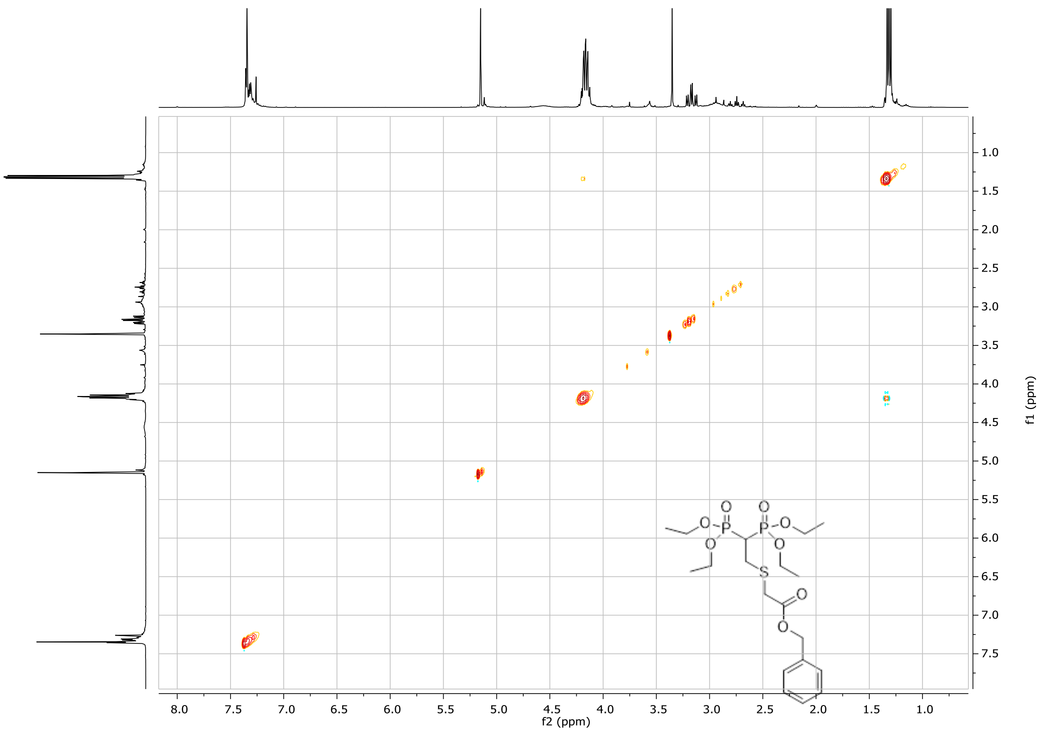

Figure 43. 2D-NMR NOESY of **4e**.

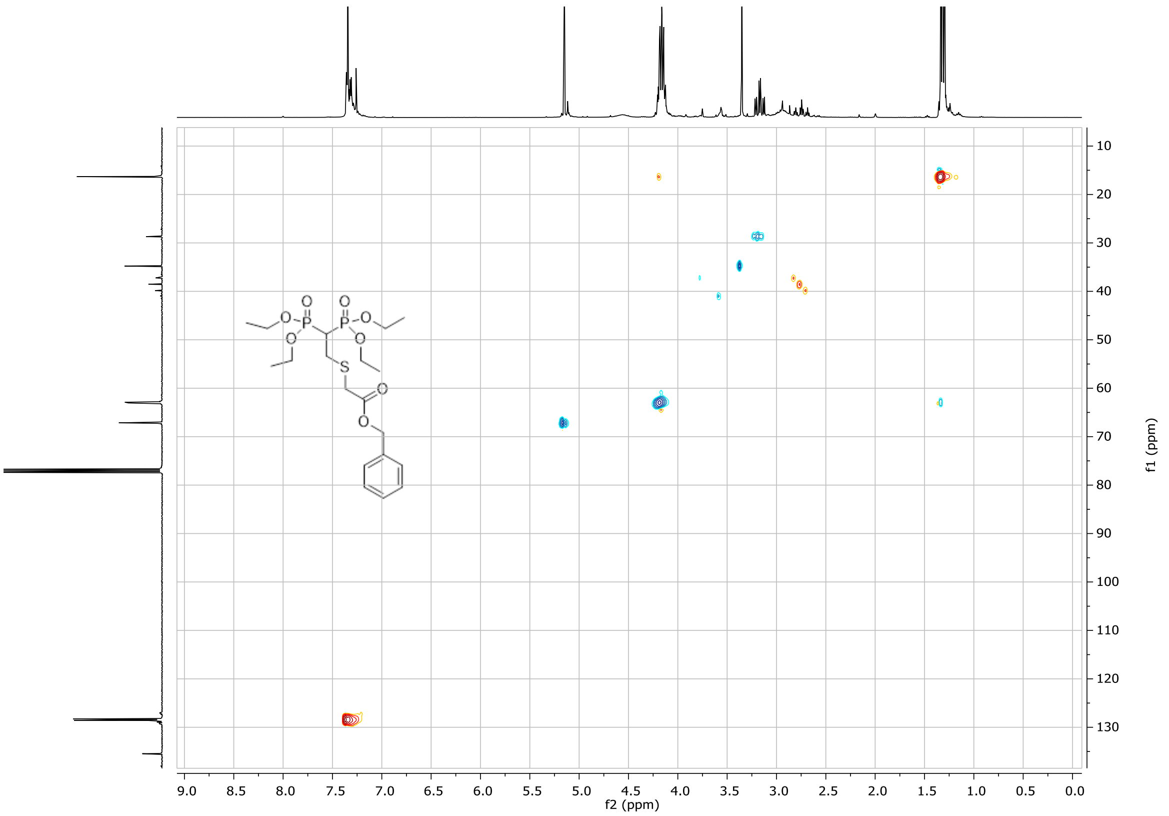

Figure 44. 2D-NMR HSQC of **4e**.

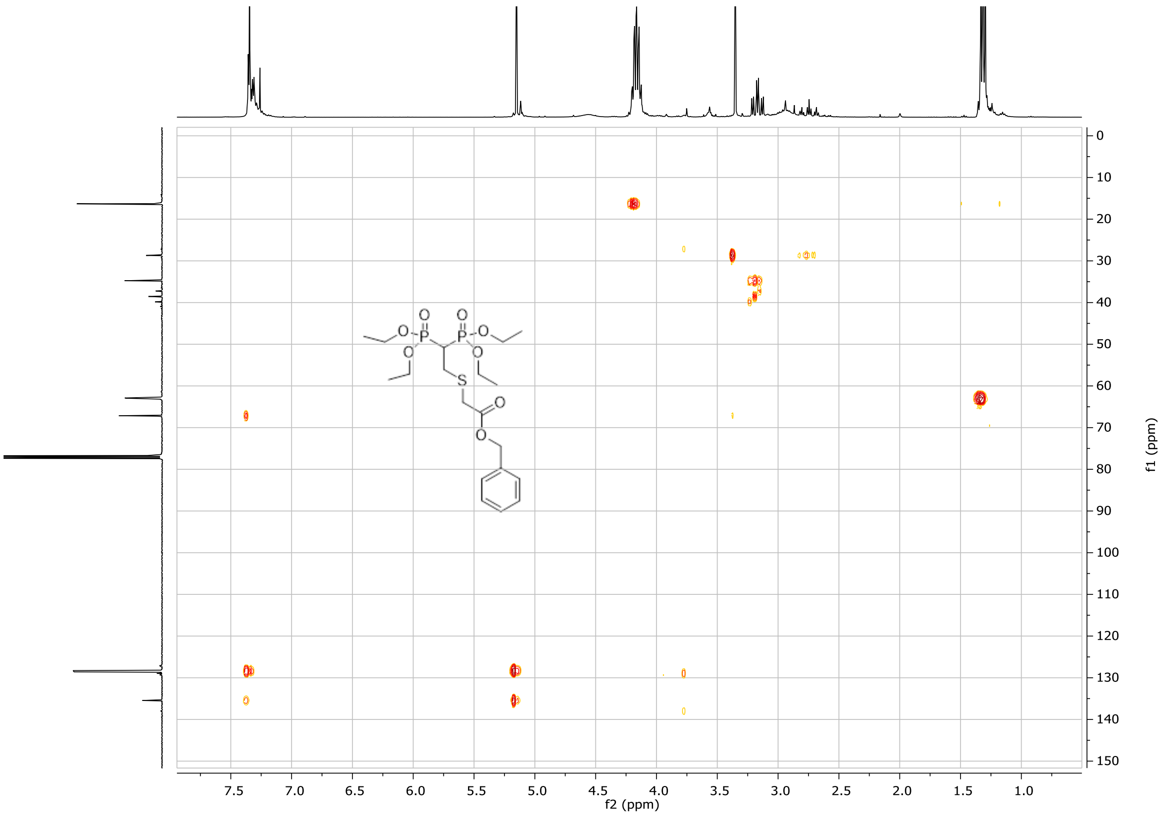

Figure 45. 2D-NMR HMBC of **4e**.

Tetraethyl (2-(allylthio)ethane-1,1-diyl)bis(phosphonate) 4f

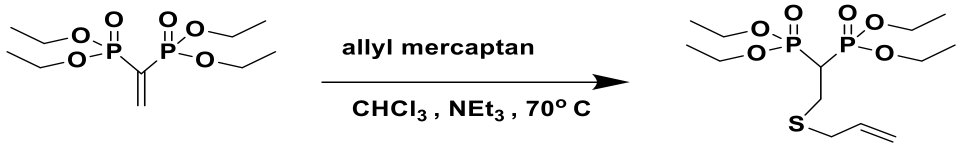

In a 100 mL round bottom flask equipped with magnetic stirring bar, vinylidene bisphosphonate tetra-ethyl ester (VBP, 500 mg, 1.67 mmol) was dispersed in 2.5 mL $CHCl_3$. Then allyl mercaptan (137 µL, 1.67 mmol) and triethyl amine (12 µL, 5% in mol) were added to the solution. The apparatus was purged with nitrogen and the reaction mixture was refluxed overnight. The resulting solution was cooled to room temperature and concentrated under rotavapor. The crude was dissolved in 10 mL CH_2Cl_2 and washed with deionized H_2O (2x 25 mL). The organic phase was dried with Na_2SO_4 , filtered and the solvent was removed with rotavapor. The crude mixture was purified by flash chromatography with1:1 ethyl acetate/Methanol as eluent. The product was obtained as a pale-yellow oil (556 mg, 1.51 mmol, 89% yield).

^{1}H NMR (400 MHz, $CDCl_3$) δ 5.75 (1H, td, *J* = 17.0, 8.5 Hz, 1H), 5.10 (dd, *J* = 17.0, 1.5 Hz, 1H), 5.04 (dd, *J* = 9.9, 1.6 Hz, 1H), 4.14 – 4.06 (set of m, 8H), 3.09 (d, *J* = 7.1 Hz, 2H), 2.91 (td, *J* = 16.2, 6.1 Hz, 2H), 2.49 (tt, *J* = 23.8, 6.1 Hz, 1H), 1.25 (t, *J* = 7.1 Hz, 12H).

^{31}P {^{1}H}- NMR (162 MHz, Chloroform-d) δ 21.58 (s, 2P). ^{1}H {^{31}P}-NMR (400 MHz, Chloroform-*d*) δ 5.83 – 5.68 (set of m, 1H), 5.11 (d, *J* = 15.9 Hz, 1H), 5.06 (d, *J* = 9.9 Hz, 1H), 4.21 – 4.10 (m, 10H), 3.13 (d, *J* = 7.1 Hz, 2H), 2.95 (d, *J* = 6.1 Hz, 3H), 2.52 (s, 0H), 1.30 (t, *J* = 7.1 Hz, 14H).

^{13}C NMR (101 MHz, Chloroform-*d*) δ 134.08, 117.48, 62.87 (d, *J* = 6.6 Hz), 62.72 (d, *J* = 6.7 Hz), 38.85 (t, *J* = 131.6 Hz), 35.81, 26.65 (t, *J* = 4.9 Hz), 16.40 (d, *J* = 6.2 Hz).

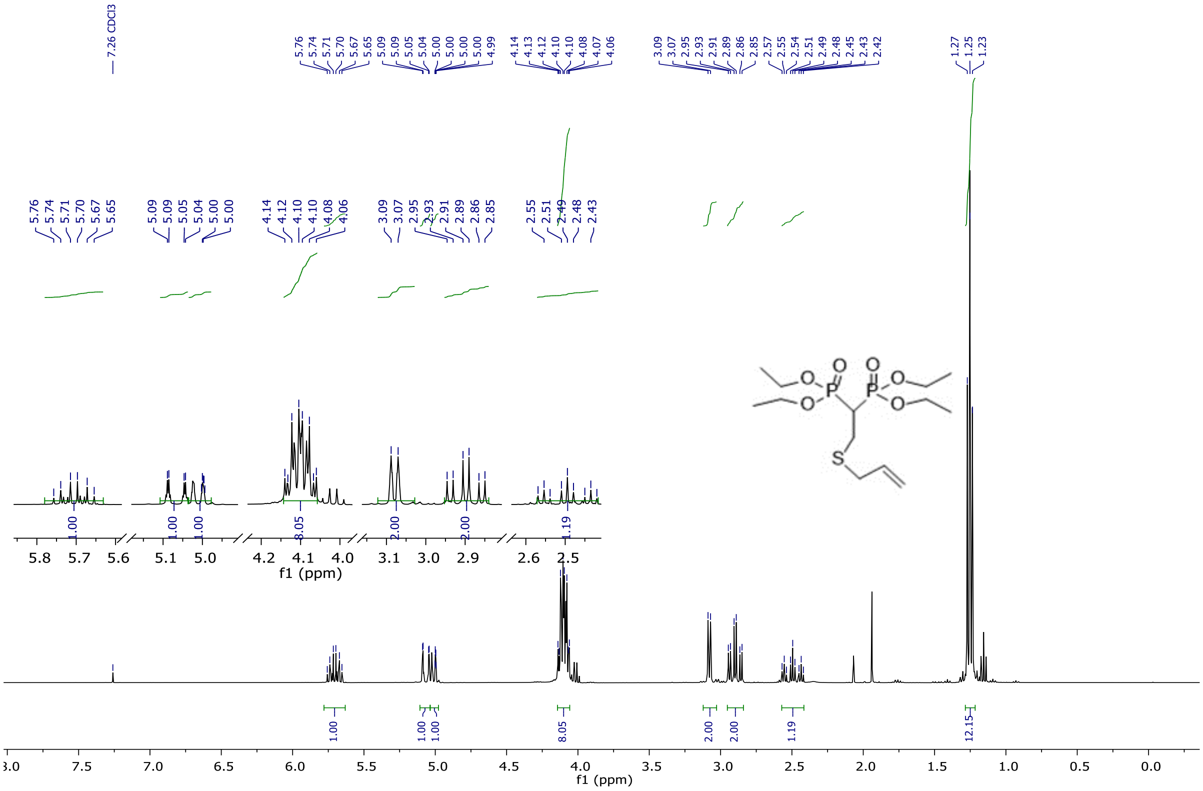

Figure 46. ^{1}H-NMR of **4f**.

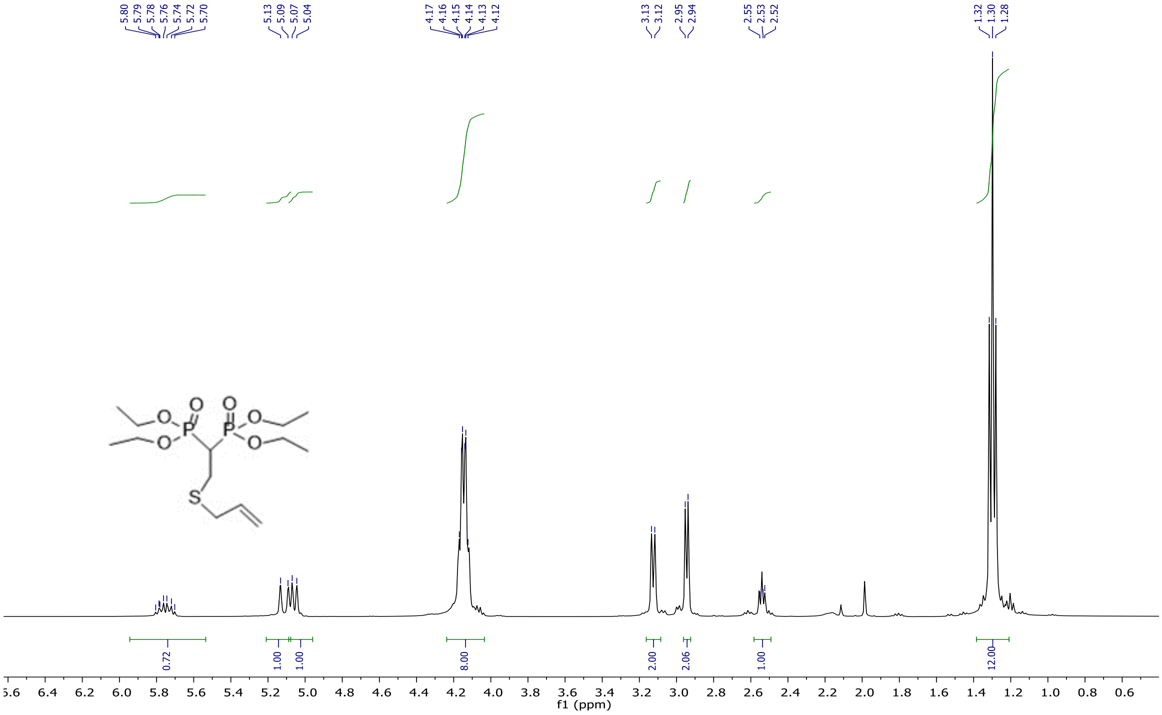

Figure 47. $^{1}H\{^{31}P\}$-NMR of **4f.**

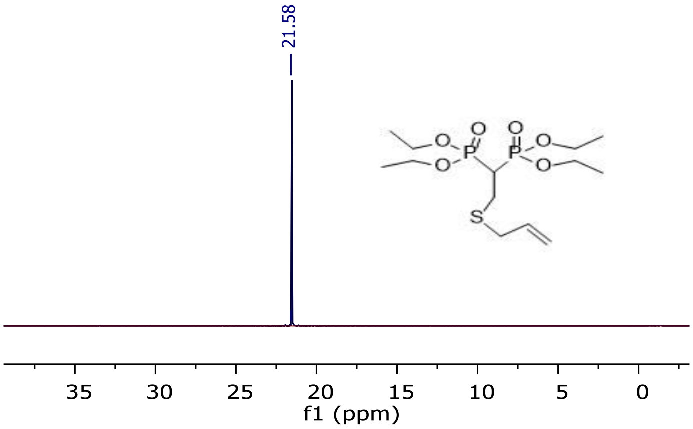

Figure 48. ^{31}P-NMR of **4f**.

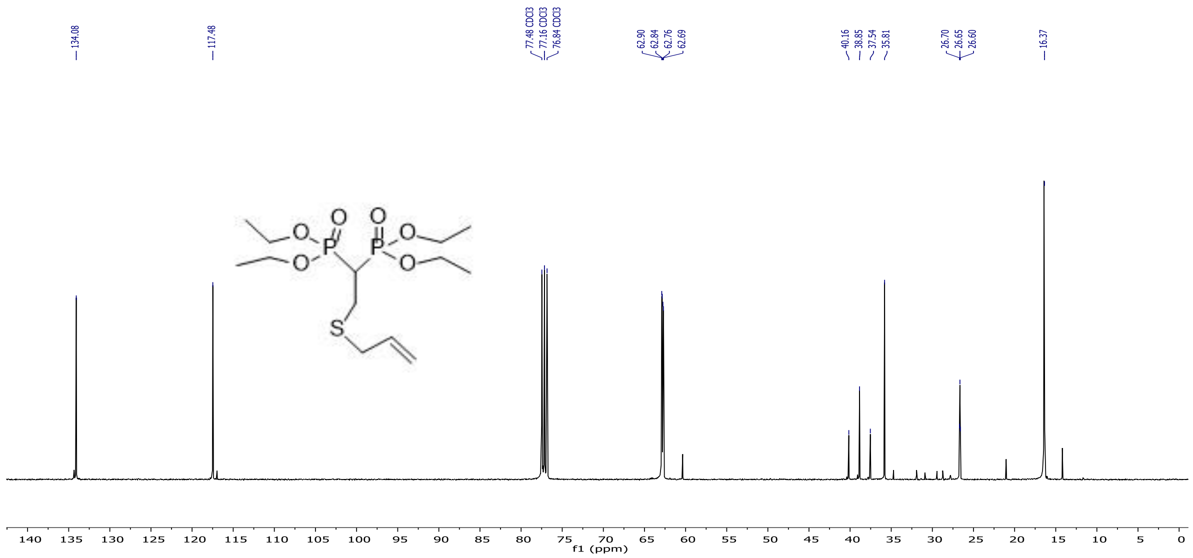

Figure 49. ^{31}C NMR of **4f**.

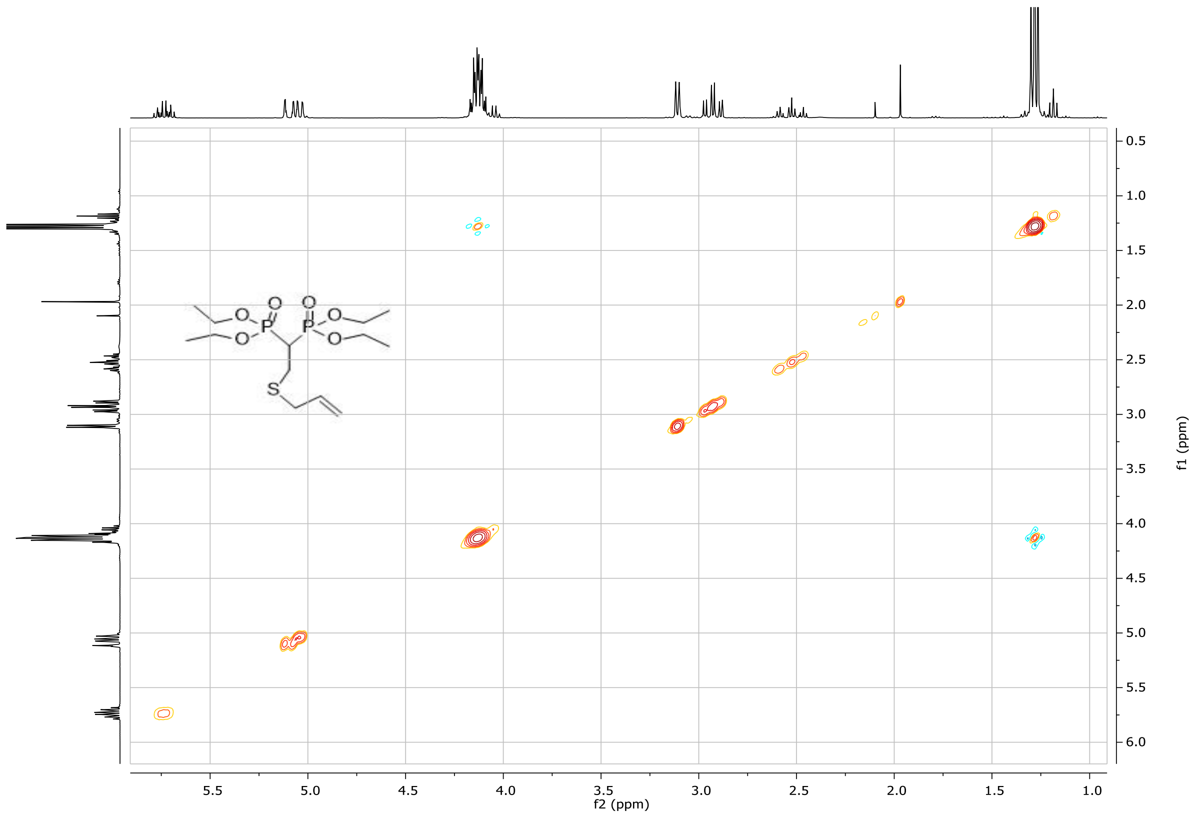

Figure 50. 2D-NMR NOESY of **4f**.

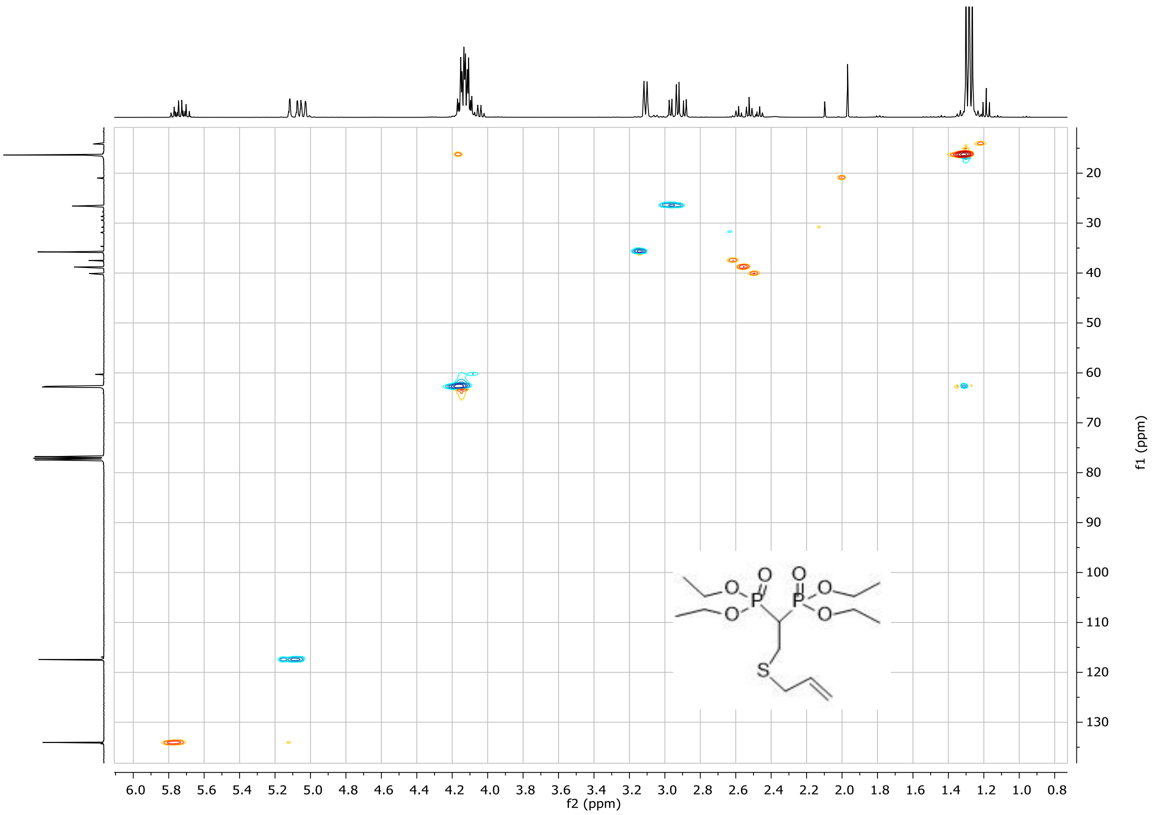

Figure 51. 2D-NMR HSQC of **4f**.

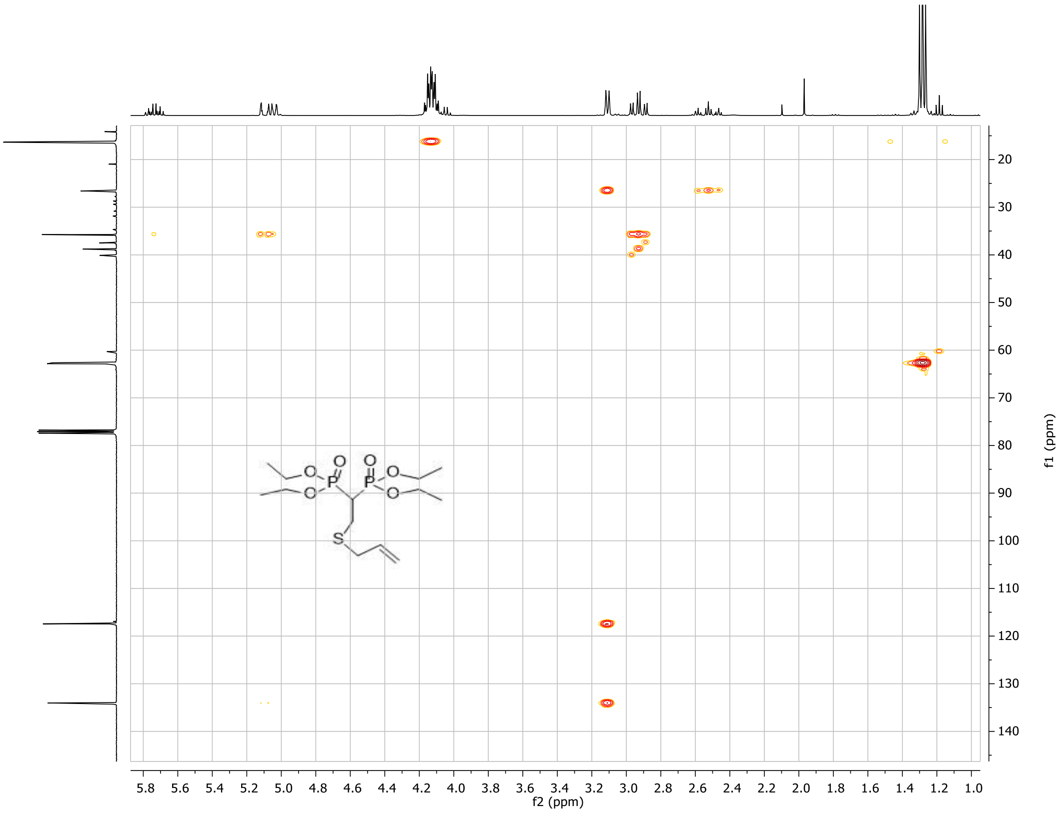

Figure 52. 2D-NMR HMBC of **4f**.

Tetraethyl (2-(pentamethylene dimercaptan) ethane-1,1-diyl)bis(phosphonate) 4g

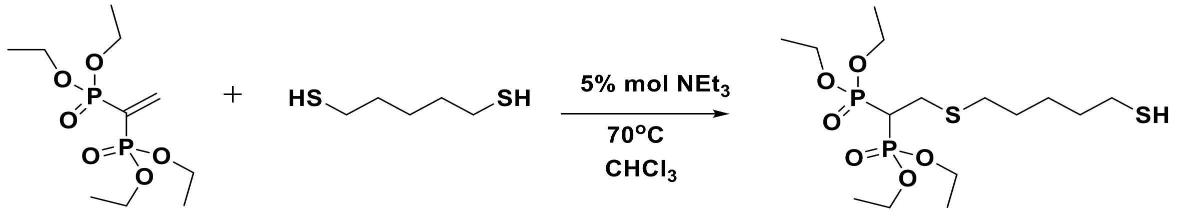

In a 100 mL round bottom flask, vinylidene bisphosphonate tetraethyl ester (VBP, 500 mg, 1.67 mmol) was dispersed in 2.5 mL $CHCl_3$. Then 1,5 pentane dithiol (224 µL, 1.67 mmol) and triethyl amine (12 µL, 5% in mol) were added to the solution. The apparatus was purged with nitrogen and the reaction mixture was refluxed for overnight. The resulting solution was cooled to room temperature and concentrated under rotavapor. The crude was dissolved in 10 mL CH_2Cl_2 and washed with deionized H_2O (2x 25 mL). The organic phase was dried with Na_2SO_4 and precipitate was filtered. Then CH_2Cl_2 solvent was removed with rotavapor. The product was obtained as colorless liquid (704 mg, 1.63 mmol, 98% yield).

^{1}H NMR (400 MHz, Chloroform-*d*) δ 4.21 – 4.11 (m, 8H), 2.99 (td, *J* = 16.3, 5.9 Hz, 2H), 2.55 – 2.45 (m, 5H), 1.63-1.57 (m, 4H), 1.51 – 1.39 (m, 2H), 1.30 (t, *J* = 7.1 Hz, 13H).

^{31}P{^{1}H}-NMR (162 MHz, Chloroform-*d*) δ 21.67 (s, 2P).

^{1}H{^{31}P}-NMR (400 MHz, Chloroform-*d*) δ 4.24 – 4.09 (m, 8H), 2.99 (d, *J* = 5.9 Hz, 2H), 2.58 – 2.42 (m, 5H), 1.61-1.53 (m, 4H), 1.51 – 1.39 (m, 2H), 1.30 (t, *J* = 7.1 Hz, 13H).

^{13}C NMR (101 MHz, Chloroform-*d*) δ 62.9 (d, *J* = 6.6 Hz), 62.8 (d, *J* = 6.7 Hz), 39.1 (t, *J* = 131.5 Hz), 33.6, 33.4, 28.8, 27.8 (t, *J* = 4.9 Hz), 27.5, 24.4, 16.4 (d, *J* = 6.2 Hz).

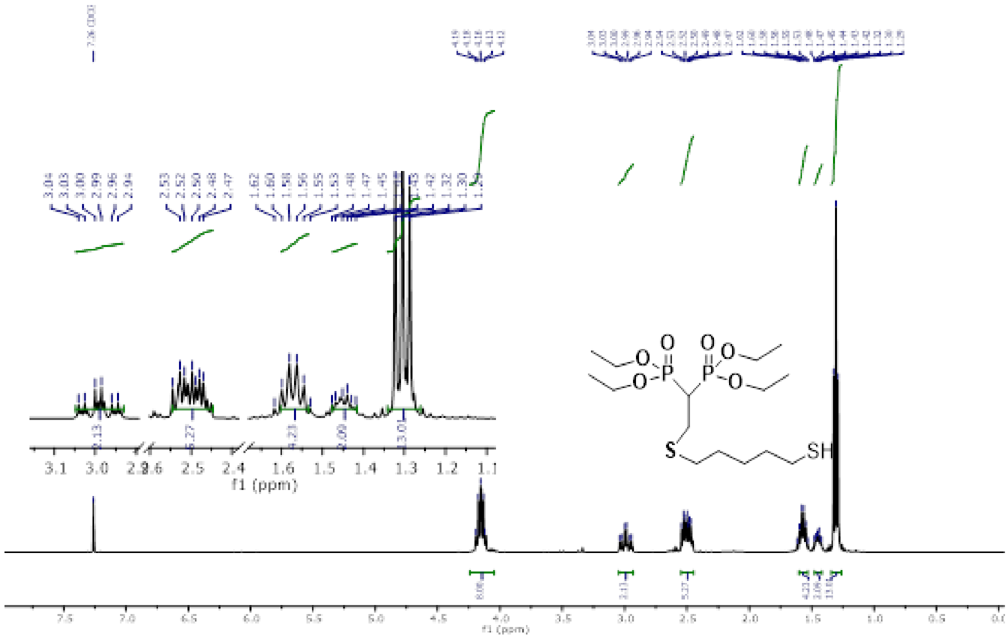

Figure 53. ^{1}H NMR of **4g**.

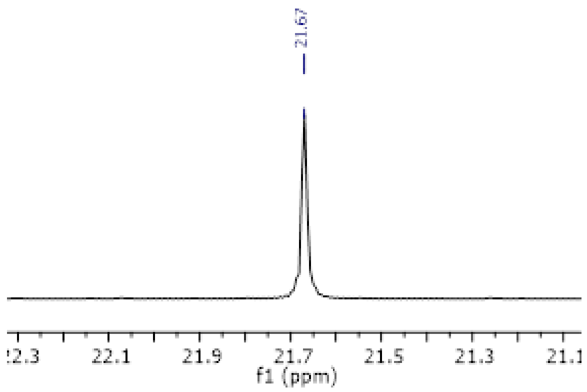

Figure 54. $^{31}P\{^{1}H\}$- NMR of **4g**

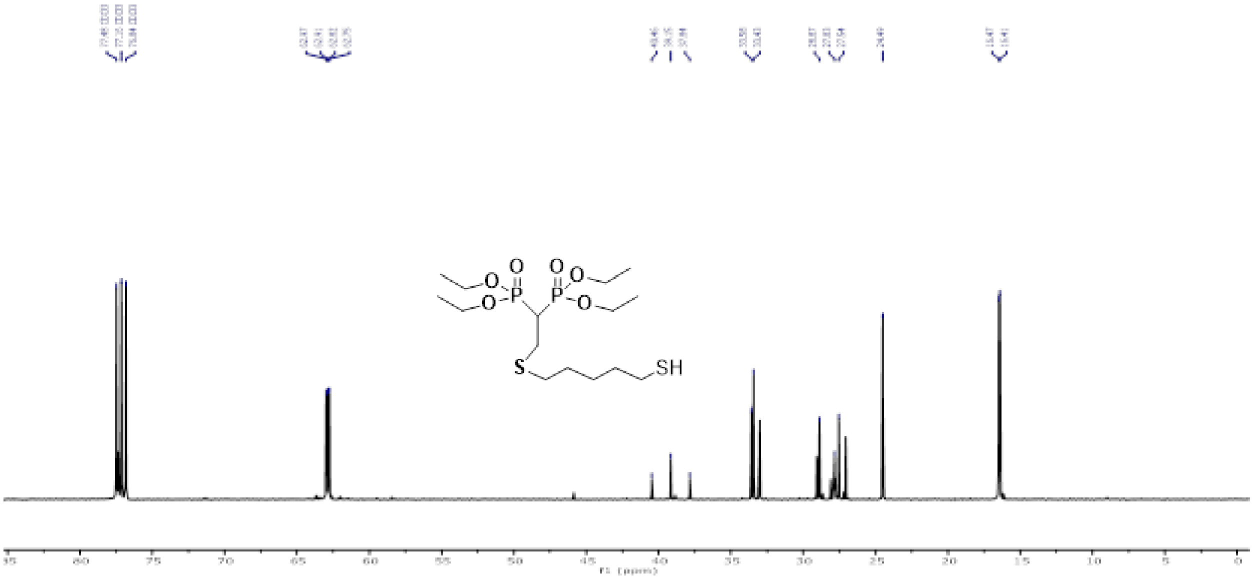

Figure 55. ^{13}C NMR of **4g.**

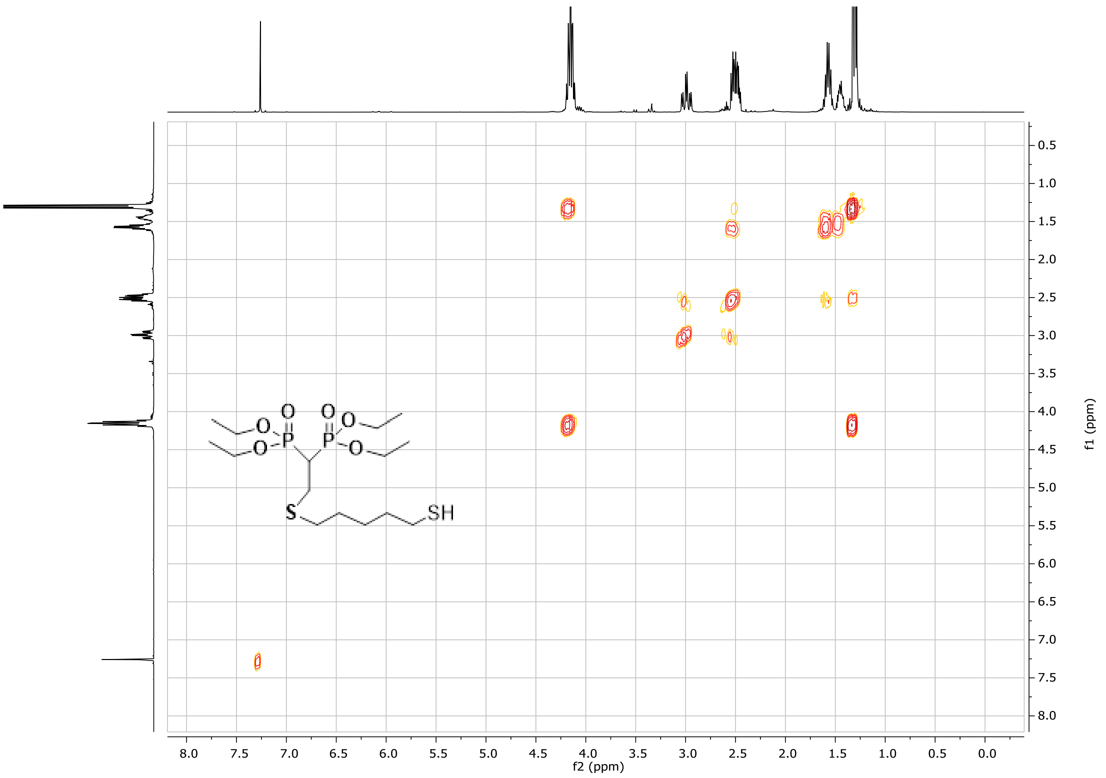

Figure 56. 2D NMR- COSY of **4g**.

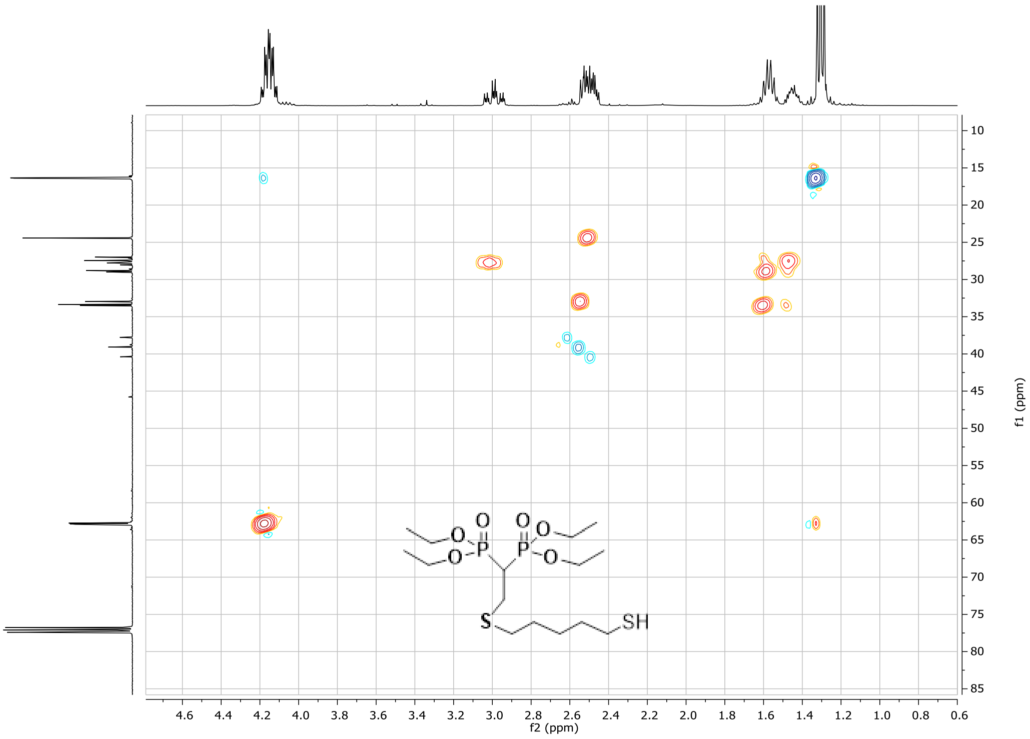

Figure 57. 2D NMR- HSQC of **4g**.

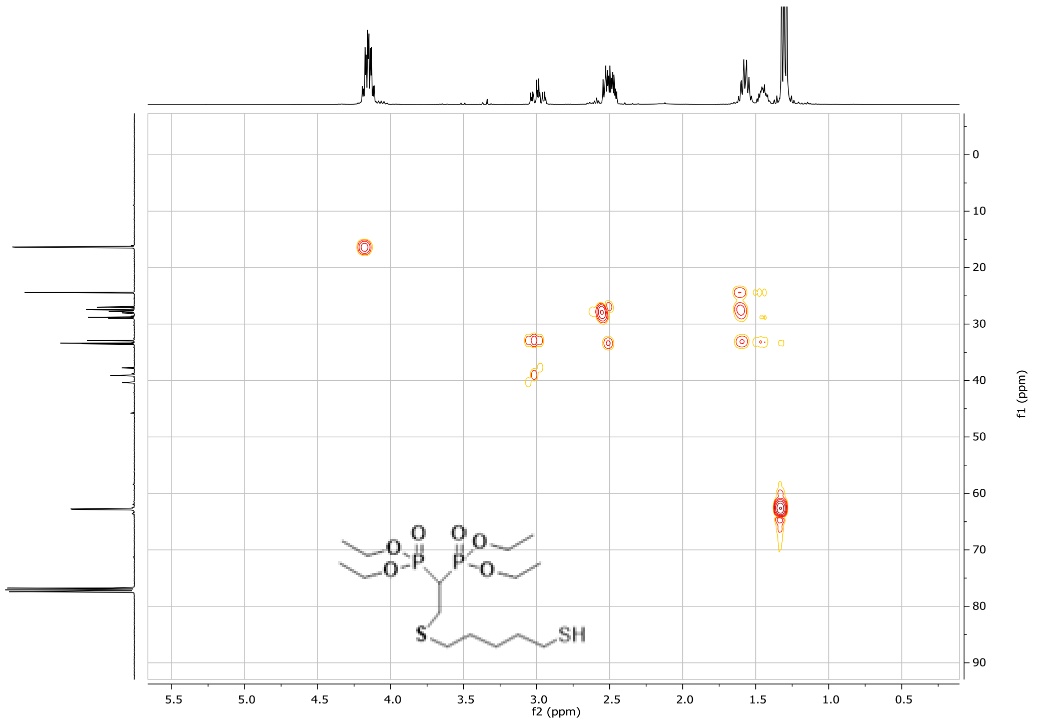

Figure 58. 2D NMR- HMBC of **4g**.

6. Experimental procedures for the synthesis of BP acids 1a-f

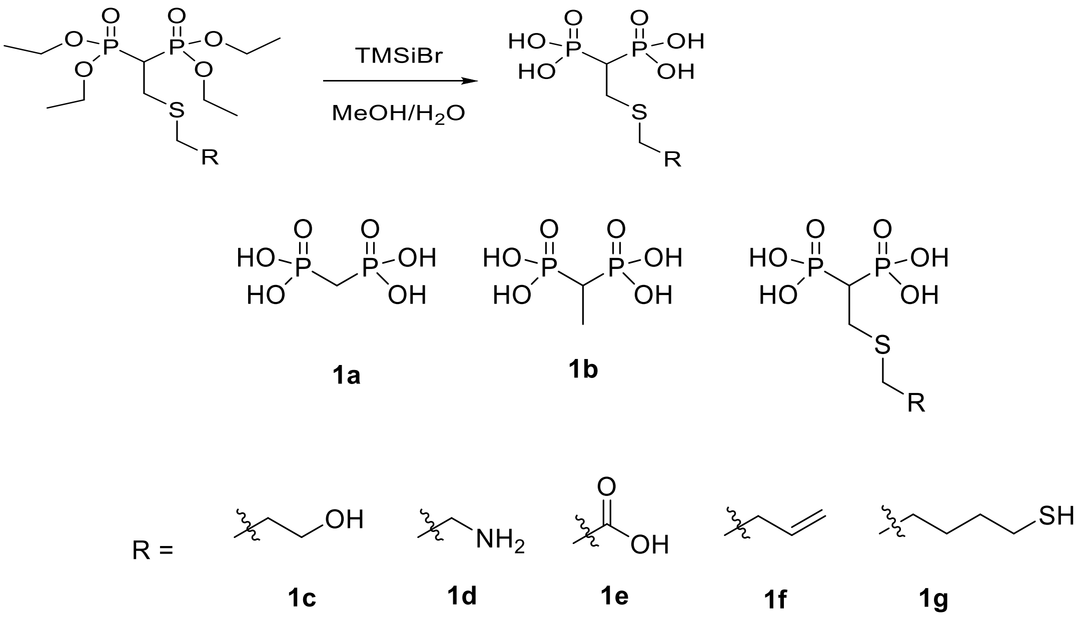

Methylene Bisphosphonic Acid 1a

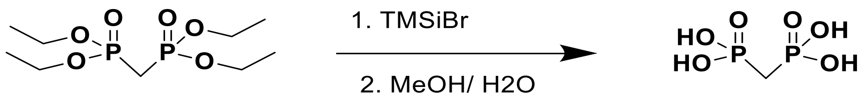

In a 100 mL round bottom flask equipped with magnetic stirring bar, methylene bisphosphonate tetraethyl ester (500 mg, 1.78 mmol) was added in 4-5 mL $C_2H_4Cl_2$ solvent. Then bromo trimethylsilane ($Si(CH_3)_3Br$, 3.46 mL, 26.7 mmol) was added quickly to the solution. The mixture was refluxed under nitrogen atmosphere for an hour and a half. Then the mixture was evaporated under vacuum with a cold trap. A solvent mixture (10 ml MeOH + 1 mL distilled water) was added to the flask and the reaction mixture was left under stirring for 1h. After that, the solvent was remover with rotavapor. The product was obtained as colorless liquid (306 mg, 1.74 mmol, 98% yield).

^{1}H NMR (400 MHz, Deuterium Oxide) δ 2.12 (t, *J* = 20.9 Hz, 2H).

^{31}P{^{1}H} NMR (162 MHz, Deuterium Oxide) δ 18.18 (s, 2P).

^{1}H-{^{31}P} NMR (400 MHz, Deuterium Oxide) δ 2.14 (s, 2H).

^{13}C NMR (101 MHz, Deuterium Oxide) δ 26.59 (t, *J* = 130.2 Hz).

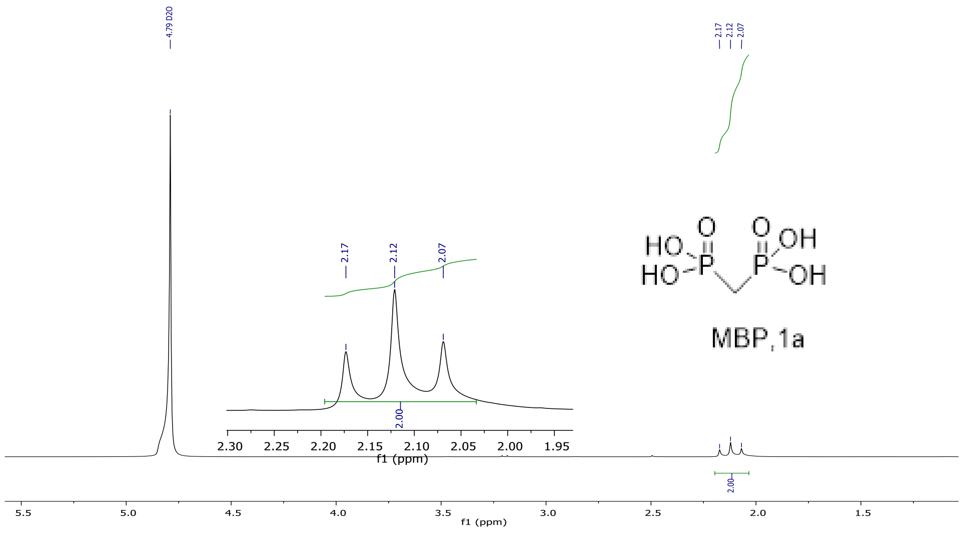

Figure 59. ^{1}H NMR of **1a.**

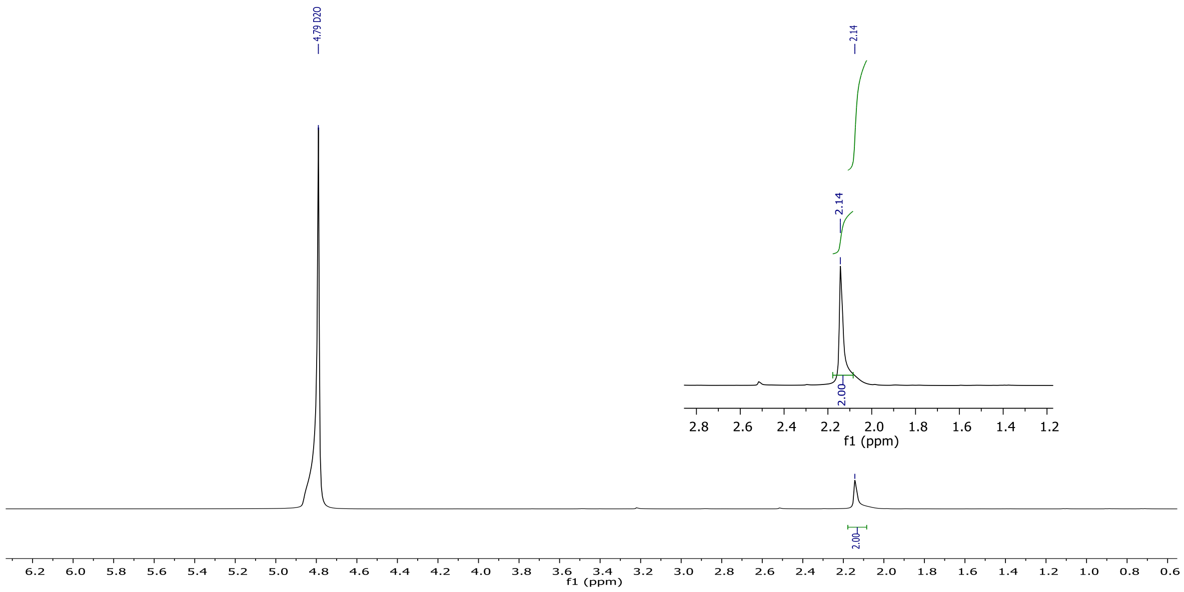

Figure 60. ^{1}H {^{31}P}-NMR of **1a.**

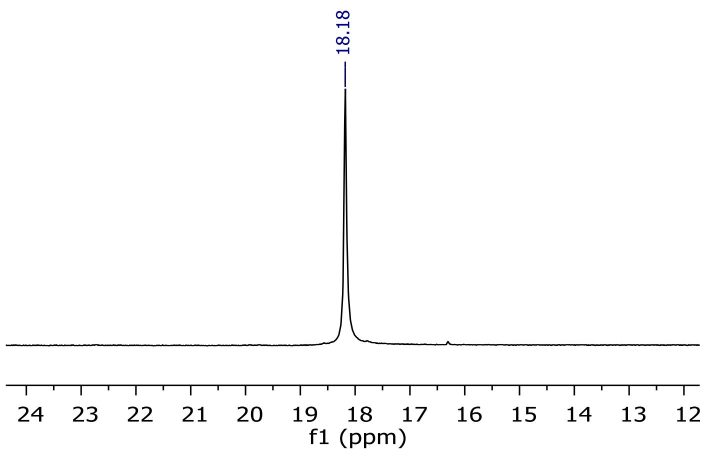

Figure 61. $^{31}P\{^{1}H\}$-NMR of **1a.**

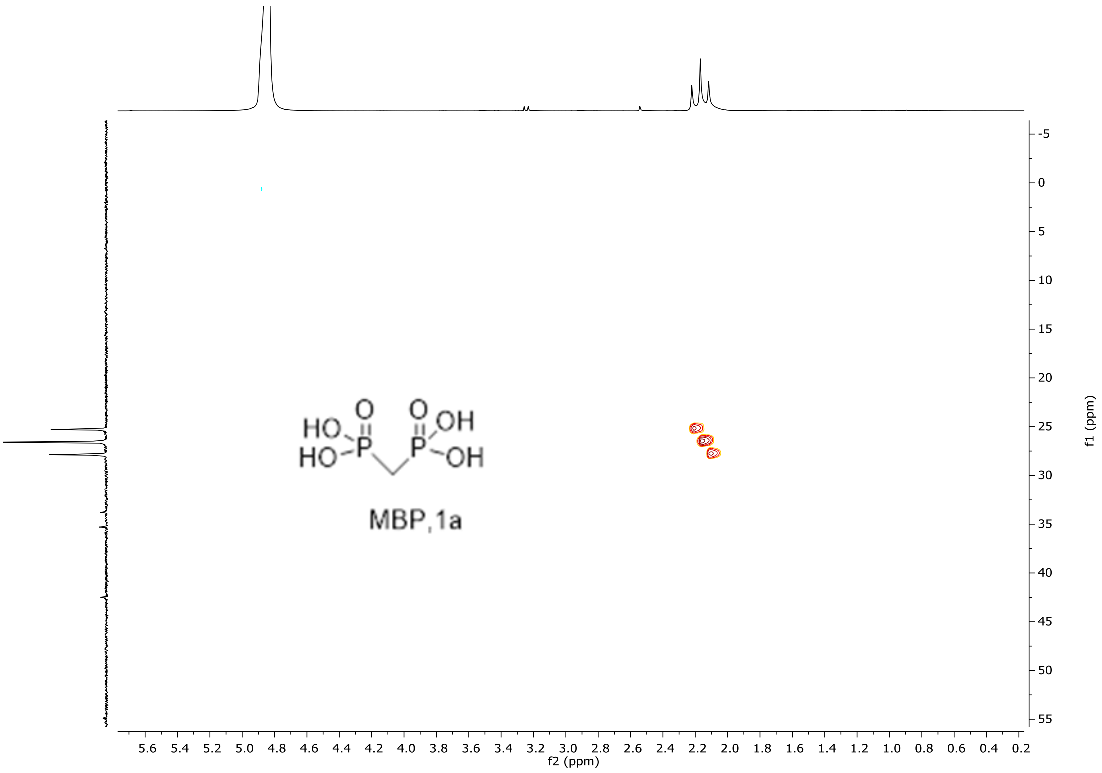

Figure 62. 2D NMR HSQC of **1a.**

Ethane-1,1-diylbis(phosphonic acid) 1b

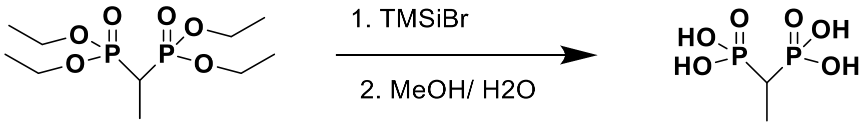

In a 100 mL round bottom flask equipped with a magnetic stirring bar, ethylene bisphosphonate tetraethyl ester (350 mg, 1.76 mmol) was added in 3 mL of dichloromethane. Then bromo trimethylsilane ($Si(CH_3)_3Br$, 2.25 mL, 17.4 mmol) was added quickly to the solution. The mixture was refluxed under nitrogen atmosphere for an hour and a half. Then the mixture was evaporated under vacuum with a cold trap. A solvent mixture (10 ml MeOH + 1 mL distilled water) was added to the flask and the reaction mixture was left stirred for 1h. After that, the solvent was removed with rotavapor. The product was obtained as a colorless liquid (326 mg, 1.72 mmol, 98% yield).

^{1}H NMR (400 MHz, Deuterium Oxide) δ 2.46 – 2.26 (m, 1H), 1.37 (td, *J* = 17.4, 7.4 Hz, 3H).

^{31}P NMR (162 MHz, Deuterium Oxide) δ 22.98 (s).

^{1}H-{^{31}P} NMR (400 MHz, Deuterium Oxide) δ 2.46 – 2.29 (m, 1H), 1.39 (d, *J* = 6.9 Hz, 3H).

^{13}C NMR (101 MHz, Deuterium Oxide) δ 31.71 (t, *J* = 129.3 Hz), 9.37 (t, *J* = 5.5 Hz).

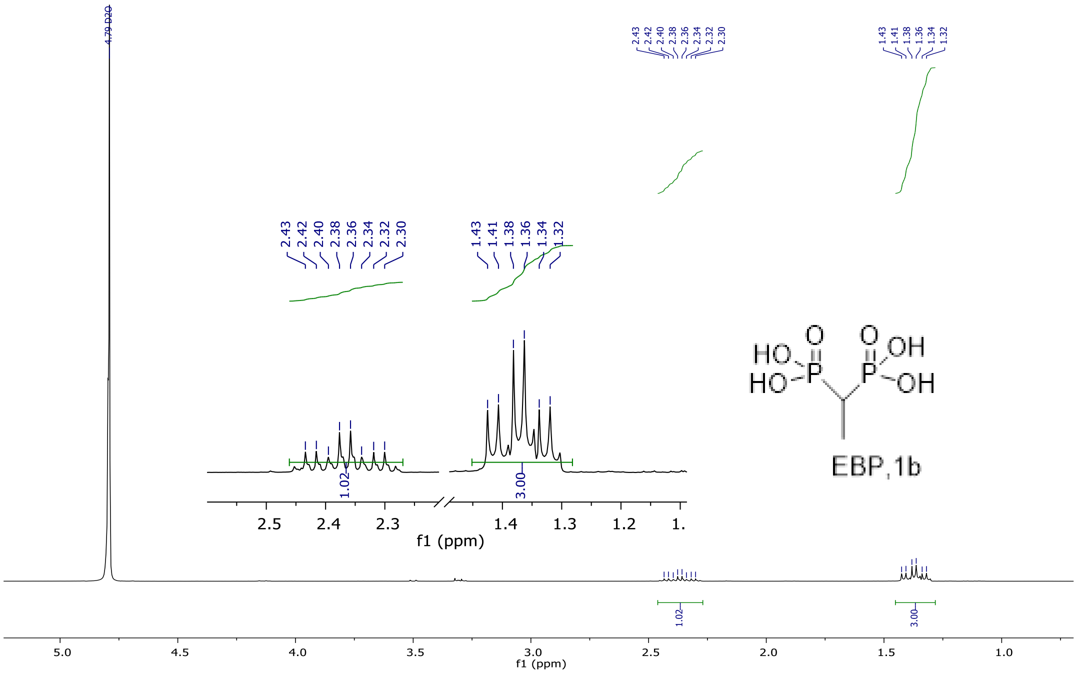

Figure 63. ^{1}H NMR of **1b.**

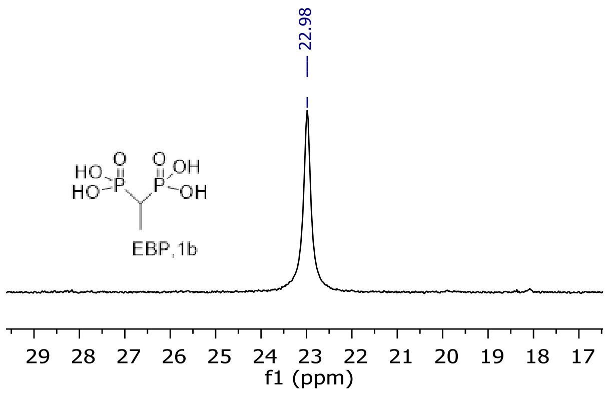

Figure 64. $^{31}P\{^{1}H\}$ NMR of **1b.**

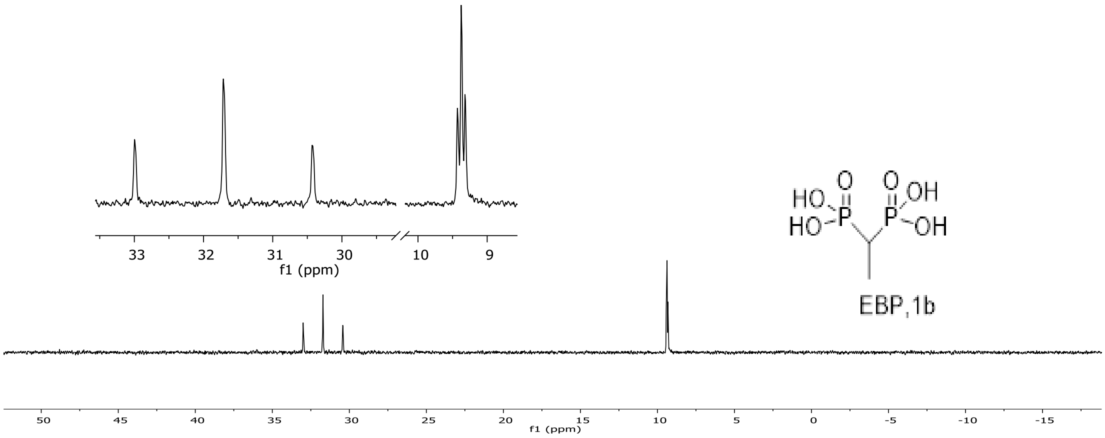

Figure 65. ^{13}C NMR of **1b.**

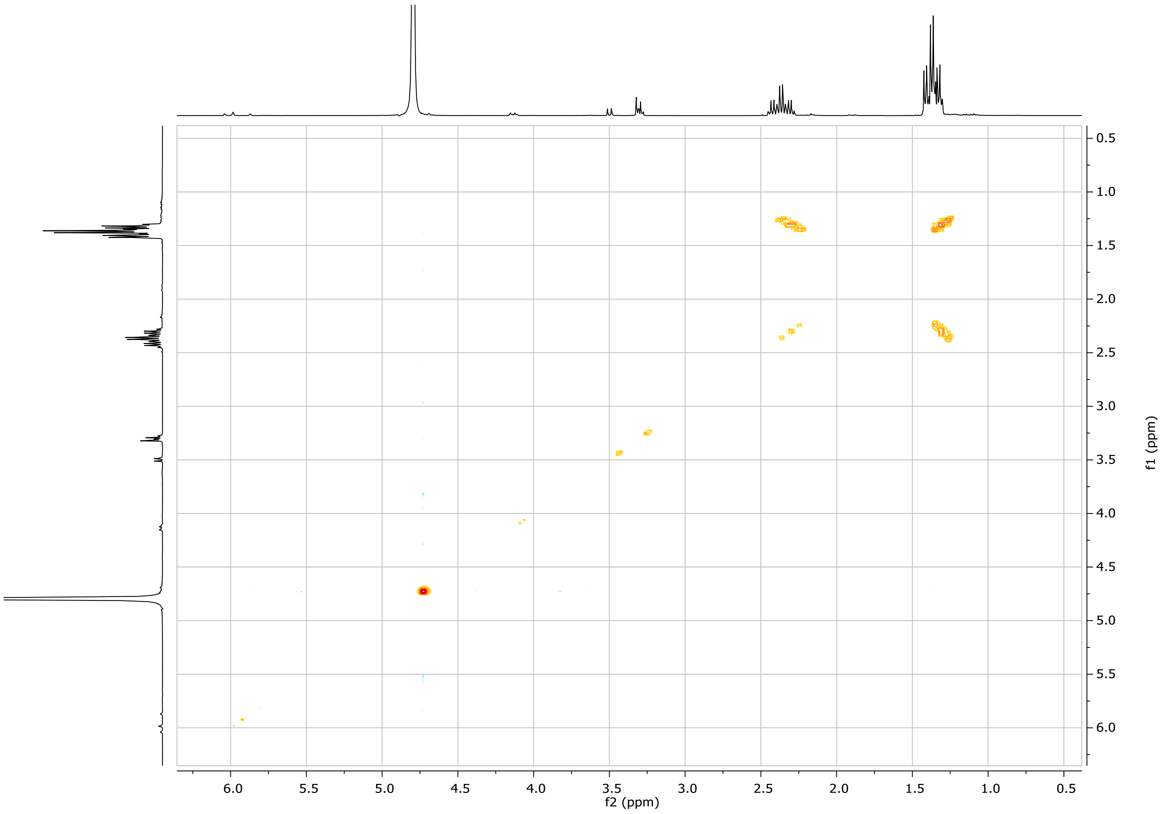

Figure 66. 2D NMR COSY of **1b.**

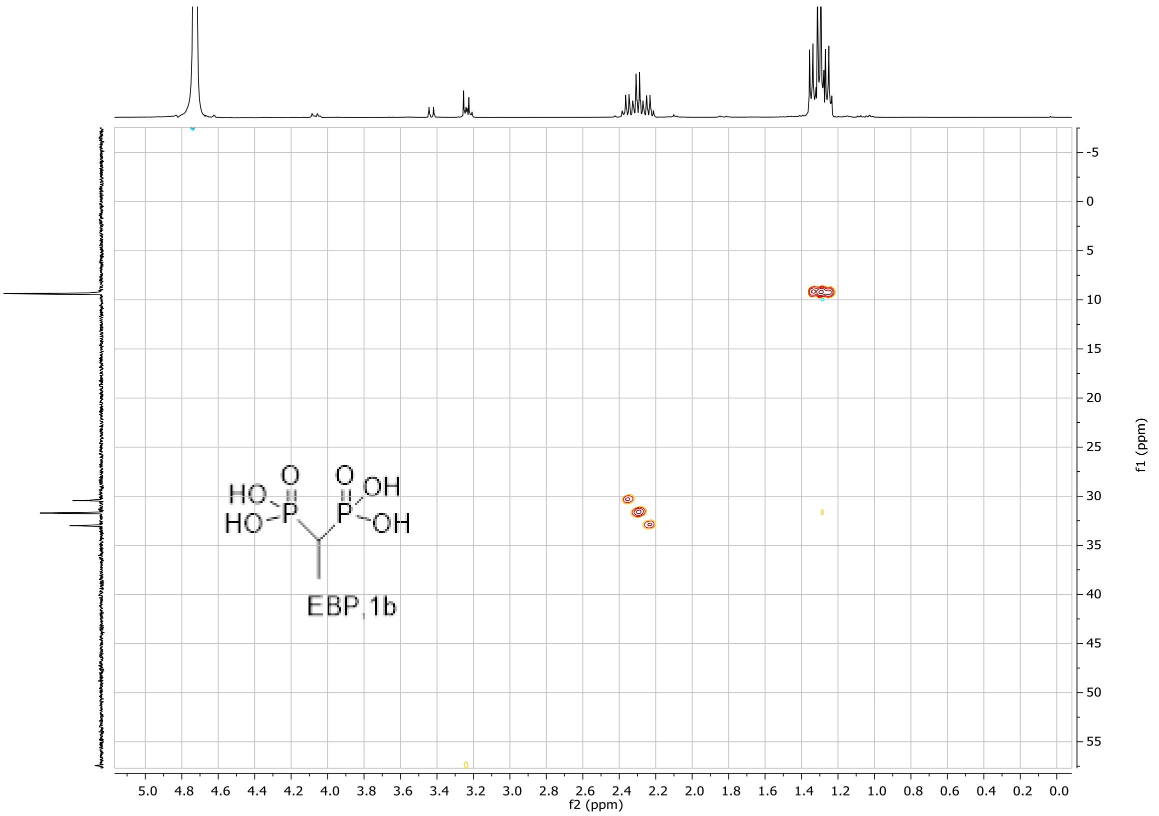

Figure 67. 2D NMR HSQC of **1b.**

(2-((3-hydroxypropyl)thio)ethane-1,1-diyl)bis(phosphonic acid) 1c

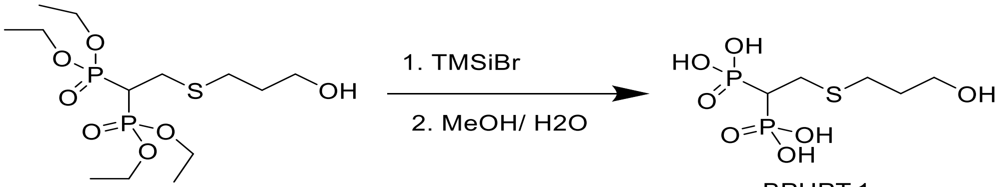

BPHPT,1c

In a 100 mL round bottom flask equipped with a magnetic stirring bar, ethylene bisphosphonate tetraethyl ester (500 mg, 1.28 mmol) was added in 4 mL of dichloromethane. Then bromo trimethylsilane ($Si(CH_3)_3Br$, 2.48 mL, 19.2 mmol) was added quickly to the solution. The mixture was refluxed under nitrogen atmosphere for an hour and a half. Then the mixture was evaporated under vacuum with a cold trap. A solvent mixture (10 ml MeOH + 1 mL distilled water) was added to the flask and the reaction mixture was left stirred for 1h. After that, the solvent was removed with rotavapor. The product was obtained as a colorless liquid (354mg, 1.27 mmol, 99% yield).

^{1}H NMR (400 MHz, Deuterium Oxide) δ 3.66 (t, 2H), 3.04 (td, *J* = 16.0, 6.4 Hz, 2H), 2.68 (t, *J* = 7.3 Hz, 2H), 2.55 (tt, *J* = 23.0, 6.4 Hz, 1H), 1.90 – 1.77 (set of m, 2H).

^{31}P NMR (162 MHz, Deuterium Oxide) δ 19.73 (s,2P).

$^{1}H\{^{31}P\}$-NMR (400 MHz, Deuterium Oxide) δ 3.69 (t, *J* = 6.1 Hz, 2H), 3.08 – 3.02 (d, *J* = 6.1, 2H), 2.69 (t, *J* = 7.1 Hz, 2H), 2.56 (t, *J* = 6.4 Hz, 1H), 1.90 – 1.80 (set of m, 2H).

^{13}C NMR (101 MHz, Deuterium Oxide) δ 60.31 (s), 48.85 (s), 39.11 (t, *J* = 123.7 Hz), 30.87 (s), 28.41 (s), 27.13 (t, *J* = 4.2 Hz).

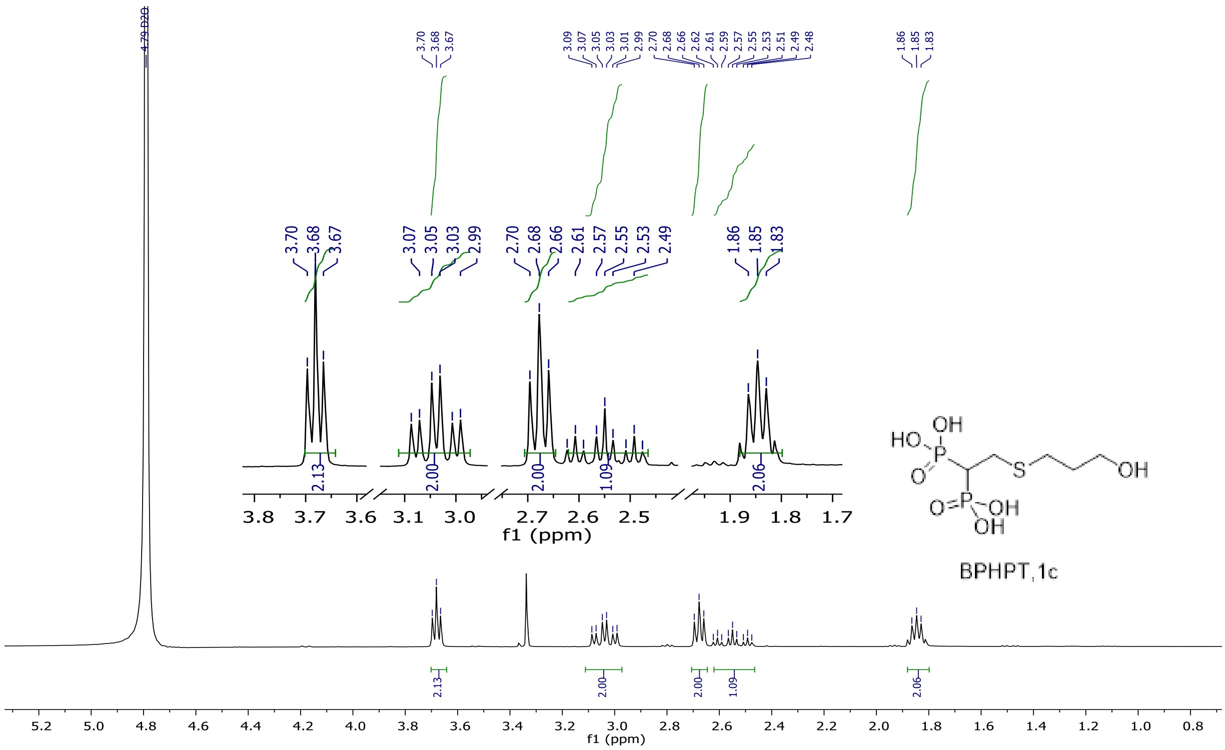

Figure 68. ^{1}H NMR of **1c.**

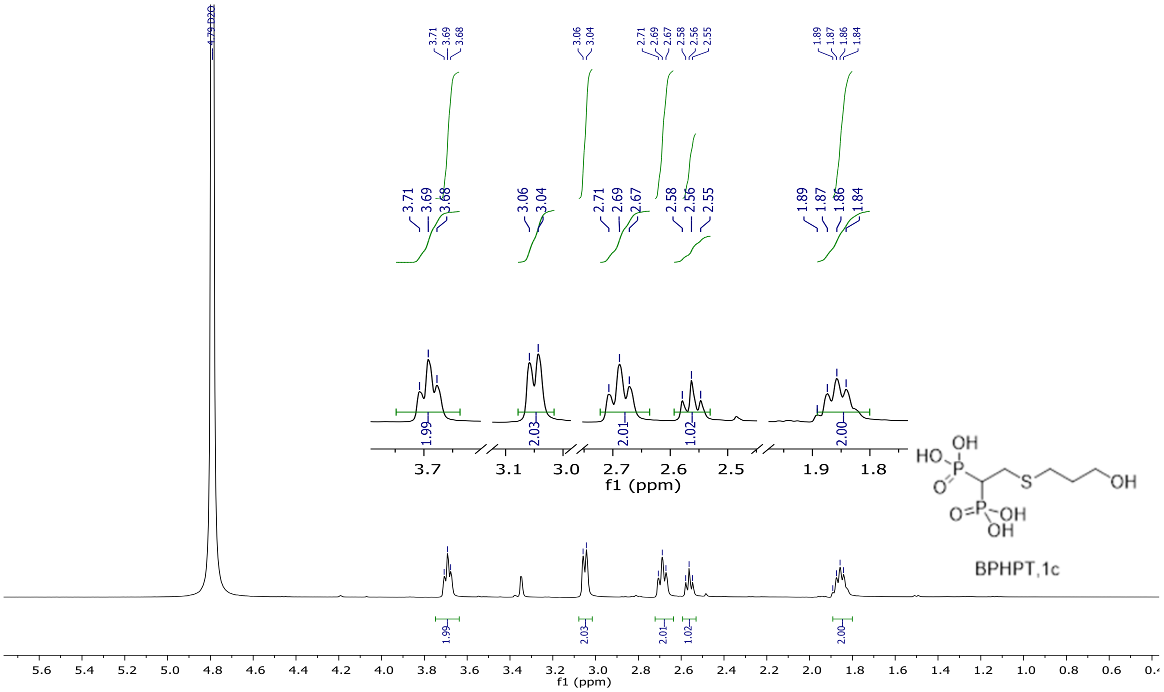

Figure 69. $^{1}H\{^{31}P\}$ NMR of **1c.**

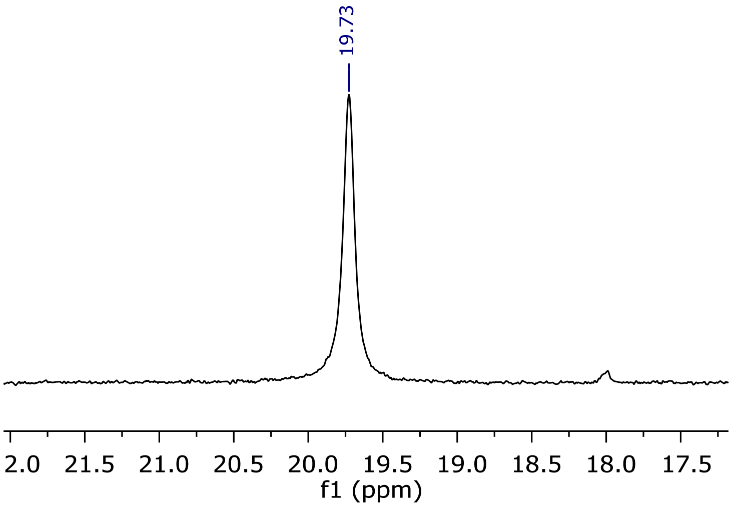

Figure 70. $^{31}P\{^{1}H\}$-NMR of **1c.**

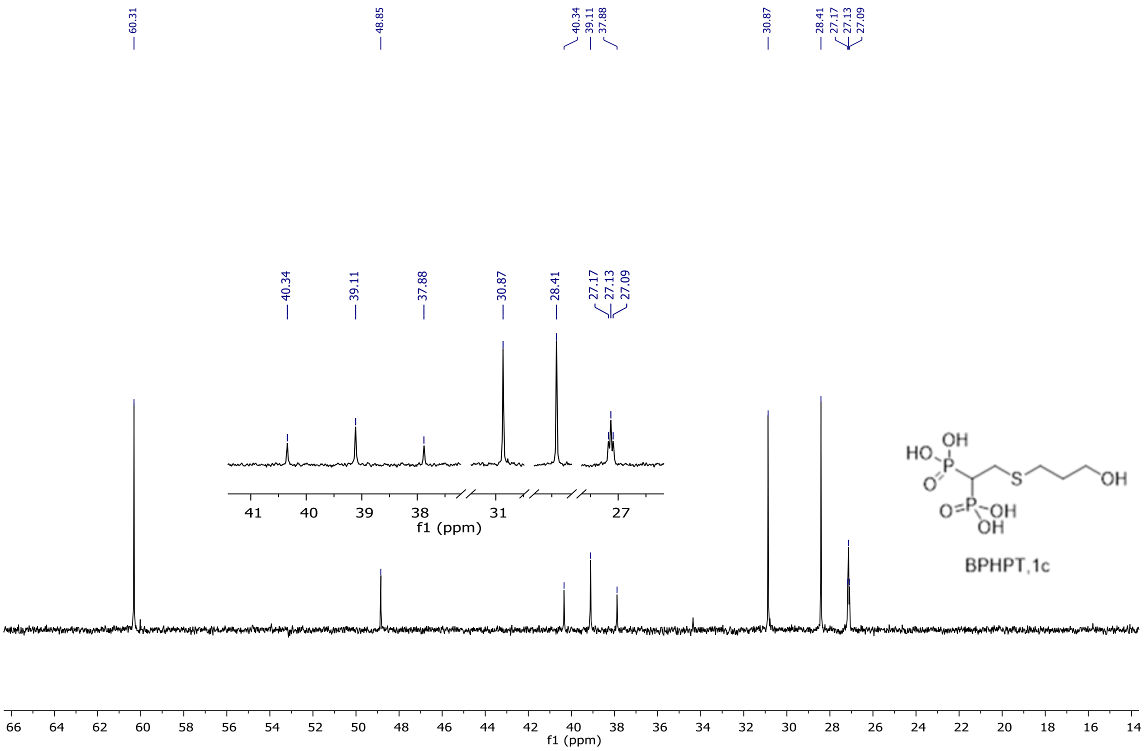

Figure 71. ^{13}C NMR of **1c.**

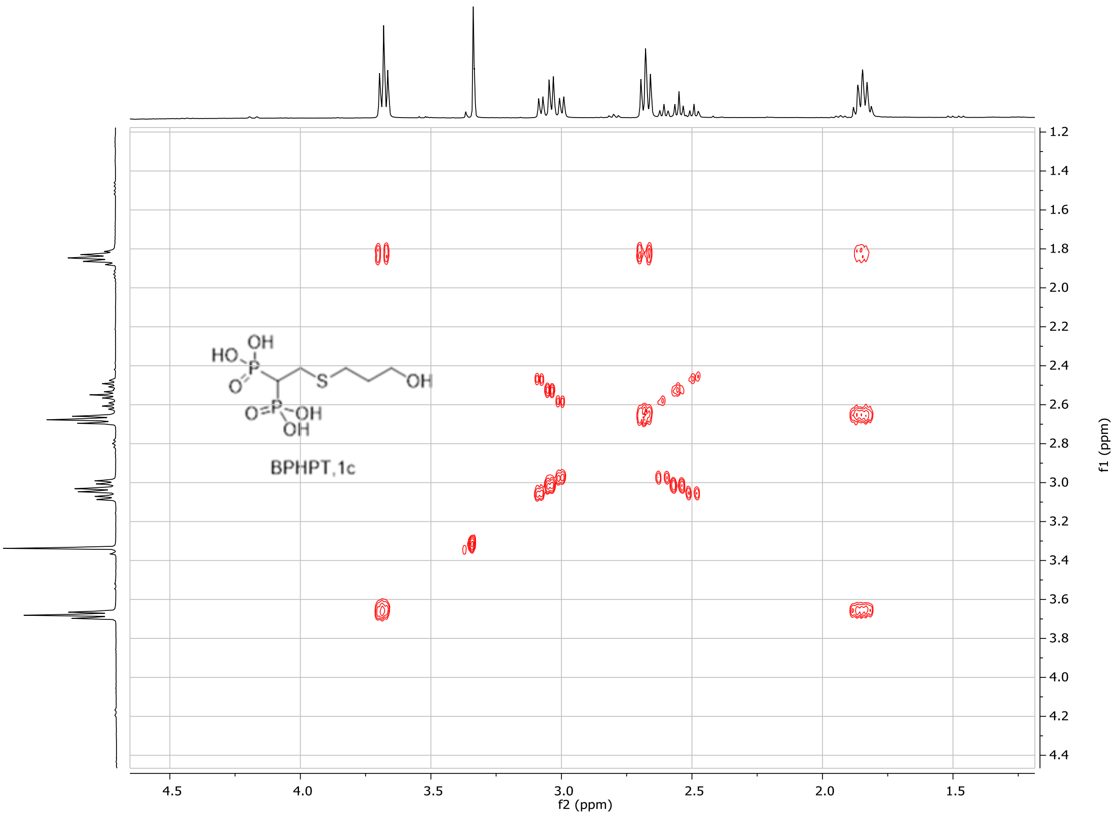

Figure 72. 2D NMR COSY of **1c.**

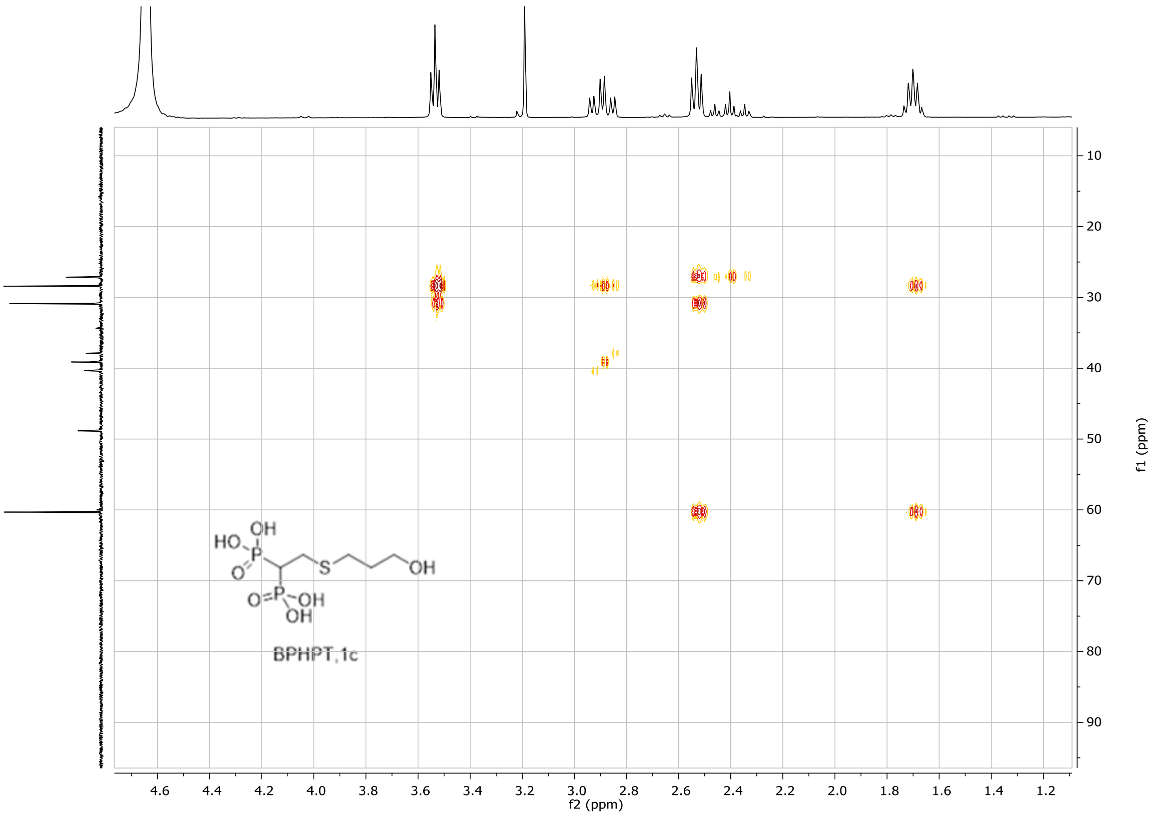

Figure 73. 2D NMR HMBC of **1c.**

(2-((2-aminoethyl)thio)ethane-1,1-diyl)bis(phosphonic acid) 1d

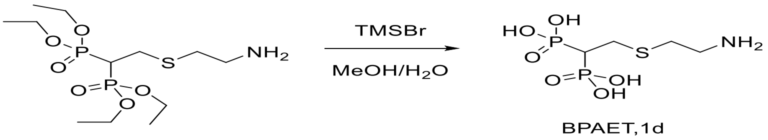

In a 100 mL round bottom flask equipped with a magnetic stirring bar, tetraethyl 2-(amino ethanethiol) ethane-1,1-diyldiphosphonate (500 mg, 1.32 mmol) was added in 4 mL of dichloromethane Then bromo trimethylsilane ($Si(CH_3)_3Br$, 2.57 mL, 19.87 mmol) was added quickly to the solution. The mixture was refluxed under nitrogen atmosphere for an hour and a half. Then the mixture was evaporated under vacuum with a cold trap. A solvent mixture (10 ml MeOH + 1 mL distilled water) was added to the flask and the reaction mixture was left stirred for 1h. After that, the solvent was removed with rotavapor. The product was obtained as a colorless liquid (332 mg, 1.25 mmol, 95% yield).

^{1}H NMR (400 MHz, DMSO-d_6) δ 3.06 – 2.97 (set of m, 2H), 2.92 (td, J = 15.4, 5.8 Hz, 2H), 2.77 (t, J = 6.6 Hz, 2H), 2.34 (tt, J= 22.3, 5.8 Hz, 1H).

^{31}P NMR (162 MHz, DMSO-d_6) δ 18.45 (s, 2P).

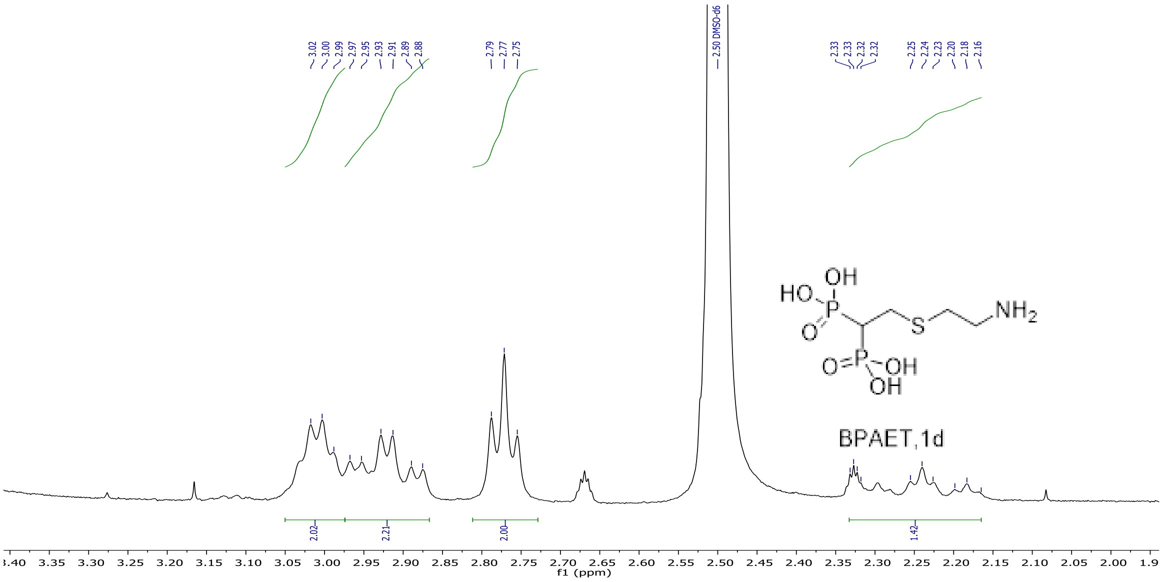

Figure 74. ^{1}H NMR of **1d.**

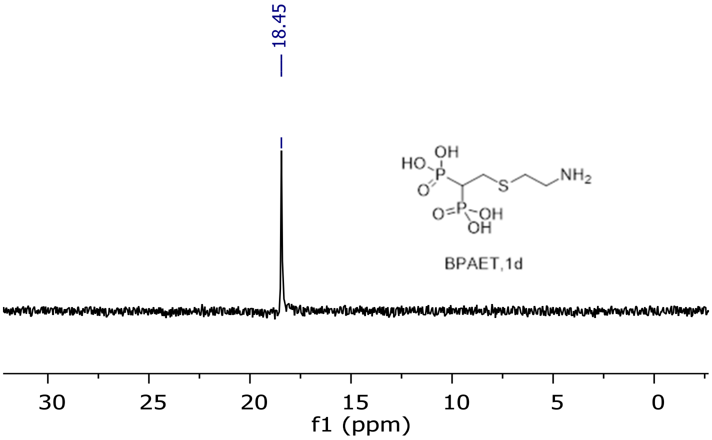

Figure 75. ^{31}P{^{1}H} NMR of **1d.**

2-((2,2-diphosphonoethyl)thio)acetic acid 1e

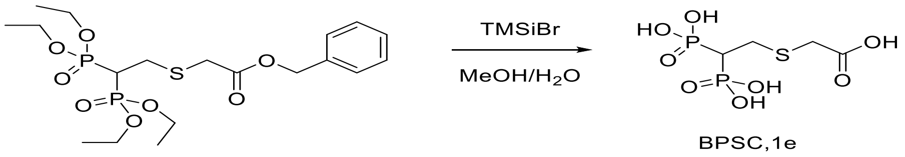

In a 100 mL round bottom flask equipped with a magnetic stirring bar, tetraethyl 2-(benzyl thioglycolate) ethane-1,1-diyldiphosphonate (500 mg, 1.04 mmol) was added in 4 mL $C_2H_4Cl_2$ solvent. Then bromo trimethylsilane ($Si(CH_3)_3Br$, 2.02 mL, 15.60 mmol) was added quickly to the solution. The mixture was refluxed under nitrogen atmosphere for an hour and a half. Then the mixture was evaporated under vacuum with a cold trap. A solvent mixture (10 ml MeOH + 1 mL distilled water) was added to the flask and the reaction mixture was left stirred for 1h. After that, the solvent was removed with rotavapor. The product was obtained as colorless liquid (285 mg, 1.02 mmol, 98% yield).

^{1}H NMR (400 MHz, Deuterium Oxide) δ 3.47 (s, 2H), 3.12 (td, J = 15.9, 6.5 Hz, 2H), 2.61 (tt, J = 22.9, 6.5 Hz, 1H).

^{31}P NMR (162 MHz, Deuterium Oxide) δ 19.22 (s,2P).

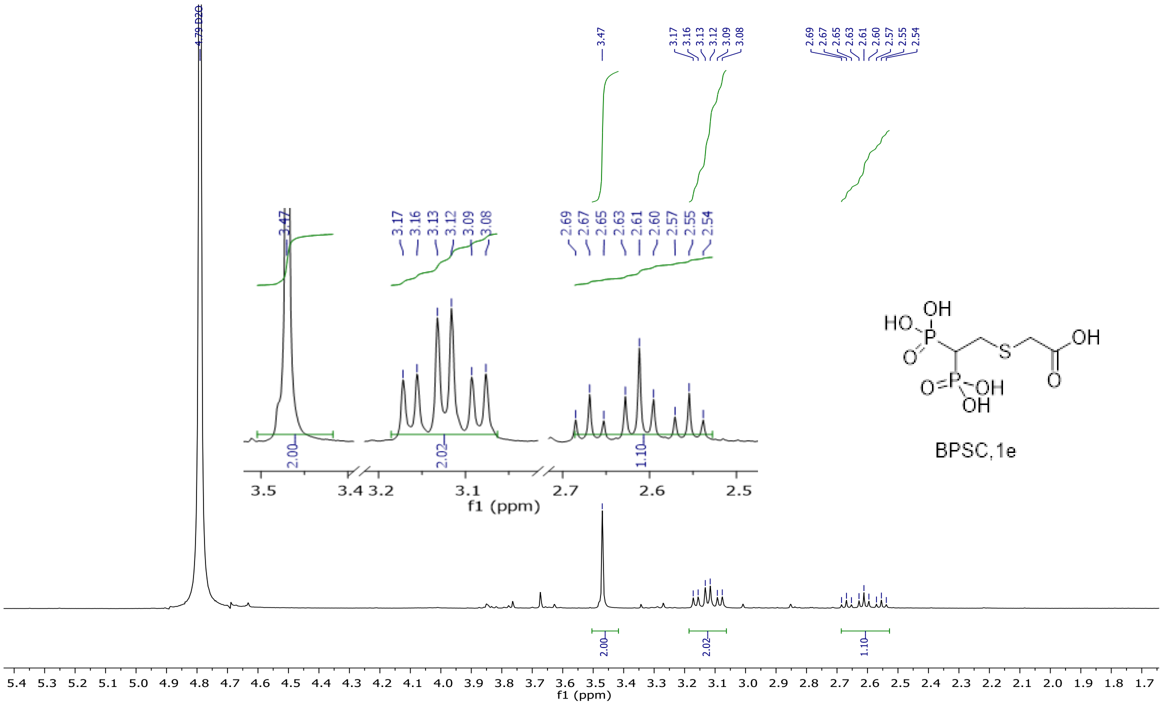

Figure 76. ^{1}H NMR of **1e.**

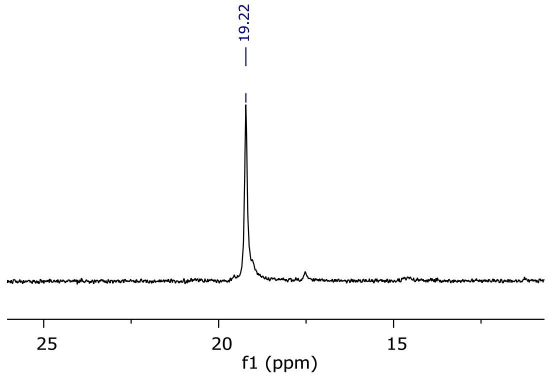

Figure 77. $^{31}P\{^{1}H\}$-NMR of **1e.**

(2-(allylthio)ethane-1,1-diyl)bis(phosphonic acid) 1f

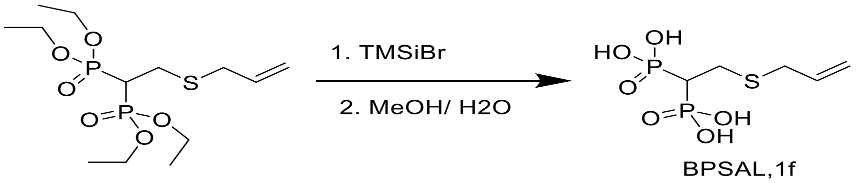

In a 100 mL round bottom flask equipped with a magnetic stirring bar, tetraethyl 2-(2-propene-1- thiol) ethane-1,1-diyldiphosphonate (200 mg, 1.29 mmol) was added in 4 mL of dichloromethane. Then bromo trimethylsilane ($Si(CH_3)_3Br$, 2.50 mL, 19.35 mmol) was added quickly to the solution. The mixture was refluxed under nitrogen atmosphere for an hour and a half. Then the mixture was evaporated under vacuum with a cold trap. A solvent mixture (10 ml MeOH + 1 mL distilled water) was added to the flask and the reaction mixture was left stirred for 1h. After that, the solvent was removed with rotavapor. The product was obtained as colorless liquid (324 mg, 1.24 mmol, 96% yield).

^{1}H NMR (400 MHz, Deuterium Oxide) δ 5.80 (td, *J* = 17.2, 7.2 Hz, 1H), 5.19 (q, *J* = 1.2 Hz, 1H), 5.12 (d, *J* = 11.6 Hz, 1H), 3.19 (d, *J* = 7.2 Hz, 2H), 2.94 (td, *J* = 15.9, 6.6 Hz, 2H), 2.55 (tt, *J* = 23.1, 6.6 Hz, 1H).

^{31}P{^{1}H}-NMR (162 MHz, Deuterium Oxide) δ 19.84 (s).

^{1}H{^{31}P} NMR (400 MHz, Deuterium Oxide) δ 5.81 (dt, *J* = 17.1, 7.4 Hz, 1H), 5.20 (s, 1H), 5.14 (d, *J* = 10.9 Hz, 1H), 3.20 (d, *J* = 7.2 Hz, 2H), 2.95 (d, *J* = 6.5 Hz, 2H), 2.57 (t, *J* = 6.5 Hz, 1H).

^{13}C NMR (101 MHz, Deuterium Oxide) δ 133.73 (s), 117.84 (s), 38.80 (t, *J* = 125.2 Hz), 34.59 (s), 25.94 (t, *J* = 4.2 Hz).

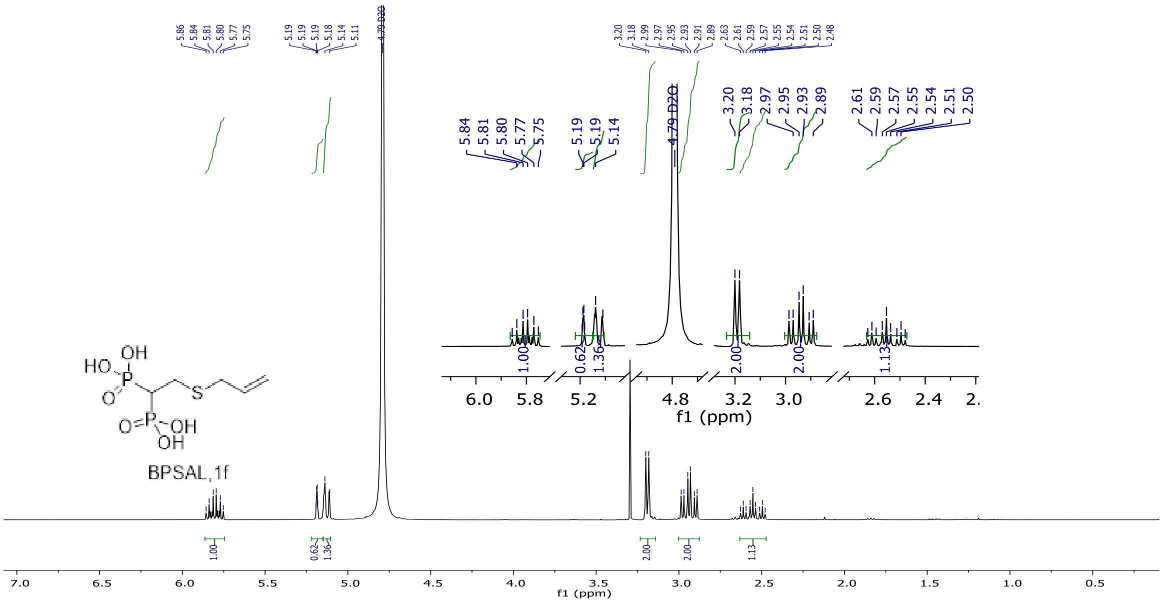

Figure 78. ^{1}H NMR of **1f.**

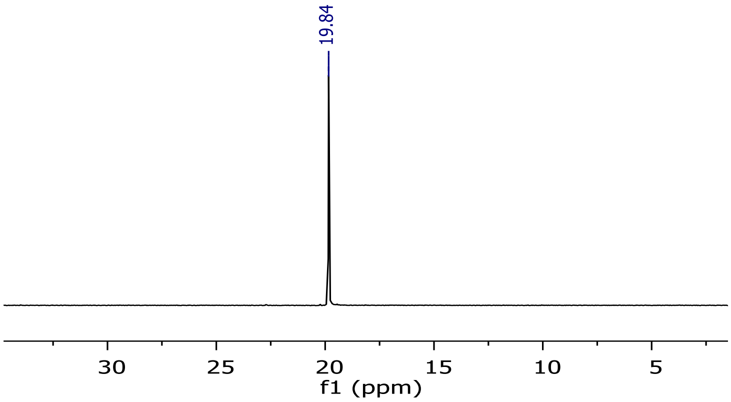

Figure 79. ^{31}P NMR of **1f.**

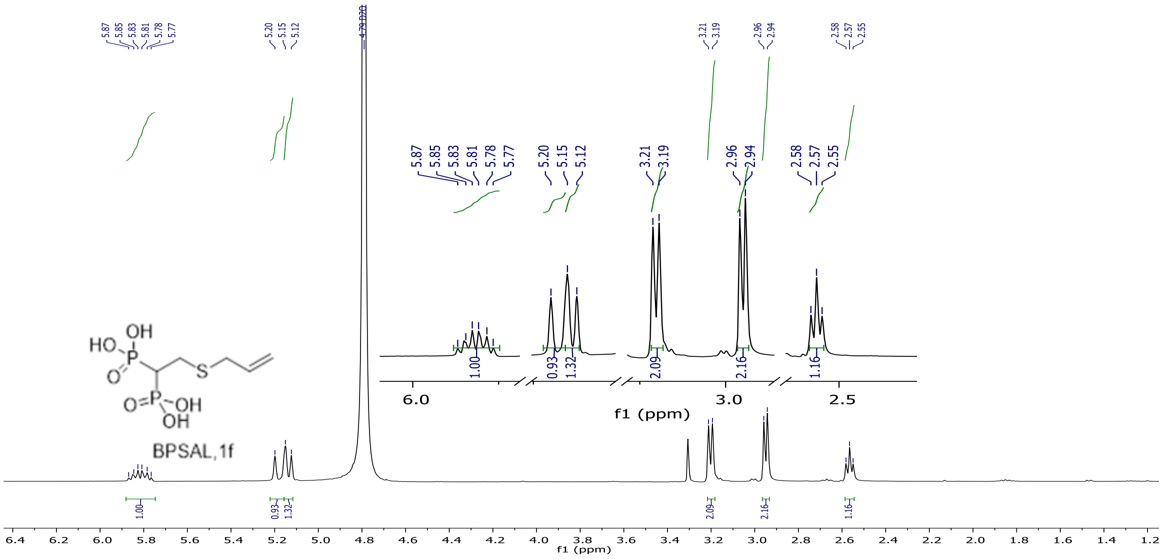

Figure 80. ^{1}H{^{31}P}- NMR of **1f.**

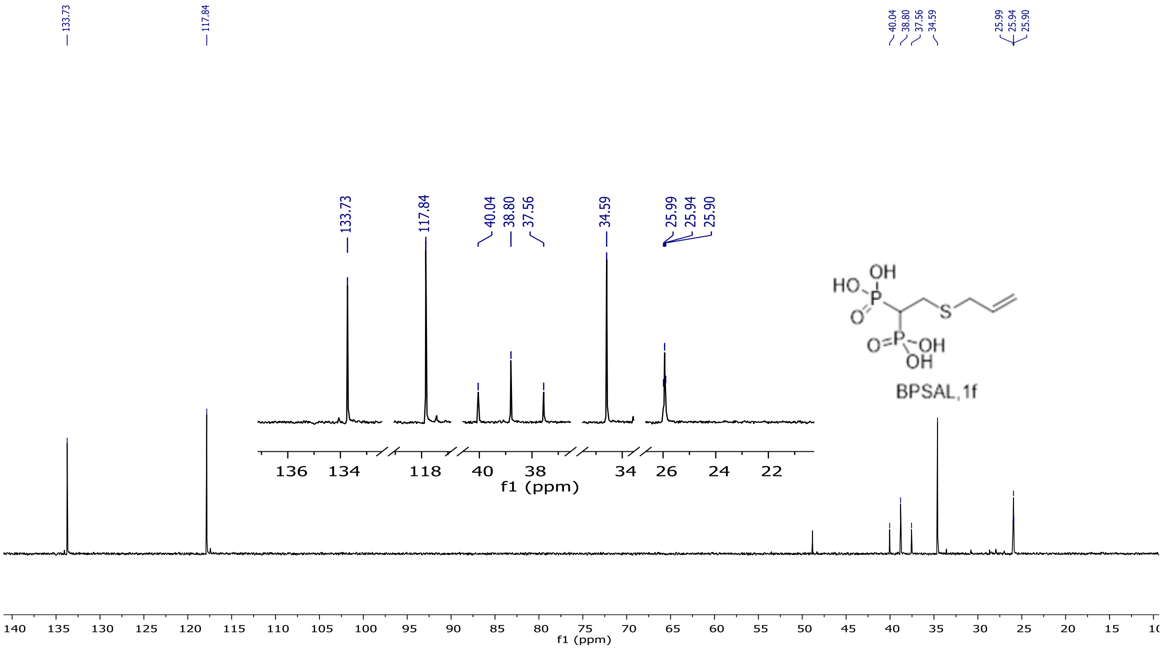

Figure 81. ^{13}C NMR of **1f.**

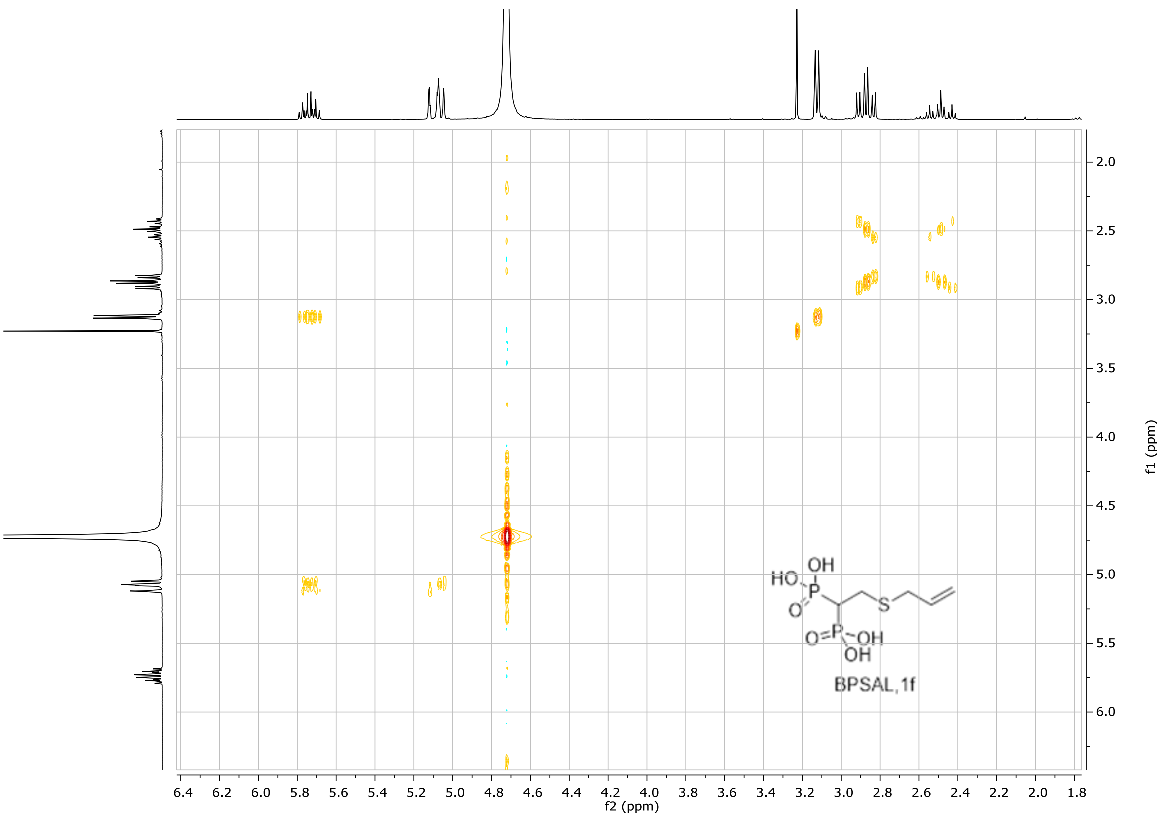

Figure 82. 2D NMR COSY of **1f.**

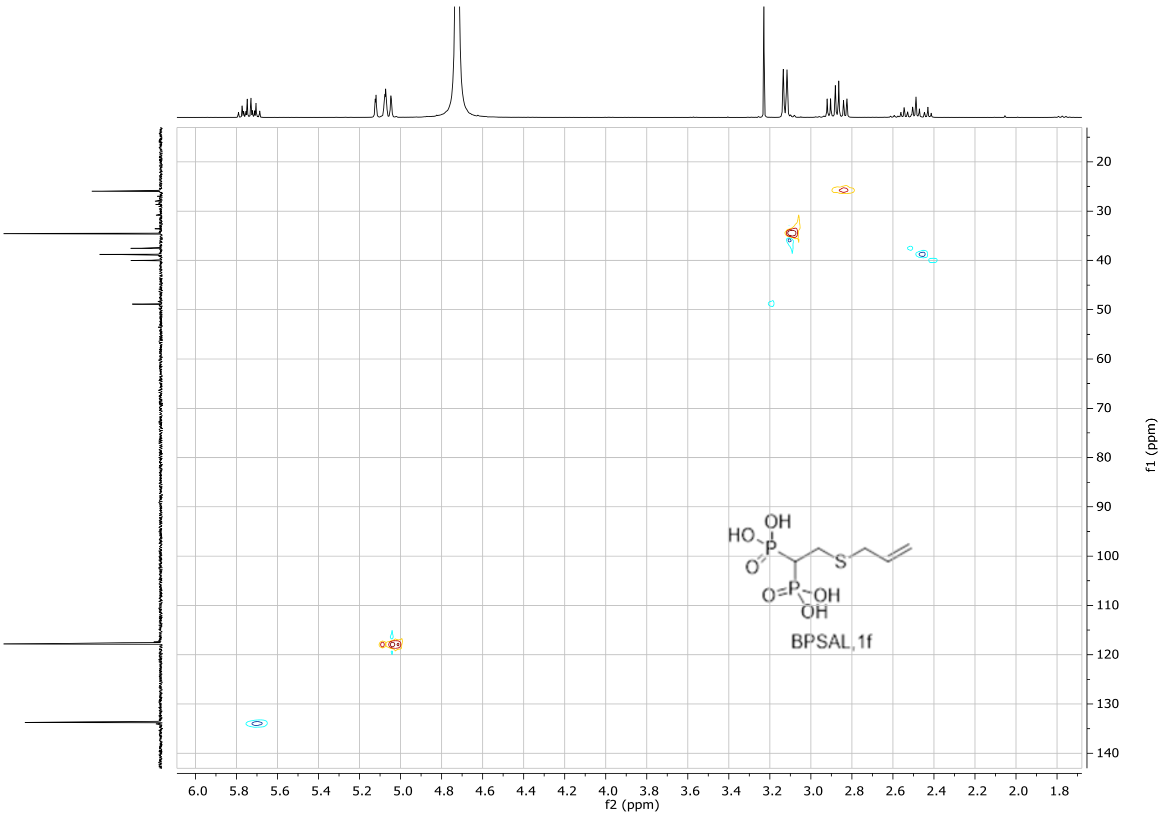

Figure 83. 2D NMR HSQC of **1f.**

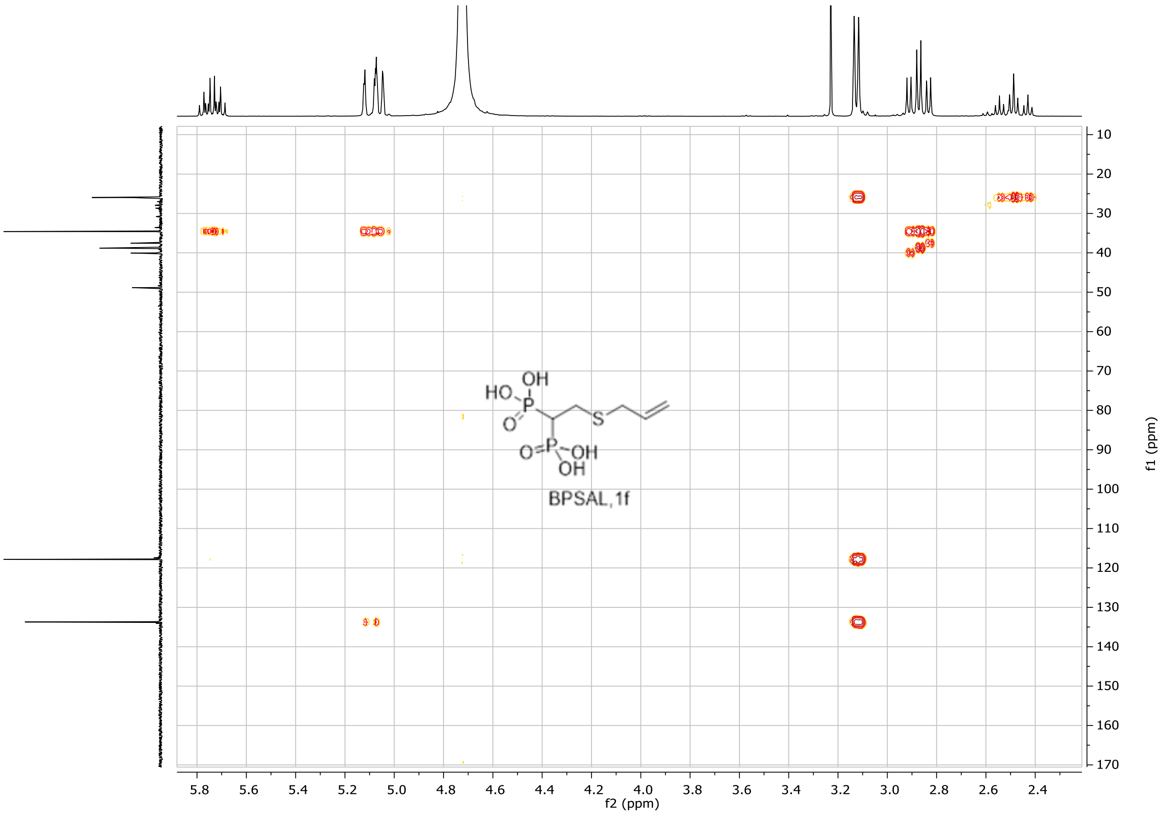

Figure 84. 2D NMR HMBC of **1f.**

2- (pentamethylene dimercaptan)ethane-1,1- diyl)bis(phosphonic Acid) 1g

In a 100 mL round bottom flask equipped with magnetic stirring bar tetraethyl 2-(pentamethylene dimercaptan) ethane-1,1-diyldiphosphonate (500 mg, 1.15 mmol) was added in 4 mL $C_2H_4Cl_2$ solvent. Then bromotrimethylsilane ($Si(CH_3)_3Br$, 2.23 mL, 17.25 mmol) was added quickly to the solution. The mixture was refluxed under nitrogen atmosphere for an hour and a half. Then the flask was putted into high vacuum with N_2 for 1 h. Then vacuum was removed and solvent mixture (10 ml MeOH + 1 mL distilled water) was added to the flask and the reaction mixture was left stirred for 1h. After that, the solvent was remover with rotavapor. The product **1g** was obtained as colorless liquid (358 mg, 1.10 mmol, 96% yield).

^{1}H NMR (400 MHz, Deuterium Oxide) δ 2.99 (td, J = 16.1, 6.4 Hz, 2H), 2.63 – 2.56 (m, 4H), 2.57 – 2.43 (m, 1H), 1.58 (m, 4H), 1.45 (m, 2H).

^{31}P{^{1}H} NMR (162 MHz, Deuterium Oxide) δ 19.88 (s,2P).

^{1}H{^{31}P}-NMR (400 MHz, Deuterium Oxide) δ 3.02 (d, J = 4.6 Hz, 2H), 2.62 (t, J = 7.1 Hz, 1H), 2.55 (m, 4H), 1.62 (m, 4H), 1.48 (2H).

^{13}C NMR (101 MHz, Deuterium Oxide) δ 39.3 (t, J = 124.7 Hz), 32.5, 31.8, 28.1 (t, J = 4.9 Hz), 27.1, 26.6, 23.6.

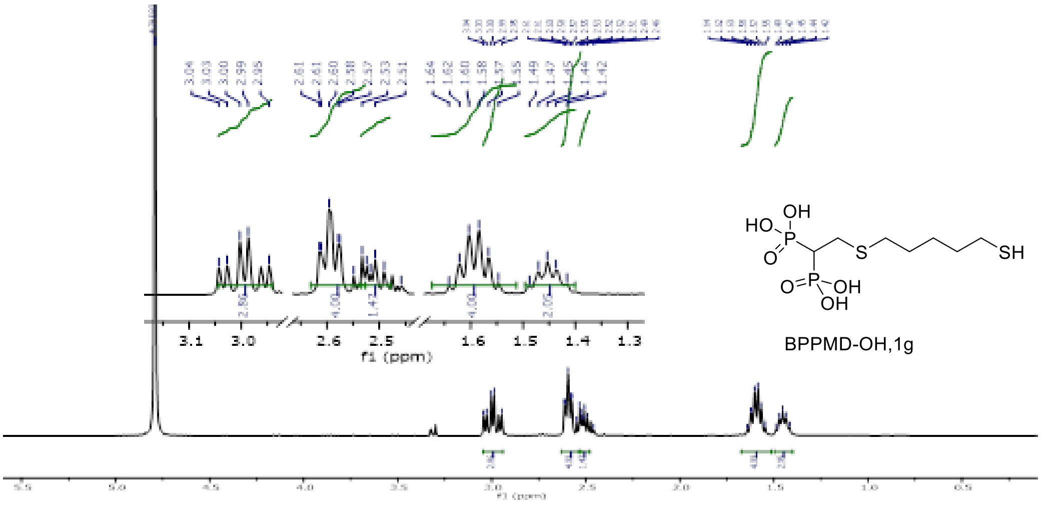

Figure 85. ^{1}H NMR of **1g**.

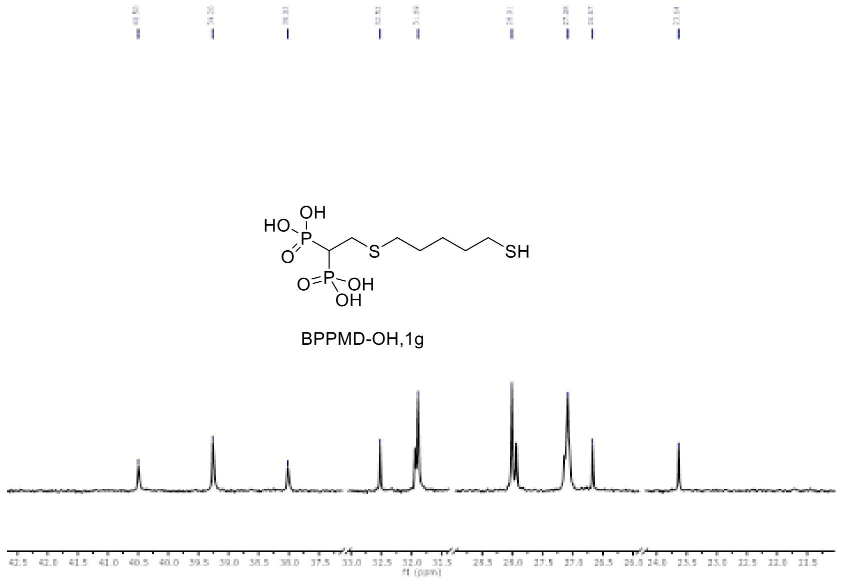

Figure 86. ^{13}C NMR of **1g**.

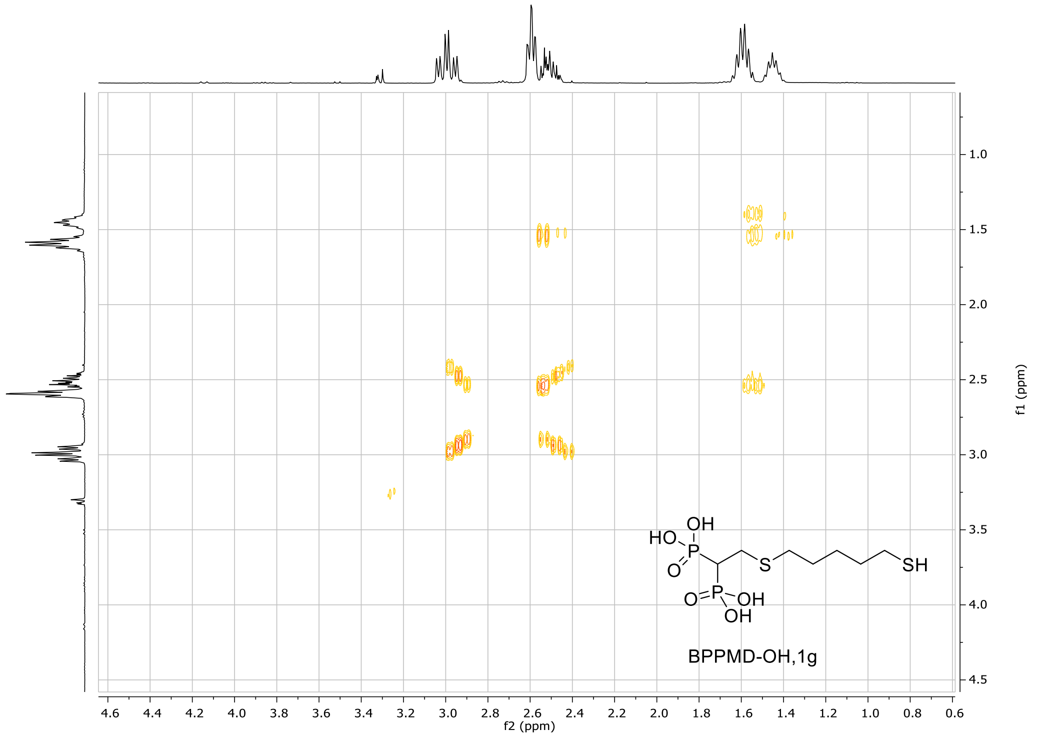

Figure 87. 2D NMR COSY of **1g**.

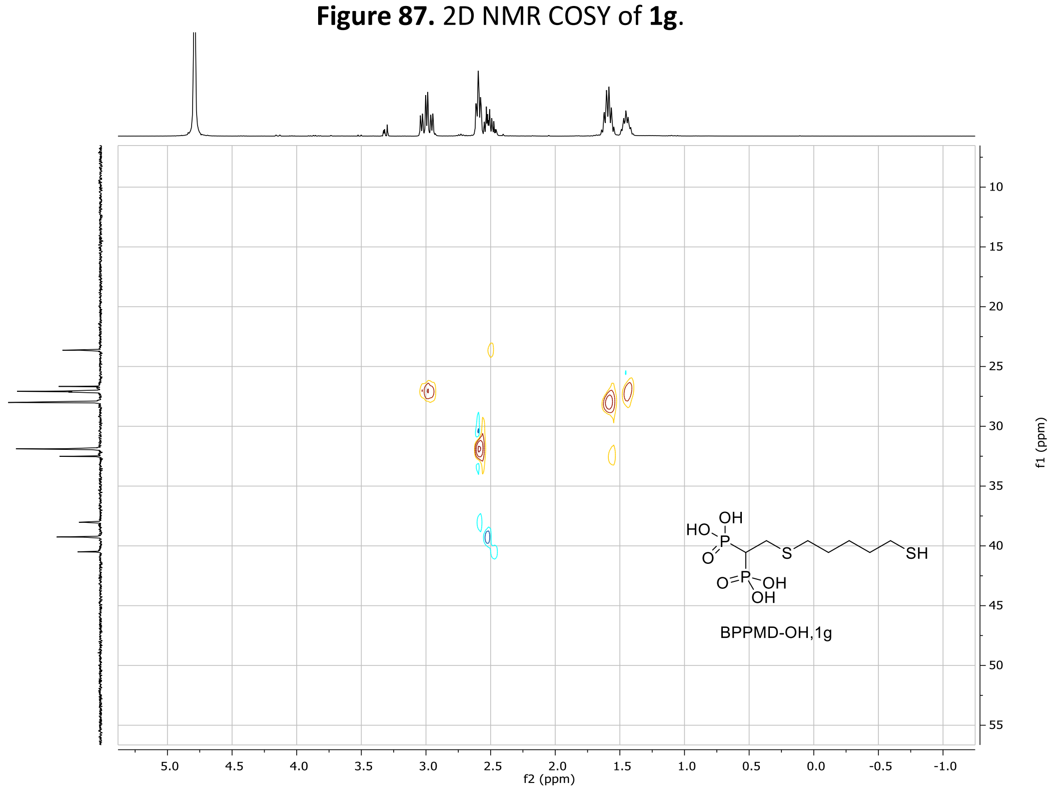

Figure 88. 2D NMR HSQC of **1g**.

7. Functionalization of ZrNPs with BP acids

The bisphosphonic acids functionalized ZrNPs were synthesized by dispersing 50 mg of ZrNPs into 5 mL of pure D_2O or a 25 mM buffer solution in D_2O (pH 4.0-8.8 excluding MES and CAPS due to their strong affinity for the ZrNPs) of 5 mM of the desired BP acid in D_2O in order to have a concentration of 10

mg/ml of ZrNPs. The dispersions were left under continuous stirring 600 rpm for different time periods 15, 30, 60, 120 minutes). Right after, the grafted NPs in the colloidal suspension were isolated by centrifugation for 10 min at 12000 rpm, removing the surnatant solution that was analyzed for the BP content by quantitative NMR applying the Eretic program with respect to a mother solution of the BP used for the loading process.

8. Quantitative NMR determination of Buffers and BPs interaction with ZrNPs

Buffer solutions:

In order to quantitatively determine the interaction between ZrNPs and the buffers, 25 mM solutions of acetic acid / sodium acetate (Acetate), MES, HEPES, TRIZMA and CAPS in D_2O were prepared. ^1H NMR reference analysis for each buffer solution were performed using the BRUKER Advance spectrometer 400 acquiring 32 scans in D_2O and applying solvent suppression (such as not to identify in the spectrum the intense signal of non-deuterated water at 4.79 ppm present in solution). Subsequently, 1 mL of buffer in D_2O and 10 mg of nanoparticles were added respectively in different vials. All the samples were stirred for 24 h using a magnetic stirrer (600 rpm) and at the end they were centrifuged at 12000 rpm for ten minutes. The supernatant was analyzed in NMR using the same procedure described above applying the Eretic program. Once the analyzes on each sample were completed, a pH measurement was performed to verify that the addition of the ZrNPs did not alter the pH of the buffer solutions.

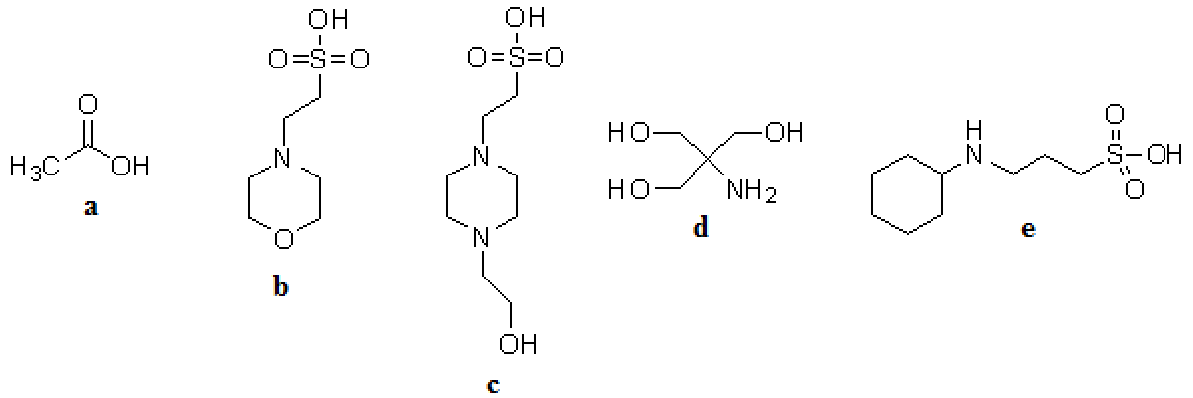

Figure 89. Chemical structure of the buffer species tested: (a) Acetic acid / sodium acetate; (b) MES; (c) HEPES; (d) TRIZMA; (e) CAPS.

Buffer	NMR signal	δ (ppm)	Buffer conc. (mM)	Buffer conc. With ZrNPs (mM)	Delta conc. (%)
**CAPS pH 10.0** 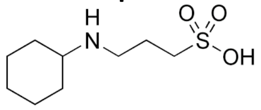	triplet	3.07-2.99	25.00	24.88	0
	multiplet	2.99-2.86	25.00	25.29	-1
	multiplet	2.05-1.90	25.00	24.87	1
	multiplet	1.79-1.67	25.00	24.82	1
	multiplet	1.63-1.54	25.00	24.82	1
	multiplet	1.32-1.04	25.00	25.13	-1
**TRIZMA pH 8.8** 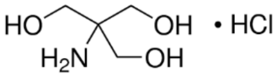	singlet	3.49	25.00	22.61	10
**HEPES pH 7.0** 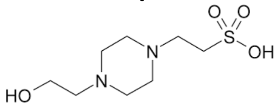	triplet	3.86-3.76	25.00	25.96	-4
	multiplet	2.50-3.50	25.00	25.90	-4

**MES pH 5.5** 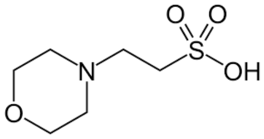	singlet	4.03-3.77	25.00	25.4	-2
**Acetate/Acetic acid pH 4.0** 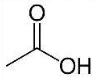	singlet	2.04-1.92	25.00	23.88	4

Table 1. Quantitative ^{1}H NMR determination of the concentration of buffer before and after interaction with the ZrNPs.

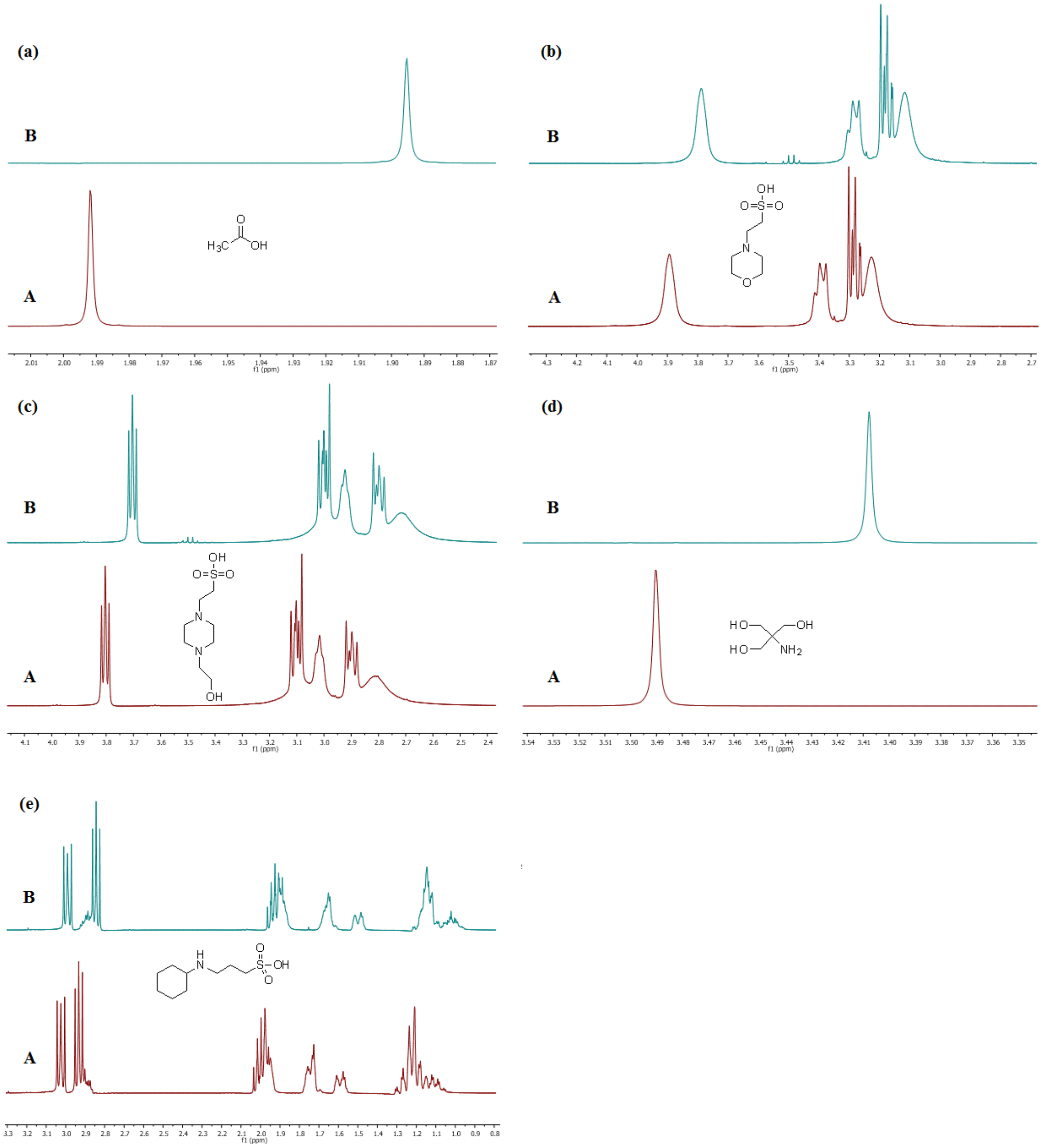

Figure 90. ^{1}H NMR spectra (400MHz, D_2O) for the solutions of the buffers before (A) and after (B) addition of ZrNPs: (a) Acetate 25mM pH 4,0; (b) MES 25mM pH 5.5; (c) HEPES 25mM pH 7.0; (d)TRIZMA 25mM pH 8.8; (e) CAPS 25mM pH 10.0.

BP interaction with ZrNPs:

The concentration of **1a** in solution was determined sampling a fixed amount of the stirred solution, removing the nanoparticles by centrifugation for 10 min at 10000 x g and analyzing the solution by quantitative ^{1}H NMR with respect to the original solution of **1a**.

Because of the time requested for each sample preparation, the first measurement of the residual **1a** concentration in solution after interaction with the ZrNPs was carried out after 12 minutes from mixing, as the shortest time possible from preparation of the sample and data acquisition.

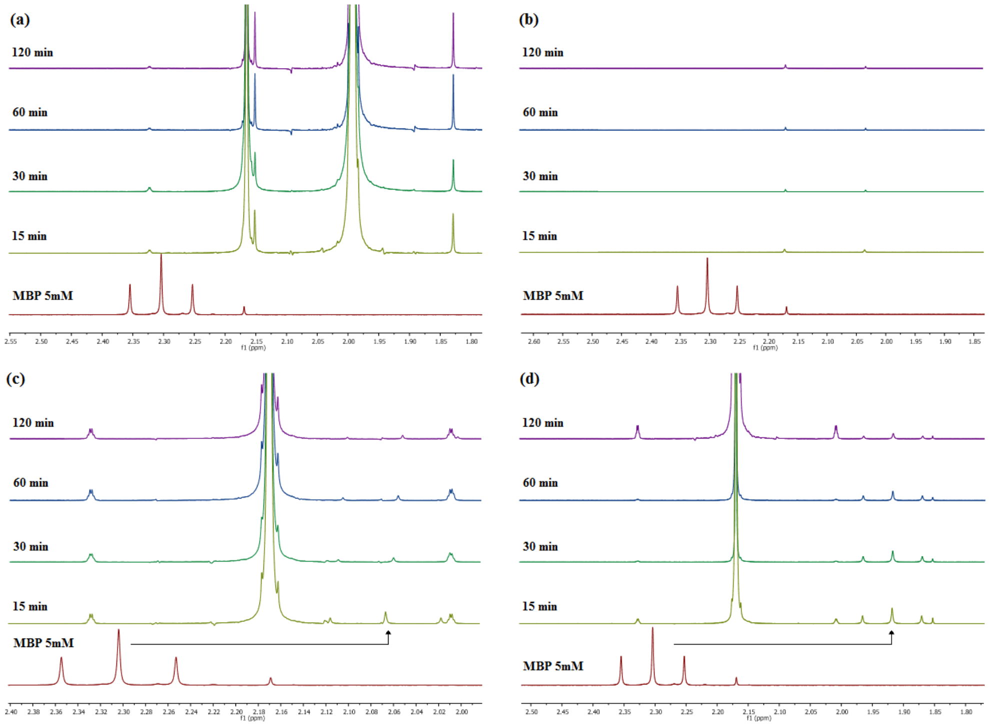

Figure 91. ^{1}H NMR spectra (400MHz, D_2O) of the solutions of **1a** with ZrNPs in different buffer solutions over time: (a) Acetate 25mM pH 4,0; (b) D_2O no buffer; (c) HEPES 25mM pH 7.0; (d)TRIZMA 25mM pH 8.8.

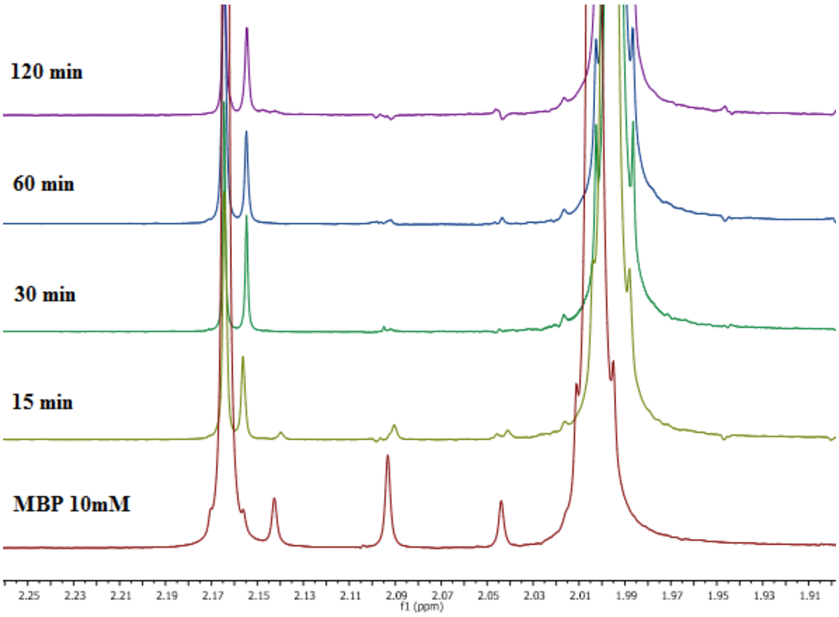

Figure 92. ^{1}H NMR spectra (400MHz, D_2O) of solution of **1a** 10 mM in Acetate buffer 25 mM pH 4.0 after treatment with ZrNPs.

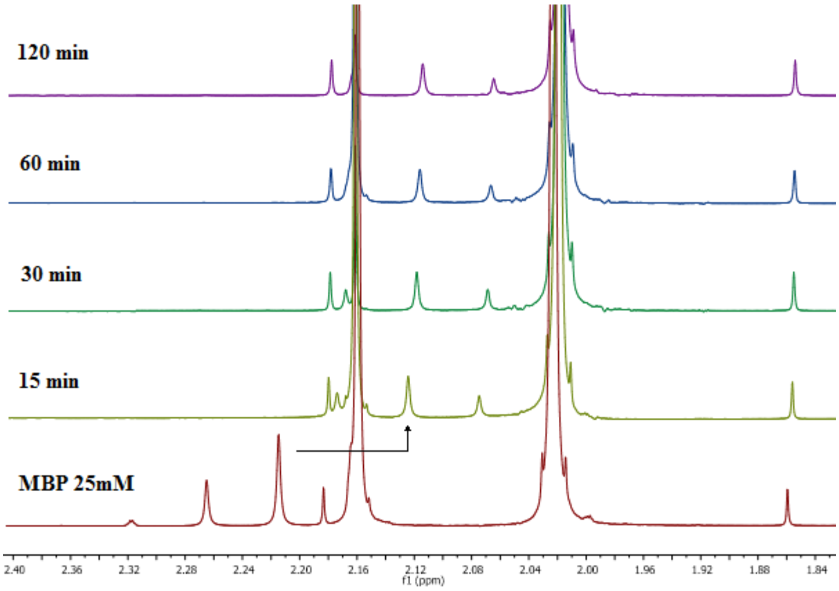

Figure 93. ^{1}H NMR spectra (400MHz, D_2O) of solution of **1a** 20 mM in Acetate buffer 25 mM pH 4.0 after treatment with ZrNPs.

BP	Time (min)	Loading (%)
**1b**	15	78
	30	>98
	60	>98
	120	>98
**1c**	15	>98
	30	>98
	60	>98
	120	>98
**1d**	15	>98

	30	>98
	60	>98
	120	>98
**1e**	15	>98
	30	>98
	60	>98
	120	>98
**1f**	15	>98
	30	>98
	60	>98
	120	>98

Table 2. Loading of BPs at pH 4.0 determined by ^1H quantitative NMR over time.

BP release tests from ZrNPs

The loaded ZrNPs with different BPs were isolated after loading by centrifugation for 10 min at 12000 rpm, washed with milliQ water three times and dried in vacuum for 60 min. Release tests of the BPs from the ZrNPs were carried out dispersing 50 mg of loaded ZrNPs in a 5 mM buffer solution in D_2O (TRIZMA or acetic acid/acetate), stirring the suspension at 600 rpm for different time periods (15-30-60-1440-2880 min) after which 1 mL of suspension was taken. The latter was treated to remove the NPs by centrifugation (10 min at 12000 rpm), isolating the surnatant further analyzed by quantitative ^1H NMR with respect to a mother standard solution of the corresponding BP acids.

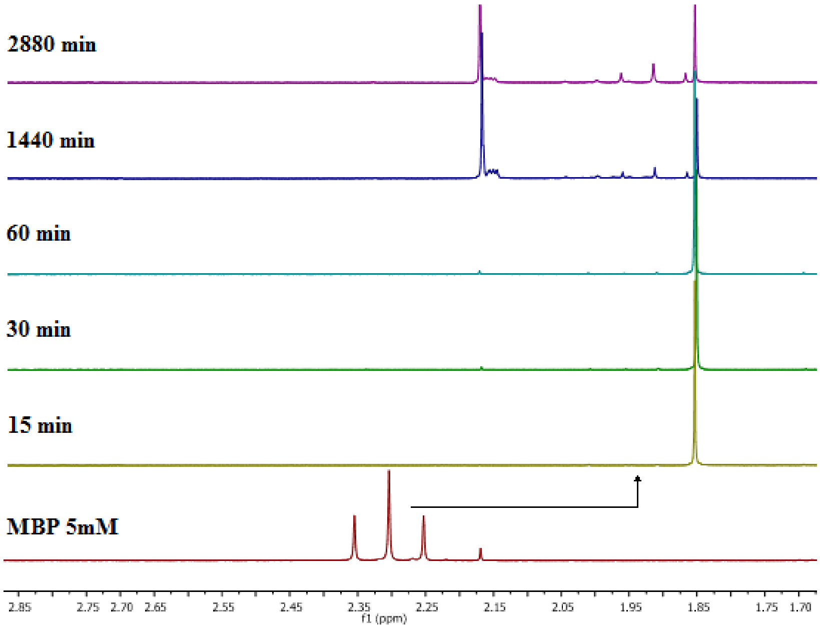

Figure 94. ^1H NMR spectra (400 MHz, D_2O) for the release of **1a** (bottom spectrum for reference) in Trizma buffer 25 mM over time.

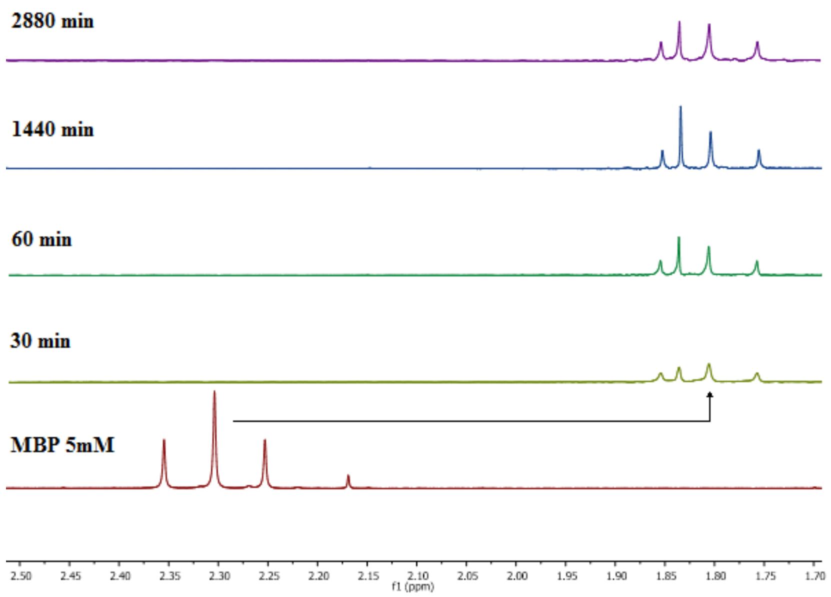

Figure 95. ^{1}H NMR spectra (400 MHz, D_2O) for the release of **1a** (bottom spectrum for reference) in NaOH 100 mM over time.

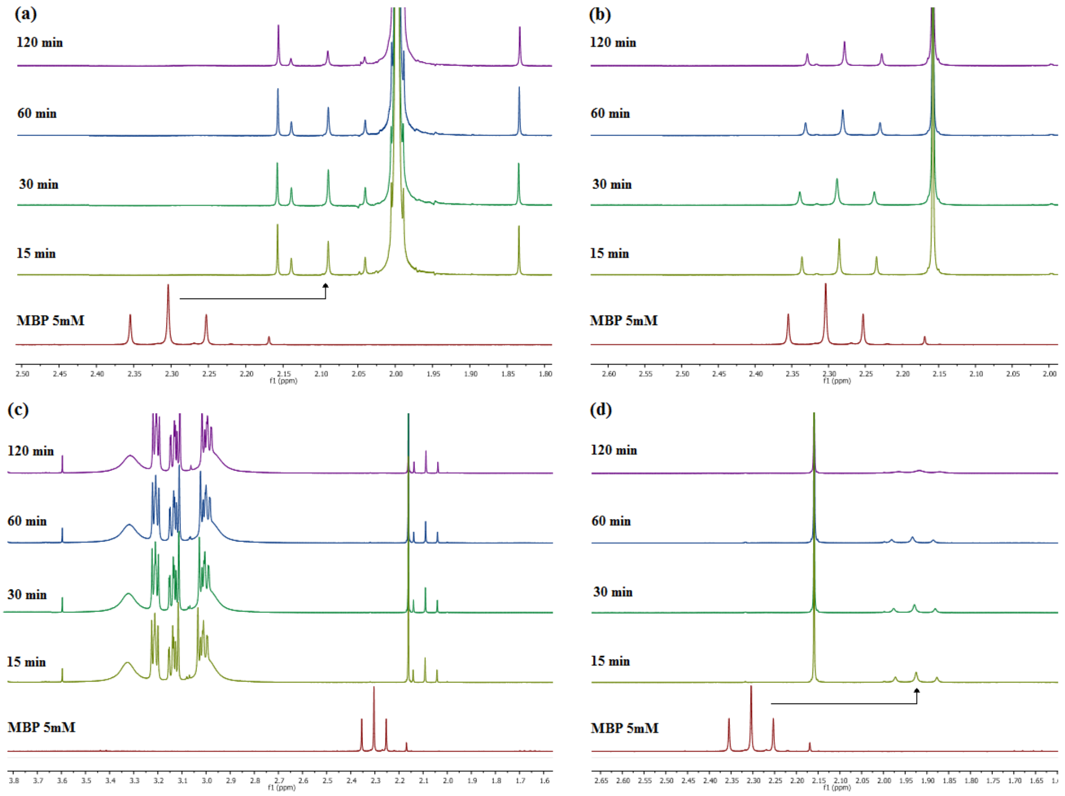

Figure 96. ^{1}H NMR spectra (400 MHz, D_2O) for the functionalization of BiNPs with **1a** 5 mM over time in (a) Acetate 25 mM pH 4.0; (b) D_2O; (c) HEPES 25 mM pH 7.0; (d)TRIZMA 25 mM pH 8.8.

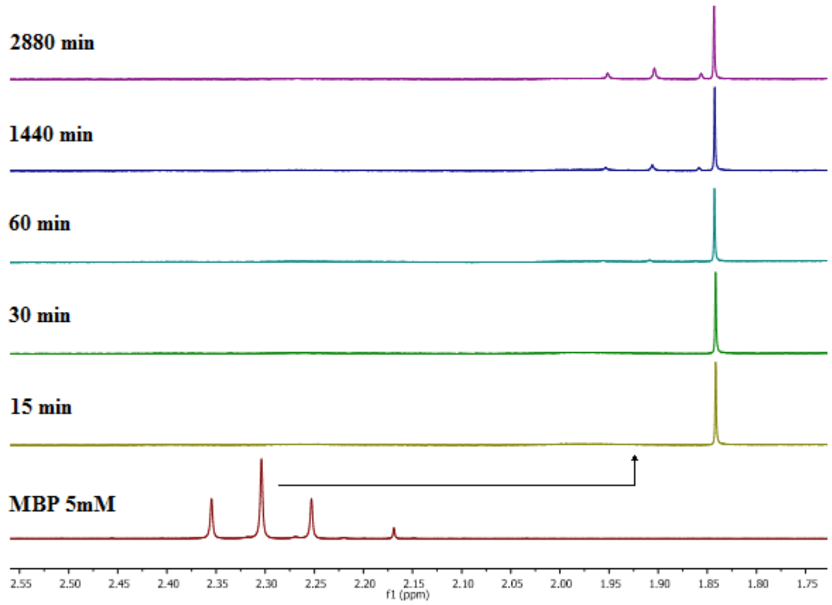

Figure 97. ^{1}H NMR spectra (400 MHz, D_2O) for the release of **1a** (bottom spectrum for reference) from BiNPs in TRIZMA buffer 25 mM pH 8.8.

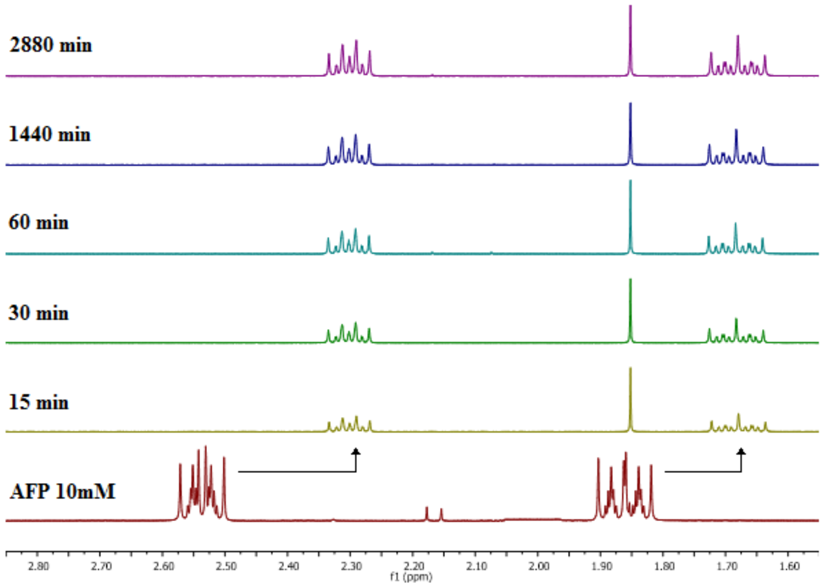

Figure 98. ^{1}H NMR spectra (400 MHz, D_2O) for the release of 2-aminoethylphosphonic acid **6d** (bottom spectrum for reference) from ZrNPs in BPS over time.

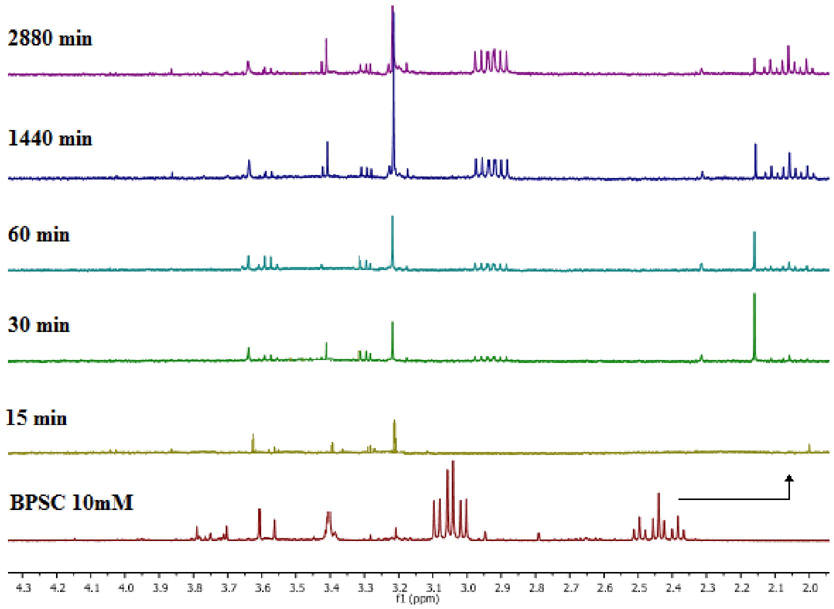

Figure 99. ^{1}H NMR spectra (400 MHz, D_2O) for the release of 3-phosphonopropionic acid **6e** (bottom spectrum for reference) from ZrNPs in BPS over time.

Determination of the pKa values of 1a

A 5 mM solution of **1a** in D_2O was prepared, it was divided into 25 aliquots and to each small amount of HCl 100 mM or NaOH 100 mM were added in order to adjust the pH to cover the range 1-13 checking the final value with a pH meter. Each sample was analyzed by ^{1}H NMR and ^{31}P NMR plotting the *chemical-shift* vs. the pH of the solution. From the plot, the inflection points were determined from first derivative analyses.[S6]

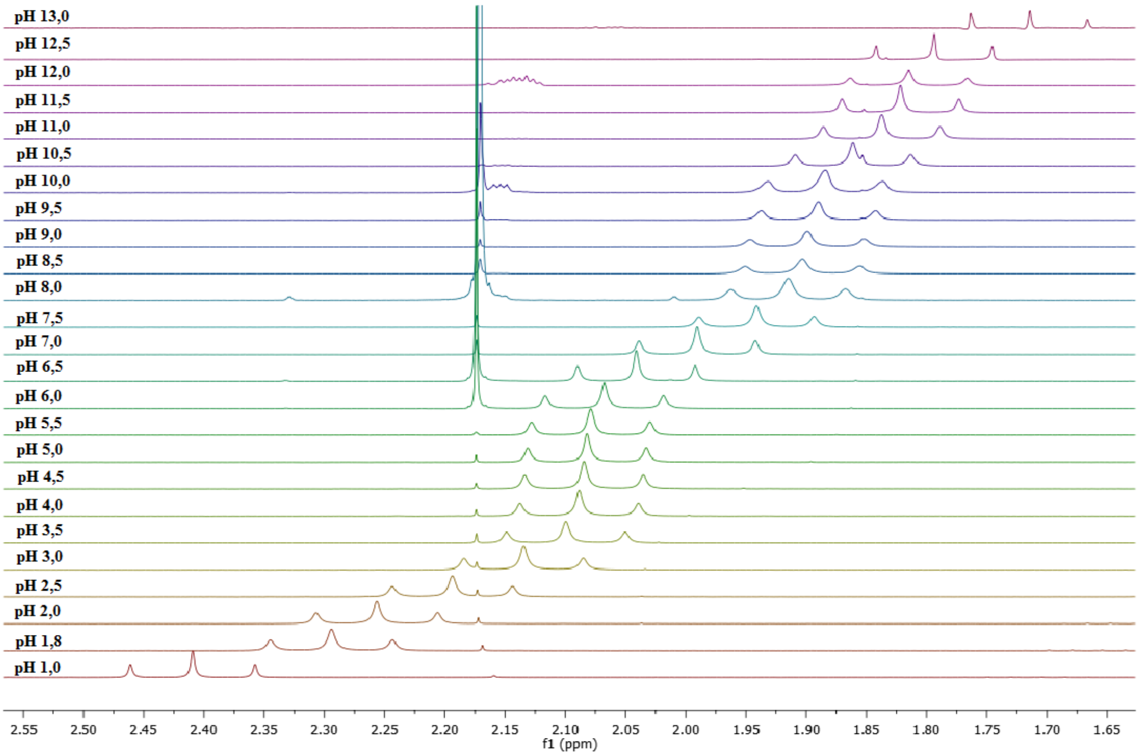

Figure 100. ^{1}H NMR spectra (400MHz, D_2O) of **1a** at different pH values: triplet signal of the methylene protons. Signal at δ 2,17 related to residual acetone.

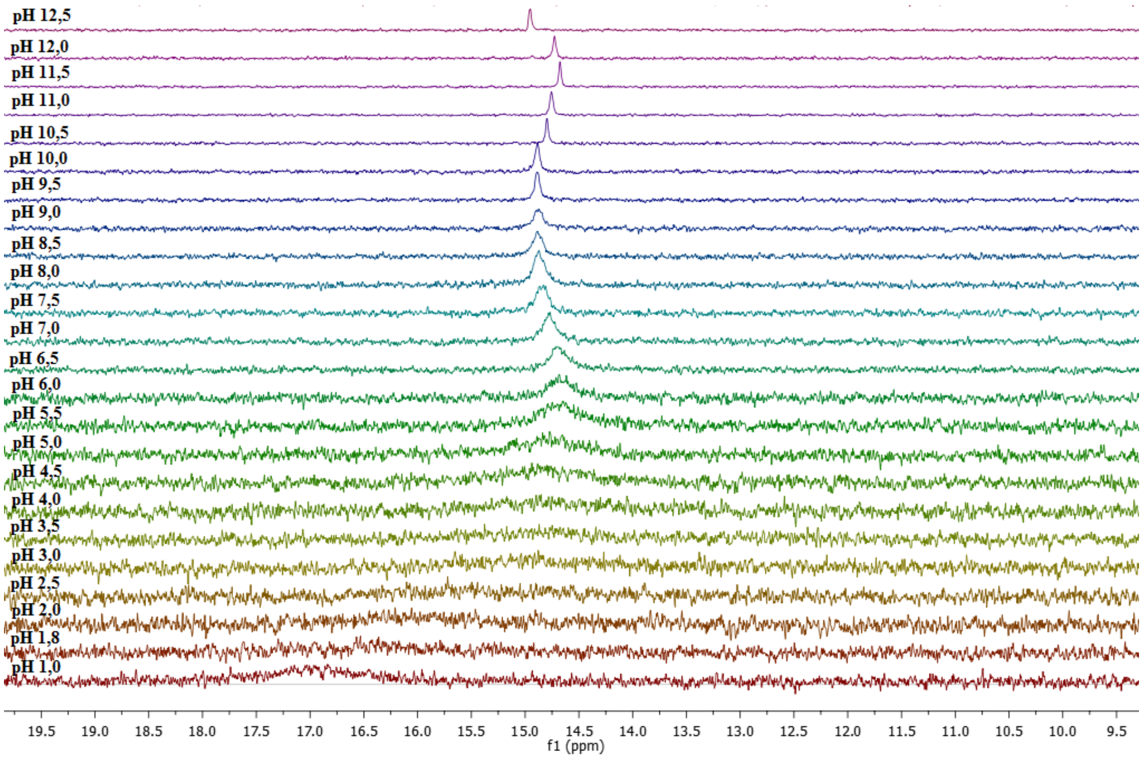

Figure 101. ^{31}P NMR spectra (162 MHz, D_2O) of **1a** at different pH values: singlet signal of the two equivalent P atoms.

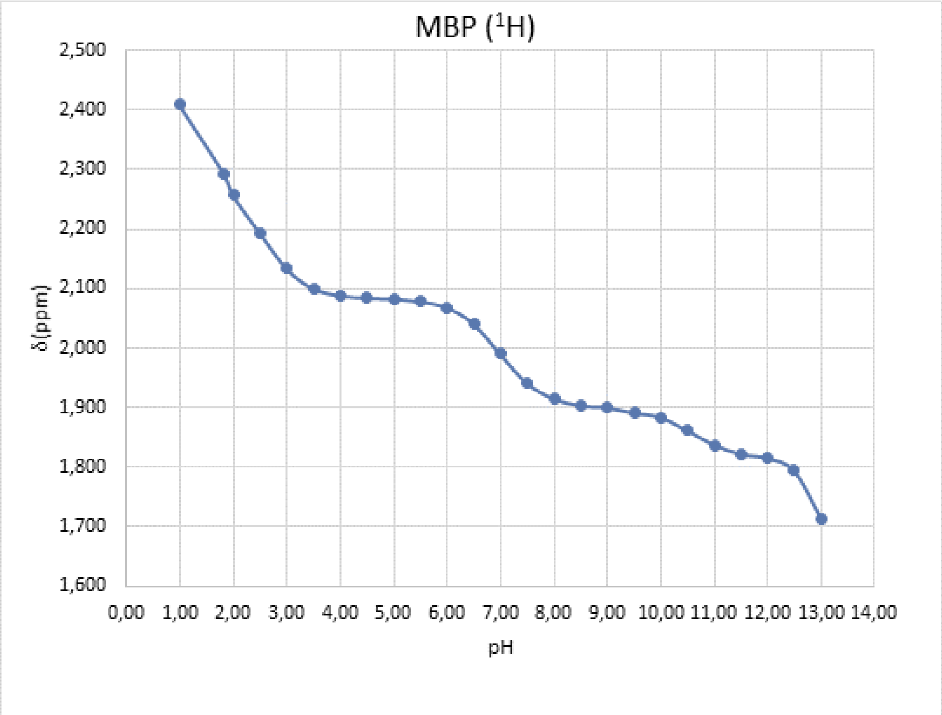

pH	δ (ppm)
1,00	2,409
1,82	2,294
2,00	2,257
2,50	2,194
3,00	2,134
3,50	2,100
4,00	2,088
4,50	2,084
5,00	2,082
5,50	2,078
6,00	2,068
6,50	2,041
7,00	1,990
7,50	1,941
8,00	1,915
8,50	1,903
9,00	1,900
9,50	1,891
10,00	1,884
10,50	1,861
11,00	1,837
11,50	1,822
12,00	1,815
12,50	1,794
13,00	1,714

Figure 102. Plot of the ^{1}H chemical shift *vs.* pH for **1a**.

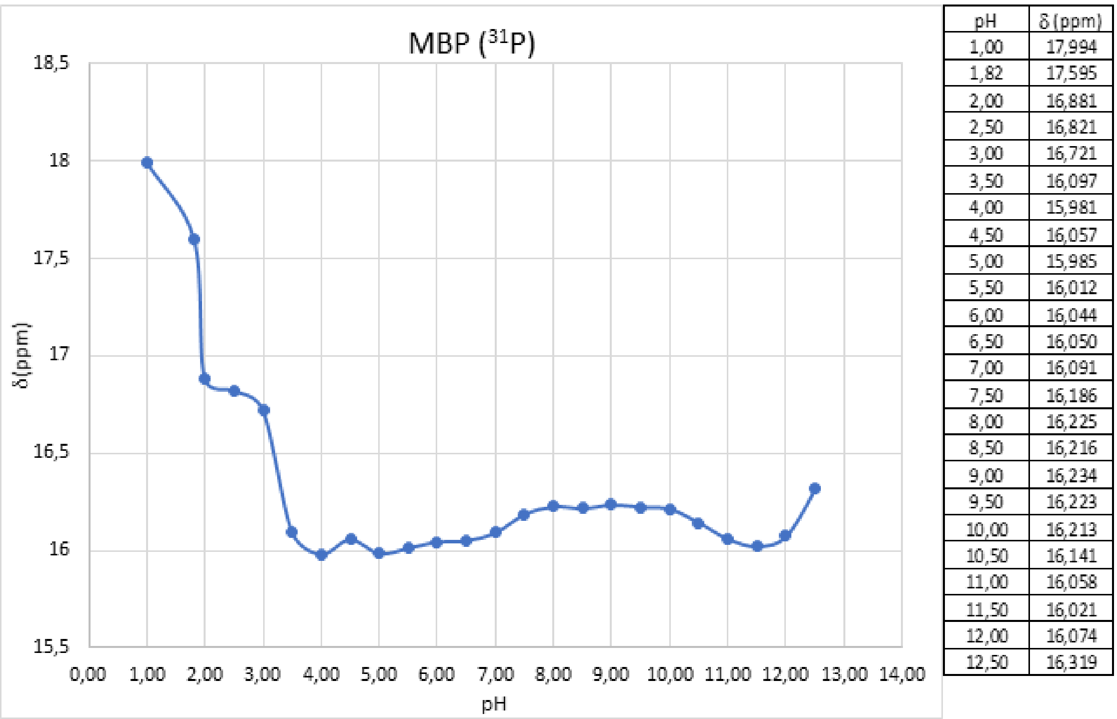

pH	δ (ppm)
1,00	17,994
1,82	17,595
2,00	16,881
2,50	16,821
3,00	16,721
3,50	16,097
4,00	15,981
4,50	16,057
5,00	15,985
5,50	16,012
6,00	16,044
6,50	16,050
7,00	16,091
7,50	16,186
8,00	16,225
8,50	16,216
9,00	16,234
9,50	16,223
10,00	16,213
10,50	16,141
11,00	16,058
11,50	16,021
12,00	16,074
12,50	16,319

Figure 103. Plot of the ^{31}P chemical shift *vs.* pH for **1a**.

9. FTIR spectra of BP acids and functionalized ZrNPs

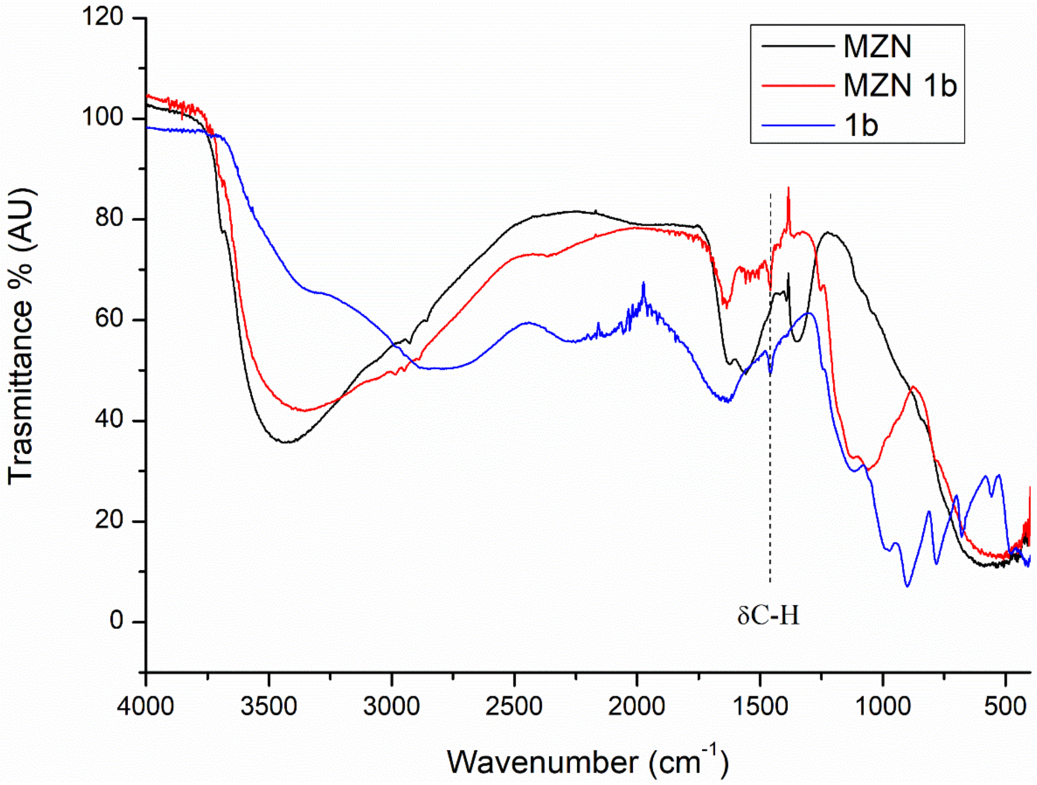

Figure 104. FTIR spectra of pure **1b** (blue) and spectra of MZNs (black) and MZNs **1b** (red).

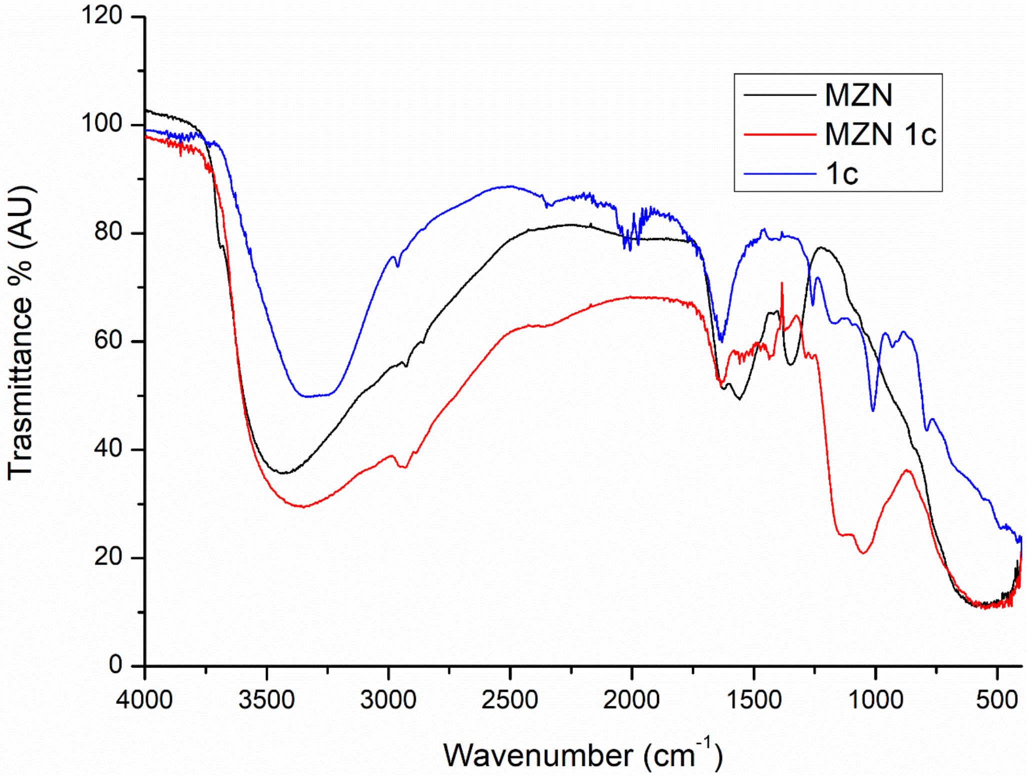

Figure 105. FTIR spectra of pure **1c** (blue) and spectra of MZNs (black) and MZNs **1c** (red).

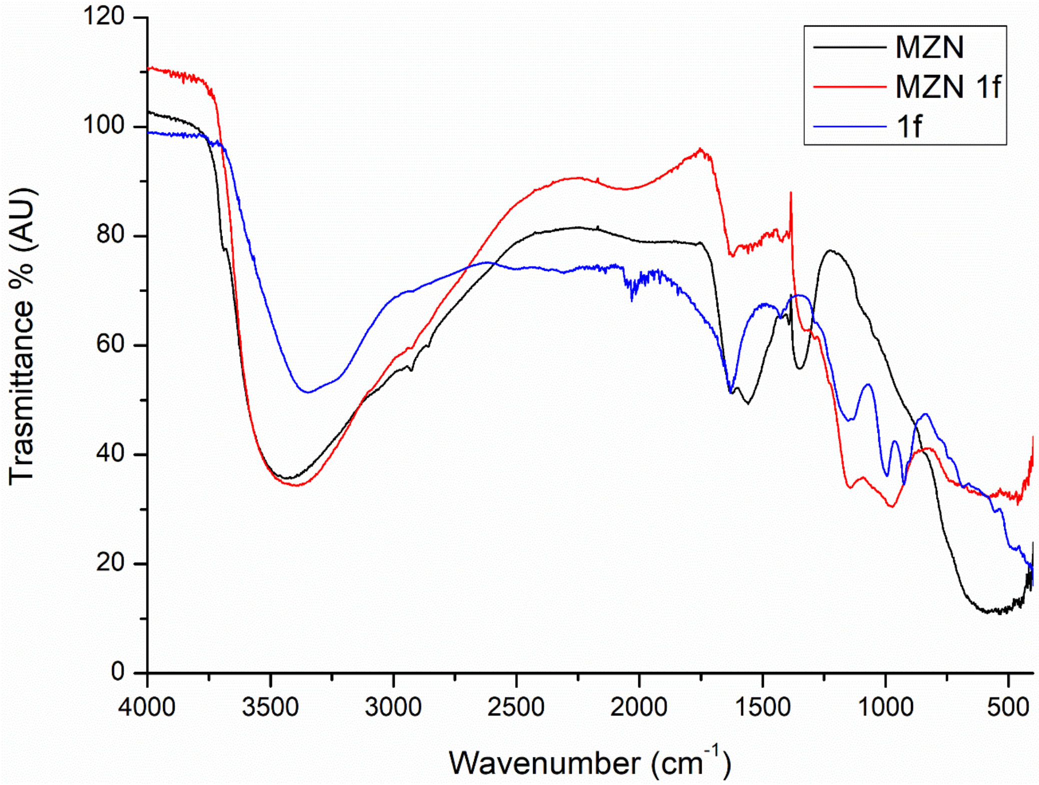

Figure 106. FTIR spectra of pure **1f** (blue) and spectra of MZNs (black) and MZNs **1f** (red).

10. Determination of the grafting density by elemental analyses

The bisphosphonic acids functionalized ZrNPs were synthesized by dispersing 50 mg of ZrNPs into 5 mL of pure D_2O or a 25 mM buffer solution in D_2O at pH 4.0 and 5 mM of the desired BP acid **1a** or **1g** in D_2O in order to have a concentration of 10 mg/ml of ZrNPs. The dispersions were left under continuous stirring 600 rpm for 120 min, then grafted NPs in the colloidal suspension were isolated by centrifugation for 10 min at 12000 rpm, washed with milliQ water three times and dried in vacuum for 60 min. Elemental analyses were determined on duplicates for samples of bare ZrNPs as well as ZrNPs with **1a** and **1g** on a Vario MICRO CHNS instrument.

	C %	H %	N %	S %
Zr NPs	2.9	1.42	0.13	**0.05**
Zr NPs	2.89	1.47	0.13	**0.01**
ZrNP **1a**	3.23	1.49	0.13	**0.11**
ZrNP **1a**	3.31	1.48	0.13	**0.06**
ZrNPs **1g**	6.39	1.72	0.11	**2.02**
ZrNPs **1g**	6.26	1.66	0.11	**2.16**

11. ^{1}H-MAS and ^{1}H-^{31}P HETCOR Solid State NMR spectra of BP functionalized ZrNPs

In the ^{1}H-MAS spectra of all samples (Figure 107), apart 3-phosphonopropanoic acid **6e**, intense signals of hexadecylamine and ethanol used in the synthesis, are present between 0 and 4 ppm. In the spectrum of ZrNPs it is possible to recognize (also with the support of a spectral fitting) an intense signal at about 5 ppm, ascribable to physiosorbed water and Zr-OH and a broad one centered at about 6.5 ppm, likely due to water and Zr-OH strongly hydrogen-bonded. Indeed, after heating in oven, the intensity of the former peak strongly decreases, while the latter remains approximately the same. In all functionalized ZrNPs samples (spectra c, d, f) the region at high chemical shifts is still dominated by the peak at about 6 ppm. The spectrum of pristine **6e** shows an intrinsic worse signal resolution, due to the strongly rigid character of the crystalline solid and the consequently strong homonuclear dipolar couplings. The signal at lowest chemical shift can be ascribed to CH_2 protons, that at about 7 ppm is likely due to tightly bound water, while the broad signal above 10 ppm could be fitted with one peak at about 11 ppm, ascribable to P-OH protons and one at about 14 ppm due to COOH protons.

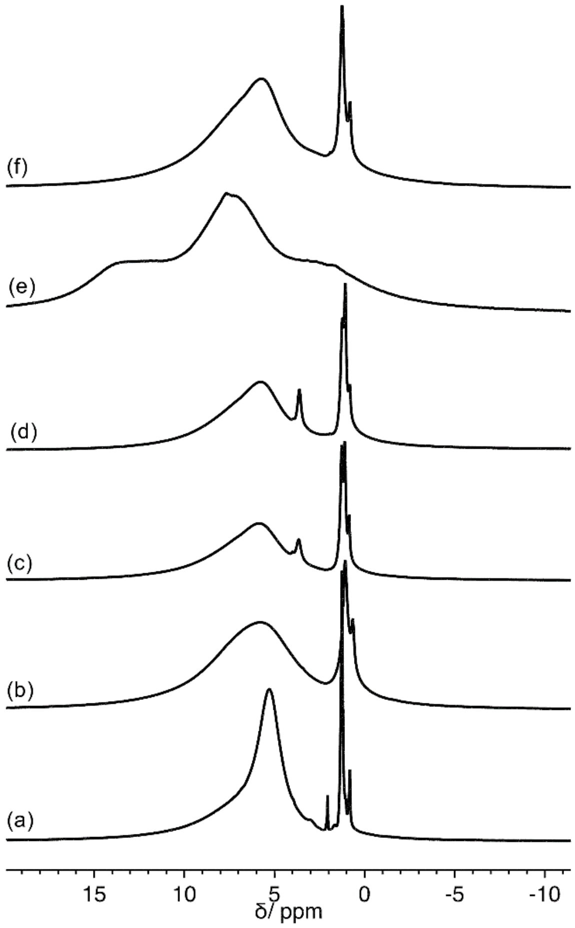

Figure 107. ^{1}H-MAS spectra of (a) ZrNPs (b) ZrNPs after heating at 80°C for 4 h (c) ZrNPs -**1a** (d) ZrNPs -**1b** (e) **6e** (f) ZrNPs -**6e**.

^{1}H-^{31}P HETCOR spectra of ZrNPs -**1a** and **6e**, described in the manuscript, are reported in Figures 108 and 109.

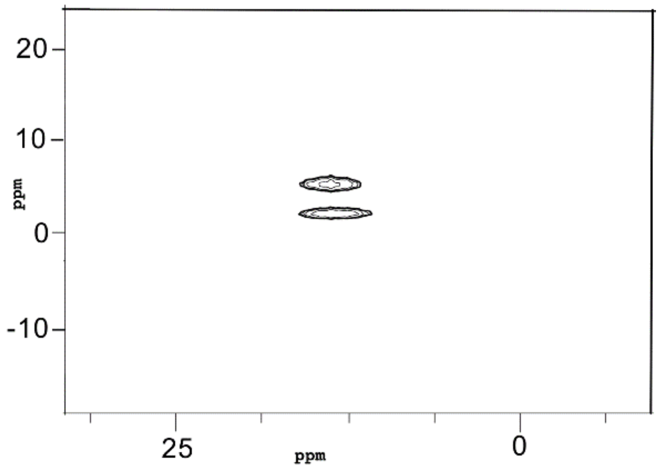

Figure 108. ^{1}H-^{31}P HETCOR spectrum of ZrNPs -**1a**.

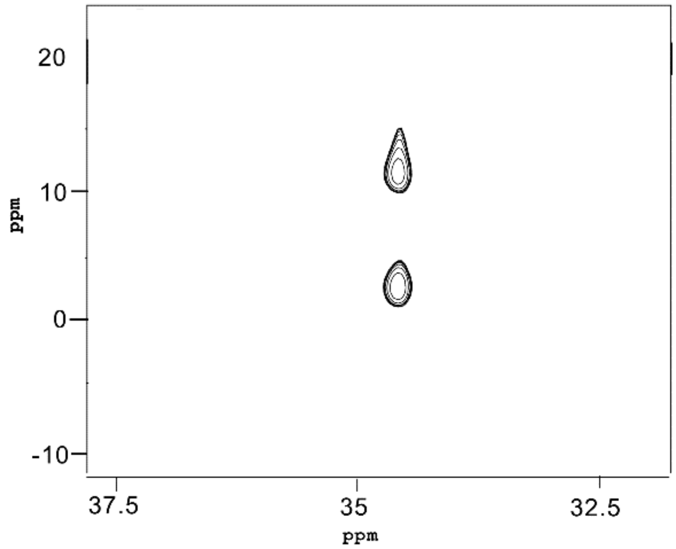

Figure 109. ^{1}H-^{31}P HETCOR spectrum of **6e**.

12. Calculation of loading by ^{1}H NMR and grafting density by Elemental analyses

**Starting Concentration of MBP in sol**	5.0E-03	mol/L	1.0E-02	mol/L	2.5E-02	mol/L
**loading %**	1.0E+02	%	1.0E+02	%	7.0E+01	%
**Final Concentration of MBP in sol**	0.00E+00	mol/L	0.00E+00	mol/L	7.50E-03	mol/L
**Volume of solution**	5.0E-03	L	5.0E-03	L	5.0E-03	L
**Amount of NP used**	**5.00E-02**	g	**5.00E-02**	g	**5.00E-02**	g
**Specific Surface area of ZrNPs**	**1.87E+02**	m2/g	**1.87E+02**	m2/g	**1.87E+02**	m2/g
**Surface area of ZrNPS**	9.35E+00	m2	9.35E+00	m2	9.35E+00	m2
**mol of MBP on ZrNPs**	**2.5E-05**	mol	**5.0E-05**	mol	**8.8E-05**	mol
**Avogrado number, Na**	6.02E+23	molecules	6.02E+23	molecules	6.02E+23	molecules
**Total number of BP molecules on ZrNPs**	1.5E+19	molecules	3.0E+19	molecules	5.3E+19	molecules
**GD moles /m2**	2.7E-06	moles/m2	5.3E-06	moles/m2	9.4E-06	moles/m2
**Grafting density**	1.61E+18	molecules/m2	3.22E+18	molecules/m2	5.64E+18	molecules/m2
**Grafting density in nm2**	1.6	mole-cules/nm2	3.2	molecules/nm2	5.6	molecules/nm2
**GD % in area**	68	% GDA	135	% GDA	237	% GDA
Area BP **1a**	4.2E+01	A2	4.2E+01	A2	4.2E+01	A2
C= mol/V (mol/L*L)						

Calculation of grafting density of MBP with two S atoms on Zr NPs

Starting Concentration of MBP in sol	1.0E-02	mol/L
Final Concentration of MBP in sol	0.00E+00	mol/L
Volume of solution	5.0E-03	L
Amount of NP used	**5.00E-02**	g
Specific Surface area of ZrNPs	**1.87E+02**	m2/g
Surface area of ZrNPS	9.37E+00	m2
mol of MBP on ZrNPs	**1.8E-05**	mol
Avogrado number, Na	6.02E+23	molecules
Total number of BP molecules on ZrNPs	1.1E+19	molecules
GD moles /m2	1.9E-06	moles/m2
Grafting density	1.12E+18	molecules/m2
Grafting density in nm2	1.1	molecules/nm2
GD % in area	47	% GDA
Area BP	4.2E+01	A2

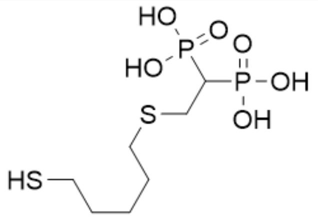

Chemical Formula: $C_7H_{18}O_6P_2S_2$
Exact Mass: 324,00
Molecular Weight: 324,28
m/z: 324.00 (100.0%), 326.00 (9.0%), 325.01 (7.6%), 325.00 (1.6%), 326.01 (1.2%)

13. References

[S1] W. L. F. Armarego, C. Chai in *Purification of Laboratory Chemicals (Seventh Edition)*, Butterworth-Heinemann, Boston, **2013.**

[S2] W. C. Still, M. Kahn, A. Mitra, *J. Org. Chem.*, **1978**, *14*, 2923-2925.

[S3] Naumkin, A. V. NIST X-Ray Photoelectron Spectroscopy Database, Version 4.1; Kraut-Vass, A., Powell, C. J., Gaarenstroom, S. W., Eds.

[S4] T. Radu, C. Iacovita, D. Benea, R. Turcu, Appl. Surf. Sci., **2017**, 405, 337–343.

[S5] G. Sponchia, E. Ambrosi, F. Rizzolio, M. Hadla, A. Del Tedesco, C. Russo Spena, G. Toffoli, P. Riello, A. Benedetti, *J Mater Chem B* **2015,** *3*, 7300-7306.

[S6] J. Bezençon, M. B. Wittwer, B. Cutting, M. Smieško, B. Wagner, M. Kansy, and B. Ernst, *J. Pharm. Biomed. Anal.*, **2014**, *93*, 147–155.
